# Supplementary material for: Associated factors with Premenstrual syndrome and Premenstrual dysphoric disorder among female medical students: A cross-sectional study
Source: PLoS One. 2023 Jan 26;18(1):e0278702. doi: 10.1371/journal.pone.0278702 (PMC9879477; doi:10.1371/journal.pone.0278702)
Supplement: S1 Data — (ZIP) [file pone.0278702.s001.zip › S1 File.docx]

**S1 File. The Premenstrual Syndrome Screening Tool (PSST)^a^**

| **Question** | **Original PSST** | **Vietnamese PSST** |
| --- | --- | --- |
| 1 | Anger/irritability | Tức giận/cáu gắt |
| 2 | Anxiety/tension | Lo âu/Căng thẳng |
| 3 | Tearful/Increased sensitivity to rejection | Hay khóc/tăng nhạy cảm khi bị từ chối |
| 4 | Depressed mood/hoplessness | Khí sắc trầm/tuyệt vọng |
| 5 | Decreased interest in work activities | Giảm hứng thú với các hoạt động trong công việc |
| 6 | Decreased interest in home activeites | Giảm hứng thú với các hoạt động ở nhà |
| 7 | Decreased interest in social activities | Giảm thích thú với các hoạt động xã hội |
| 8 | Difficulty concentrating | Khó tập trung |
| 9 | Fatigue/lack of energy | Mệt mỏi/Thiếu năng lượng |
| 10 | Overeating/food cravings | Ăn quá nhiều/thèm ăn |
| 11 | Insomia | Mất ngủ |
| 12 | Hypersomnia (needing more sleep) | Ngủ nhiều (nhu cầu ngủ nhiều hơn) |
| 13 | Feeling overwhelmed or out of control | Cảm thấy quá tải hoặc mất kiểm soát |
| 14 | Physical symtoms: breast tenderness, headaches, joint/muscle pain, bloading, weight gain | Các triệu chứng thực thể: Căng vú, đau đầu, đau cơ/khớp, chướng bụng, tăng cân. |
| 15 | Your work efficiency or productivity | Hiệu quả hoặc năng suất công việc |
| 16 | Your relationship with coworkers | Các mối quan hệ với đồng nghiệp |
| 17 | Your relationships with your family | Các mối quan hệ với gia đình |
| 18 | Your social life activities | Các hoạt động đời sống xã hội |
| 19 | Your home responsibilities | Tránh nhiệm của bạn đối với gia đình |

^a^Steiner M, Macdougall M, Brown E. The premenstrual symptoms screening tool (PSST) for clinicians. *Arch Womens Ment Health* 2003;**6**:203–209.
