# Supplementary material for: Associated factors with Premenstrual syndrome and Premenstrual dysphoric disorder among female medical students: A cross-sectional study
Source: PLoS One. 2023 Jan 26;18(1):e0278702. doi: 10.1371/journal.pone.0278702 (PMC9879477; doi:10.1371/journal.pone.0278702)
Supplement: S1 Data — (ZIP) [file pone.0278702.s001.zip › S1 Table.docx]

**Table S1. Characteristics of participants with and without follow-up of at least two menstrual cycles**

|  | **Follow-up** | **Non-Follow-up** | **Total** | **P-value** |
| --- | --- | --- | --- | --- |
| n (%) | 302 (70.6) | 126 (29.4) | 428 (100.0) |  |
| **PSST diagnosis, n (%)** |  |  |  |  |
| No PMS & PMDD | 212 (70.2) | 106 (84.1) | 318 (74.3) |  |
| PMS or PMDD | 90 (29.8) | 20 (15.9) | 110 (25.7) | 0.002^2^ |
| **Age (Years), mean (sd)** | 23.24 (3.58) | 24.26 (4.63) | 23.54 (3.94) | 0.014^1^ |
| **Age (Years), median (IQR)** | 22.55 (21.16; 23.93) | 22.87 (20.54; 27.56) | 22.64 (21.01; 24.43) | 0.245^3^ |
| **Hometown n (%)** |  |  |  |  |
| Urban | 79 (26.2) | 36 (28.6) | 115 (26.9) |  |
| Rural | 223 (73.8) | 90 (71.4) | 313 (73.1) | 0.610^2^ |
| **Ethnic group, n (%)** |  |  |  |  |
| Kinh | 290 (96.0) | 125 (99.2) | 415 (97.0) |  |
| Other | 12 (4.0) | 1 (0.8) | 13 (3.0) | 0.120^2^ |
| **Medical specialty, n (%)** |  |  |  |  |
| General medicine | 163 (54.9) | 66 (53.2) | 229 (54.4) |  |
| Preventive medicine | 49 (16.5) | 18 (14.5) | 67 (15.9) |  |
| Traditional medicine | 21 (7.1) | 8 (6.5) | 29 (6.9) |  |
| Pharmacy | 26 (8.8) | 12 (9.7) | 38 (9.0) |  |
| Others (Dentistry, Public health, Medical technician, and Nursing) | 38 (12.8) | 20 (16.1) | 58 (13.8) | 0.893^2^ |
| **ABO blood type^a^, n (%)** |  |  |  |  |
| A | 51 (16.9) | 20 (15.9) | 71 (16.6) |  |
| B | 88 (29.1) | 40 (31.7) | 128 (29.9) |  |
| AB | 19 (6.3) | 6 (4.8) | 25 (5.8) |  |
| O | 128 (42.4) | 50 (39.7) | 178 (41.6) |  |
| Unknown | 16 (5.3) | 10 (7.9) | 26 (6.1) | 0.776^2^ |
| **Rh blood type^a^, n (%)** |  |  |  |  |
| Positive | 269 (89.1) | 98 (77.8) | 367 (85.7) |  |
| Negative | 11 (3.6) | 10 (7.9) | 21 (4.9) |  |
| Unknown | 22 (7.3) | 18 (14.3) | 40 (9.3) | 0.010^2^ |
| **BMI, mean (sd)** | 19.94 (2.16) | 20.40 (2.27) | 20.08 (2.20) | 0.051^1^ |
| **BMI, median (iqr)** | 19.78 (2.60) | 20.25 (2.30) | 20.00 (2.56) | 0.042^3^ |
| **BMI classification^b^, n (%)** |  |  |  |  |
| Underweight | 79 (26.2) | 22 (17.5) | 101 (23.6) |  |
| Normal | 198 (65.6) | 94 (74.6) | 292 (68.2) |  |
| Overweight or Obese | 25 (8.3) | 10 (7.9) | 35 (8.2) | 0.142^2^ |
| **Psychological disorders in 1st degree relatives, n (%)** |  |  |  |  |
| No | 296 (98.0) | 125 (99.2) | 421 (98.4) |  |
| Yes | 6 (2.0) | 1 (0.8) | 7 (1.6) | 0.679^2^ |
| **Alcohol consumption in the last 12 months, n (%)** |  |  |  |  |
| No | 48 (15.9) | 32 (25.4) | 80 (18.7) |  |
| Once per month or less | 226 (74.8) | 82 (65.1) | 308 (72.0) |  |
| More than once per month | 28 (9.3) | 12 (9.5) | 40 (9.3) | 0.066^2^ |
| **Caffein consumption in the last 12 months, n (%)** |  |  |  |  |
| Once a month or less | 158 (52.3) | 68 (54.0) | 226 (52.8) |  |
| 2-3 times per month to 1-3 times per week | 117 (38.7) | 50 (39.7) | 167 (39.0) |  |
| From 4 times per week and above | 27 (8.9) | 8 (6.3) | 35 (8.2) | 0.679^2^ |
| **Physical Activity in the last 7 days^c^, n (%)** |  |  |  |  |
| Low | 145 (48.0) | 65 (51.6) | 210 (49.1) |  |
| Moderate | 108 (35.8) | 38 (30.2) | 146 (34.1) |  |
| Vigorous | 49 (16.2) | 23 (18.3) | 72 (16.8) | 0.529^2^ |
| **Depression level based on PHQ-9, n (%)** |  |  |  |  |
| No or Minimal depression | 111 (36.8) | 54 (42.9) | 165 (38.6) |  |
| Mild depression | 147 (48.7) | 54 (42.9) | 201 (47.0) |  |
| Morderate depression | 44 (14.6) | 18 (14.3) | 62 (14.5) | 0.479^2^ |
| **Smoking, n (%)** |  |  |  |  |
| No | 302 (100.0) | 126 (100.0) | 428 (100.0) | 1.00^2^ |

***^a^*** *Blood type was self-reported.*

***^b^****BMI was classified according to the Asia-Pacific body mass index classifications: Underweight (<18.5 kg/m^2^), Normal (18.5-23 kg/m^2^), Overweight (23-27.5 kg/m^2^) and Obese (> 27.5 kg/m^2^)*

*^c^Physical activity was asscessed by the International Physical Activity Questionnaire (IPAQ-SF)*

**Statistics test***: ^1^Kruskal–Wallis tests, ^2^Fisher exact and ^3^Wilcoxson.*

**Abbreviation:** *BMI – Body mass index; PSST – Premenstrual Syndrome Screening Tools; PMS – Premenstrual syndrome; PMDD – Premenstrual dysphoric disorders; PHQ-9 – Patient health questionare 9*
