# Supplementary material for: Associated factors with Premenstrual syndrome and Premenstrual dysphoric disorder among female medical students: A cross-sectional study
Source: PLoS One. 2023 Jan 26;18(1):e0278702. doi: 10.1371/journal.pone.0278702 (PMC9879477; doi:10.1371/journal.pone.0278702)
Supplement: S1 Data — (ZIP) [file pone.0278702.s001.zip › S2 File.docx]

**S2 File. The Daily Record of Severity Problems (DRSP)^b^**

| **Question** | **Original DRSP** | **Vietnamese DRSP** |
| --- | --- | --- |
| 1 | Felt depressed, sad, down, or blue | Cảm thấy trầm cảm, buồn, tụt dốc, hoặc chán nãn |
| 2 | Felt hopeless | Cảm thấy tuyệt vọng |
| 3 | Felt worthless or guilty | Cảm thấy vô dụng hoặc có lỗi |
| 4 | Felt anxious, tense, keyed up, or on edge | Cảm thấy lo âu, căng thẳng, dễ bị kích thích hoặc dễ cáu. |
| 5 | Had mood swings (e.g., suddenly felt sad or tearful) | Cảm xúc thay đổi (ví dụ., đột ngột cảm thấy buồn hoặc khóc) |
| 6 | Was more sensitive to rejection or feelings were more easily hurt | Nhạy cảm hơn khi bị từ chối hoặc cảm thấy dễ bị tổn thương hơn. |
| 7 | Felt angry, irritable | Cảm thấy tức giận và dễ nỗi cáu. |
| 8 | Had conflicts or problems with people | Có các xung đột hoặc những vấn đề với người khác. |
| 9 | Had less interest in usual activities (e.g., work, school, friends, hobbies) | Giảm hứng thú với những hoạt động thông thường (ví dụ: công việc, trường lớp, bạn bè, sở thích) |
| 10 | Had difficulty concentrating | Khó tập trung |
| 11 | Felt lethargic, tired, fatigued, or had a lack of energy | Cảm thấy lờ đờ, mệt mỏi hoặc cảm thây thiếu năng lượng |
| 12 | Had increased appetite or overate | Tăng cảm giác thèm ăn hoặc ăn quá nhiều. |
| 13 | Had cravings for specific foods | Thèm ăn những thức ăn đặc biệt |
| 14 | Slept more, took naps, found it hard to get up when intended | Ngủ nhiều hơn, ngủ trưa, cảm thấy khó thức dậy hơn khi có ý định. |
| 15 | Had trouble getting to sleep or staying asleep | Khó đi vào giấc ngủ hoặc khó duy trì giấc ngủ |
| 16 | Felt overwhelmed or that I could not cope | Cảm thấy quá tải hoặc không thể đương đầu. |
| 17 | Felt out of control | Cảm thấy mất kiểm soát |
| 18 | Had breast tenderness | Căng tức vú |
| 19 | Had breast swelling, felt bloated, or had weight gain | Sưng vú, chướng bụng, hoặc tăng cân. |
| 20 | Had headache | Đau đầu |
| 21 | Had joint or muscle pain | Đau cơ hoặc khớp |
| 22 | At work, school, home, or in daily routine, at least one of the problems noted above caused reduced productivity or inefficiency | Có ít nhất một trong những vẫn đề ở trên là nguyên nhân gây giảm năng suất hoặc không hiệu quả tại nơi làm việc, trường lớp hoặc những thói quen hàng ngày |
| 23 | At least one of the problems noted above interfered with hobbies or social activities (e.g., avoided or did less) | Ít nhất một trong các vấn đề kể trên làm cản trở sở thích hoặc những hoạt động xã hội (vídụ, né tránh hoặc làm ít hơn) |
| 24 | At least one of the problems noted above interfered with relationships with others | Ít nhất một trong các vấn đề ở trên cản trở mối quan hệ của bạn với người khác. |
| 25 | Menstrual flow: H = heavy, M = medium, L = light or spotting; leave blank for no bleeding | Lượng kinh nguyệt: H= Nhiều, M= trung bình, L= nhẹ hoặc dạng vết; để trống nếu không có chảy máu. |

^b^ Hofmeister S, Bodden S. Premenstrual Syndrome and Premenstrual Dysphoric Disorder. *Am Fam Physician* 2016;**94**:236–240.
