# Supplementary material for: Associated factors with Premenstrual syndrome and Premenstrual dysphoric disorder among female medical students: A cross-sectional study
Source: PLoS One. 2023 Jan 26;18(1):e0278702. doi: 10.1371/journal.pone.0278702 (PMC9879477; doi:10.1371/journal.pone.0278702)
Supplement: S1 Data — (ZIP) [file pone.0278702.s001.zip › S2a Table.docx]

**S2a Table.** Diagnosis of PMS/PMDD based on baseline PSST and C-PASS after at least two menstrual cycles (n=302)

| **PMS/PMDD diagnosis** | **According to C-PASS** | | |  |
| --- | --- | --- | --- | --- |
| **According to baseline PSST** | No PMS&PMDD | PMS | PMDD | **Total** |
| No PMSS&PMDD | 205 | 6 | 1 | 212 |
| PMS | 62 | 24 | 2 | 88 |
| PMDD | 0 | 1 | 1 | 2 |
| **Total** | 267 | 31 | 4 | 302 |

*Abbreviations: PSST (Premenstrual Syndrome Screening Tools); C-PASS (Carolina Premenstrual Assessment Scoring System); PMS (Premenstrual syndrome); PMDD (Premenstrual dysphoric disorders). DSM-V (Diagnostic and Statistical Manual of Mental Disorders – 5^th^ edition)*
