# Supplementary material for: Associated factors with Premenstrual syndrome and Premenstrual dysphoric disorder among female medical students: A cross-sectional study
Source: PLoS One. 2023 Jan 26;18(1):e0278702. doi: 10.1371/journal.pone.0278702 (PMC9879477; doi:10.1371/journal.pone.0278702)
Supplement: S1 Data — (ZIP) [file pone.0278702.s001.zip › S2b Table.docx]

**S2b Table.** Diagnosis of PMS/PMDD based on endline PSST vs C-PASS after at least two menstrual cycles (n=276)*

| **PMS/PMDD diagnosis** | **According to C-PASS** | | |  |
| --- | --- | --- | --- | --- |
| **According to re-test PSST** | No PMS&PMDD | PMS | PMDD | **Total** |
| No PMSS&PMDD | 192 | 12 | 2 | 206 |
| PMS | 48 | 9 | 1 | 58 |
| PMDD | 4 | 7 | 1 | 12 |
| **Total** | 244 | 28 | 4 | 276 |

*Abbreviations: PSST (Premenstrual Syndrome Screening Tools); C-PASS (Carolina Premenstrual Assessment Scoring System); PMS (Premenstrual syndrome); PMDD (Premenstrual dysphoric disorders).*

**There were 276 participants who completed the second PSST at the end of the study.*
