# Supplementary material for: Associated factors with Premenstrual syndrome and Premenstrual dysphoric disorder among female medical students: A cross-sectional study
Source: PLoS One. 2023 Jan 26;18(1):e0278702. doi: 10.1371/journal.pone.0278702 (PMC9879477; doi:10.1371/journal.pone.0278702)
Supplement: S1 Data — (ZIP) [file pone.0278702.s001.zip › S2c Table.docx]

**S2c Table.** Diagnosis of PMS/PMDD based on baseline PSST vs endline PSST (n=276)*

| **Re-test PSST**  **PSST** | **No PMS&PMDD** | **PMS** | **PMDD** | **Total** |
| --- | --- | --- | --- | --- |
| No PMSS&PMDD | 169 | 19 | 1 | 189 |
| PMS | 37 | 39 | 9 | 85 |
| PMDD | 0 | 0 | 2 | 2 |
| **Total** | 206 | 58 | 12 | 276 |

*Abbreviations: PSST (Premenstrual Syndrome Screening Tools); C-PASS (Carolina Premenstrual Assessment Scoring System); PMS (Premenstrual syndrome); PMDD (Premenstrual dysphoric disorders).*

**There were 276 participants who completed the second PSST at the end of the study.*
