# Supplementary material for: Associated factors with Premenstrual syndrome and Premenstrual dysphoric disorder among female medical students: A cross-sectional study
Source: PLoS One. 2023 Jan 26;18(1):e0278702. doi: 10.1371/journal.pone.0278702 (PMC9879477; doi:10.1371/journal.pone.0278702)
Supplement: S1 Data — (ZIP) [file pone.0278702.s001.zip › S3 File.docx]

**S3 File. Literature search syntax**

We conducted the literature review in Pubmed library using this Boolean search term:

((premenstrual syndrome) OR (premenstrual dysphoric disorder) OR (premenstrual disorder) OR (menstrual disorder)) AND (((Premenstrual syndrome screening tool) OR (Daily record of severity of problems) OR (Carolina Premenstrual Assessment Scoring System)) OR ((association factors) OR (caffeine) OR (alcohol) OR (menarche) OR (depression) OR (Blood group) OR (lifestyle) OR (smoking) OR (cigarette) OR (physical activity) OR (exercise)))

The article from 2014 to 2019 had been reviewed (139 articles).
