# Supplementary material for: Associated factors with Premenstrual syndrome and Premenstrual dysphoric disorder among female medical students: A cross-sectional study
Source: PLoS One. 2023 Jan 26;18(1):e0278702. doi: 10.1371/journal.pone.0278702 (PMC9879477; doi:10.1371/journal.pone.0278702)
Supplement: S1 Data — (ZIP) [file pone.0278702.s001.zip › S3 Table.docx]

**S3 Table.** Sensitivity and Specificity of endline PSST compared with C-PASS after at least two menstrual cycles (n=276)*

| **Indicators** | **Point estimate** | **95% Confidental interval** |
| --- | --- | --- |
| Sensitivity (%) | 56.3 | 37.7 - 73.6 |
| Specificity (%) | 78.7 | 73.0 - 83.7 |
| Likelihood ratio (+) | 2.64 | 1.79 - 3.90 |
| Likelihood ratio (-) | 0.56 | 0.37 - 0.83 |
| Positive predictive value (%) | 25.7 | 16.0 - 37.6 |
| Negative predictive value (%) | 93.2 | 88.9 - 96.2 |
| Kappa (PSST vs CPASS) | 0.231 | 0.104 – 0.357 |
| Percent agreement (%) | 76.1 | N/A |

*Abbreviations: PSST (Premenstrual Syndrome Screening Tools); C-PASS (Carolina Premenstrual Assessment Scoring System); PMS (Premenstrual syndrome); PMDD (Premenstrual dysphoric disorders).*
