# Supplementary material for: Associated factors with Premenstrual syndrome and Premenstrual dysphoric disorder among female medical students: A cross-sectional study
Source: PLoS One. 2023 Jan 26;18(1):e0278702. doi: 10.1371/journal.pone.0278702 (PMC9879477; doi:10.1371/journal.pone.0278702)
Supplement: S1 Data — (ZIP) [file pone.0278702.s001.zip › S4 Table.docx]

**S4 Table.** Factor loading after orthogonal rotation

| **Variable** | **Factor 1** | **Factor 2** | **Uniqueness** |
| --- | --- | --- | --- |
| Item 5 (Decreased interest in work activities) |  | 0.8367 | 0.2413 |
| Item 6 (Decreased interest in home activeites) |  | 0.7121 | 0.4544 |
| Item 7 (Decreased interest in social activities) |  | 0.6159 | 0.5129 |
| Item 16 (Your relationship with coworkers) | 0.8453 |  | 0.2290 |
| Item 17 (Your relationships with your family) | 0.7862 |  | 0.3527 |
| Item 18 (Your social life activities) | 0.6322 |  | 0.4545 |
