# Supplementary material for: Associated factors with Premenstrual syndrome and Premenstrual dysphoric disorder among female medical students: A cross-sectional study
Source: PLoS One. 2023 Jan 26;18(1):e0278702. doi: 10.1371/journal.pone.0278702 (PMC9879477; doi:10.1371/journal.pone.0278702)
Supplement: S1 Data — (ZIP) [file pone.0278702.s001.zip › S5 Table.docx]

**S5 Table.** Characteristics of participants by PMS/PMDD status classified by baseline PSST

| **Columns by: PSST diagnosis for PMS & PMDD** | **No PMS & PMDD** | **PMS or PMDD** | **P-value** |
| --- | --- | --- | --- |
| **n (%)** | **212 (70.2)** | **90 (29.8)** |  |
|  | | | |
| **Biological and physical measurement** |  |  |  |
| **Age (Years), mean (sd)** | 23.54 (3.73) | 22.52 (3.11) | 0.023^1^ |
| **ABO blood type^a^, n (%)** |  |  |  |
| A | 38 (74.5) | 13 (25.5) |  |
| B | 60 (68.2) | 28 (31.8) |  |
| AB | 13 (68.4) | 6 (31.6) |  |
| O | 90 (70.3) | 38 (29.7) |  |
| unknown | 11 (68.8) | 5 (31.2) | 0.955^2^ |
| **Rh blood type^a^, n (%)** |  |  |  |
| Positive | 188 (69.9) | 81 (30.1) |  |
| Negative | 7 (63.6) | 4 (36.4) |  |
| unknown | 17 (77.3) | 5 (22.7) | 0.667^2^ |
| **Study year, n (%)** |  |  |  |
| Preclinic (<= 3 years) | 97 (67.8) | 46 (32.2) |  |
| Clinic (> 3 years) | 115 (72.3) | 44 (27.7) | 0.450^2^ |
| **BMI (kg/m2), mean (sd)** | 19.87 (2.23) | 20.11 (1.99) | 0.384^1^ |
| **BMI classification^b^, n (%)** |  |  |  |
| Underweight | 134 (67.7) | 64 (32.3) |  |
| Normal | 60 (75.9) | 19 (24.1) |  |
| Overweight & Obese | 18 (72.0) | 7 (28.0) | 0.397^2^ |
|  | | | |
| **Menstrual status** |  |  |  |
| **Age of menarche (Years), median (IQR)** | 14.00 (13.00; 15.00) | 14.00 (12.00; 14.00) | 0.117^3^ |
| **Late menarche (>= 15 years old), n (%)** | 56 (76.7) | 17 (23.3) | 0.187^2^ |
| **Menstrual days (Days), median (IQR)** | 5.00 (4.00; 5.00) | 5.00 (4.00; 5.25) | 0.513^3^ |
| **Cycle days (Days), median (IQR)** | 30.00 (28.00; 31.00) | 30.00 (28.00; 30.25) | 0.129^3^ |
| **Menstrual blood volumes (ml), median (IQR)** | 70.00 (45.25; 107.88) | 66.75 (41.50; 92.12) | 0.370^3^ |
|  | | | |
| **Obstetric history** |  |  |  |
| **Had >= 1 pregnancy, n (%)** | 18 (85.7) | 3 (14.3) | 0.139^2^ |
| **History of C-section, n (%)** | 6 (75.0) | 2 (25.0) | 1.000^2^ |
| **History of Term births, n (%)** | 16 (84.2) | 3 (15.8) | 0.203^2^ |
| **History of Preterm births, n (%)** | 3 (100.0) | 0 (0.0) | 0.557^2^ |
| **History of Abortions, n (%)** | 2 (50.0) | 2 (50.0) | 0.585^2^ |
|  | | | |
| **Family history** |  |  |  |
| **Psychological disorders in 1st degree relatives^c^, n (%)** | 2 (33.3) | 4 (66.7) | 0.067^2^ |
|  | | | |
| **Lifestyle** |  |  |  |
| **Alcohol consumption in the last 12 months, n (%)** |  |  |  |
| No | 36 (75.0) | 12 (25.0) |  |
| Once per month or less | 160 (70.8) | 66 (29.2) |  |
| More than once per month | 16 (57.1) | 12 (42.9) | 0.233^2^ |
| **Caffeine consumption in the last 12 months, n (%)** |  |  |  |
| Once a month or less | 119 (75.3) | 39 (24.7) |  |
| 2-3 times per month to 1-3 times per week | 80 (68.4) | 37 (31.6) |  |
| From 4 times per week and above | 13 (48.1) | 14 (51.9) | 0.018^2^ |
| **Physical Activity in the last 7 days^d^, n (%)** |  |  |  |
| Low | 108 (74.5) | 37 (25.5) |  |
| Moderate | 74 (68.5) | 34 (31.5) |  |
| Vigorous | 30 (61.2) | 19 (38.8) | 0.191^2^ |
|  | | | |
| **STRESS** |  |  |  |
| **Depression level based on PHQ-9, n (%)** |  |  |  |
| No or Minimal depression | 92 (82.9) | 19 (17.1) |  |
| Mild depression | 103 (70.1) | 44 (29.9) |  |
| Moderate depression | 17 (38.6) | 27 (61.4) |  |
| Severe depression | 92 (82.9) | 19 (17.1) | 0.001^2^ |

***^a^****Blood type was self-reported.*

***^b^****BMI was classified according to the Asia-Pacific body mass index classifications: Underweight (<18.5 kg/m^2^), Normal (18.5-23 kg/m^2^), Overweight (23-27.5 kg/m^2^) and Obese (> 27.5 kg/m^2^)*

***^c^****In the non-PMS/PMDD group, the family history of psychological disorders included Anxiety (n=1) and Bipolar disorder (n=1), while that of the PMS/PMDD group included Depression (n=1), Anxiety (n=2), and Schizophrenia (n=1). The family history was seft-reported in their first-degree relatives.*

*^d^Physical activity was assessed by the International Physical Activity Questionnaire (IPAQ-SF)*

**Statistics test***: ^1^Kruskal–Wallis tests, ^2^Fisher exac and ^3^Wilcoxson.*

**Abbreviation:** *BMI – Body mass index; PSST – Premenstrual Syndrome Screening Tools; PMS – Premenstrual syndrome; PMDD – Premenstrual dysphoric disorders; PHQ-9 – Patient health questionnaire 9.*
