# Supplementary material for: Associated factors with Premenstrual syndrome and Premenstrual dysphoric disorder among female medical students: A cross-sectional study
Source: PLoS One. 2023 Jan 26;18(1):e0278702. doi: 10.1371/journal.pone.0278702 (PMC9879477; doi:10.1371/journal.pone.0278702)
Supplement: S1 Data — (ZIP) [file pone.0278702.s001.zip › S5. File codebook.docx]

---------------------------------------------------------------------------------------------------------------------------------------------------------------------------------------------------------------------------------------------------------------

name: <unnamed>

log: /Users/ngodinhtrieuvy/Documents/PMS /DATA PMS PROJECT 2/Data source/Data results/codebook_data.log

log type: text

opened on: 13 Nov 2022, 15:51:13

.

. * Note: Cx_Qy_Bz or Cx_Qy_Az meaning:

. /// Cx: Cycle x (x range from 1 to 3)

> /// Qy: Question y of CPASS (y range from 1 to 24)

> /// Bz: Day z before menstruation (z range from 1 to 7)

> /// Az: Day z after menstruation (z range from 1 to 10

>

. codebook ,compact

Variable Obs Unique Mean Min Max Label

---------------------------------------------------------------------------------------------------------------------------------------------------------------------------------------------------------------------------------------------------------------

maso 302 302 600.1954 101 1153 Participant ID

C1_Q1_B1 302 7 1.622517 0 6 (sum) C1_Q1_B1

C1_Q1_B2 302 7 1.642384 0 6 (sum) C1_Q1_B2

C1_Q1_B3 302 6 1.662252 1 6 (sum) C1_Q1_B3

C1_Q1_B4 302 6 1.625828 1 6 (sum) C1_Q1_B4

C1_Q1_B5 302 7 1.589404 0 6 (sum) C1_Q1_B5

C1_Q1_B6 302 6 1.65894 1 6 (sum) C1_Q1_B6

C1_Q1_B7 302 7 1.649007 0 6 (sum) C1_Q1_B7

C1_Q1_A1 302 6 1.798013 1 6 (sum) C1_Q1_A1

C1_Q1_A2 302 6 1.850993 1 6 (sum) C1_Q1_A2

C1_Q1_A3 302 6 1.738411 1 6 (sum) C1_Q1_A3

C1_Q1_A4 302 6 1.582781 1 6 (sum) C1_Q1_A4

C1_Q1_A5 302 6 1.466887 1 6 (sum) C1_Q1_A5

C1_Q1_A6 302 6 1.384106 1 6 (sum) C1_Q1_A6

C1_Q1_A7 302 5 1.34106 1 5 (sum) C1_Q1_A7

C1_Q1_A8 302 5 1.334437 1 5 (sum) C1_Q1_A8

C1_Q1_A9 302 6 1.304636 1 6 (sum) C1_Q1_A9

C1_Q1_A10 302 6 1.350993 1 6 (sum) C1_Q1_A10

C1_Q2_B1 302 6 1.364238 0 5 (sum) C1_Q2_B1

C1_Q2_B2 302 7 1.370861 0 6 (sum) C1_Q2_B2

C1_Q2_B3 302 6 1.347682 1 6 (sum) C1_Q2_B3

C1_Q2_B4 302 6 1.400662 1 6 (sum) C1_Q2_B4

C1_Q2_B5 302 7 1.374172 0 6 (sum) C1_Q2_B5

C1_Q2_B6 302 6 1.377483 1 6 (sum) C1_Q2_B6

C1_Q2_B7 302 7 1.413907 0 6 (sum) C1_Q2_B7

C1_Q2_A1 302 6 1.437086 1 6 (sum) C1_Q2_A1

C1_Q2_A2 302 6 1.506623 1 6 (sum) C1_Q2_A2

C1_Q2_A3 302 6 1.490066 1 6 (sum) C1_Q2_A3

C1_Q2_A4 302 6 1.354305 1 6 (sum) C1_Q2_A4

C1_Q2_A5 302 6 1.31457 1 6 (sum) C1_Q2_A5

C1_Q2_A6 302 6 1.268212 1 6 (sum) C1_Q2_A6

C1_Q2_A7 302 5 1.211921 1 6 (sum) C1_Q2_A7

C1_Q2_A8 302 5 1.201987 1 5 (sum) C1_Q2_A8

C1_Q2_A9 302 5 1.182119 1 6 (sum) C1_Q2_A9

C1_Q2_A10 302 6 1.201987 1 6 (sum) C1_Q2_A10

C1_Q3_B1 302 6 1.569536 0 5 (sum) C1_Q3_B1

C1_Q3_B2 302 7 1.5 0 6 (sum) C1_Q3_B2

C1_Q3_B3 302 6 1.543046 1 6 (sum) C1_Q3_B3

C1_Q3_B4 302 6 1.569536 1 6 (sum) C1_Q3_B4

C1_Q3_B5 302 7 1.539735 0 6 (sum) C1_Q3_B5

C1_Q3_B6 302 6 1.493377 1 6 (sum) C1_Q3_B6

C1_Q3_B7 302 7 1.615894 0 6 (sum) C1_Q3_B7

C1_Q3_A1 302 6 1.602649 1 6 (sum) C1_Q3_A1

C1_Q3_A2 302 6 1.569536 1 6 (sum) C1_Q3_A2

C1_Q3_A3 302 6 1.622517 1 6 (sum) C1_Q3_A3

C1_Q3_A4 302 6 1.549669 1 6 (sum) C1_Q3_A4

C1_Q3_A5 302 6 1.440397 1 6 (sum) C1_Q3_A5

C1_Q3_A6 302 5 1.390728 1 5 (sum) C1_Q3_A6

C1_Q3_A7 302 6 1.360927 1 6 (sum) C1_Q3_A7

C1_Q3_A8 302 5 1.251656 1 5 (sum) C1_Q3_A8

C1_Q3_A9 302 5 1.284768 1 6 (sum) C1_Q3_A9

C1_Q3_A10 302 6 1.294702 1 6 (sum) C1_Q3_A10

C1_Q4_B1 302 7 1.735099 0 6 (sum) C1_Q4_B1

C1_Q4_B2 302 7 1.725166 0 6 (sum) C1_Q4_B2

C1_Q4_B3 302 6 1.731788 1 6 (sum) C1_Q4_B3

C1_Q4_B4 302 6 1.721854 1 6 (sum) C1_Q4_B4

C1_Q4_B5 302 7 1.745033 0 6 (sum) C1_Q4_B5

C1_Q4_B6 302 6 1.781457 1 6 (sum) C1_Q4_B6

C1_Q4_B7 302 7 1.89404 0 6 (sum) C1_Q4_B7

C1_Q4_A1 302 6 2.066225 1 6 (sum) C1_Q4_A1

C1_Q4_A2 302 6 2.033113 1 6 (sum) C1_Q4_A2

C1_Q4_A3 302 6 1.92053 1 6 (sum) C1_Q4_A3

C1_Q4_A4 302 6 1.715232 1 6 (sum) C1_Q4_A4

C1_Q4_A5 302 5 1.582781 1 5 (sum) C1_Q4_A5

C1_Q4_A6 302 5 1.450331 1 5 (sum) C1_Q4_A6

C1_Q4_A7 302 5 1.377483 1 5 (sum) C1_Q4_A7

C1_Q4_A8 302 6 1.407285 1 6 (sum) C1_Q4_A8

C1_Q4_A9 302 5 1.407285 1 5 (sum) C1_Q4_A9

C1_Q4_A10 302 6 1.370861 1 6 (sum) C1_Q4_A10

C1_Q5_B1 302 7 1.788079 0 6 (sum) C1_Q5_B1

C1_Q5_B2 302 7 1.721854 0 6 (sum) C1_Q5_B2

C1_Q5_B3 302 6 1.715232 1 6 (sum) C1_Q5_B3

C1_Q5_B4 302 6 1.682119 1 6 (sum) C1_Q5_B4

C1_Q5_B5 302 7 1.751656 0 6 (sum) C1_Q5_B5

C1_Q5_B6 302 6 1.758278 1 6 (sum) C1_Q5_B6

C1_Q5_B7 302 7 1.887417 0 6 (sum) C1_Q5_B7

C1_Q5_A1 302 6 2.072848 1 6 (sum) C1_Q5_A1

C1_Q5_A2 302 6 2 1 6 (sum) C1_Q5_A2

C1_Q5_A3 302 6 1.837748 1 6 (sum) C1_Q5_A3

C1_Q5_A4 302 6 1.649007 1 6 (sum) C1_Q5_A4

C1_Q5_A5 302 5 1.55298 1 6 (sum) C1_Q5_A5

C1_Q5_A6 302 5 1.466887 1 5 (sum) C1_Q5_A6

C1_Q5_A7 302 4 1.334437 1 4 (sum) C1_Q5_A7

C1_Q5_A8 302 6 1.403974 1 6 (sum) C1_Q5_A8

C1_Q5_A9 302 5 1.334437 1 5 (sum) C1_Q5_A9

C1_Q5_A10 302 6 1.374172 1 6 (sum) C1_Q5_A10

C1_Q6_B1 302 7 1.599338 0 6 (sum) C1_Q6_B1

C1_Q6_B2 302 7 1.609272 0 6 (sum) C1_Q6_B2

C1_Q6_B3 302 6 1.592715 1 6 (sum) C1_Q6_B3

C1_Q6_B4 302 6 1.55298 1 6 (sum) C1_Q6_B4

C1_Q6_B5 302 7 1.596026 0 6 (sum) C1_Q6_B5

C1_Q6_B6 302 6 1.576159 1 6 (sum) C1_Q6_B6

C1_Q6_B7 302 7 1.735099 0 6 (sum) C1_Q6_B7

C1_Q6_A1 302 6 1.850993 1 6 (sum) C1_Q6_A1

C1_Q6_A2 302 6 1.807947 1 6 (sum) C1_Q6_A2

C1_Q6_A3 302 6 1.735099 1 6 (sum) C1_Q6_A3

C1_Q6_A4 302 6 1.549669 1 6 (sum) C1_Q6_A4

C1_Q6_A5 302 6 1.433775 1 6 (sum) C1_Q6_A5

C1_Q6_A6 302 5 1.387417 1 5 (sum) C1_Q6_A6

C1_Q6_A7 302 4 1.241722 1 4 (sum) C1_Q6_A7

C1_Q6_A8 302 5 1.264901 1 5 (sum) C1_Q6_A8

C1_Q6_A9 302 5 1.235099 1 5 (sum) C1_Q6_A9

C1_Q6_A10 302 6 1.274834 1 6 (sum) C1_Q6_A10

C1_Q7_B1 302 7 1.645695 0 6 (sum) C1_Q7_B1

C1_Q7_B2 302 7 1.649007 0 6 (sum) C1_Q7_B2

C1_Q7_B3 302 6 1.645695 1 6 (sum) C1_Q7_B3

C1_Q7_B4 302 6 1.622517 1 6 (sum) C1_Q7_B4

C1_Q7_B5 302 7 1.708609 0 6 (sum) C1_Q7_B5

C1_Q7_B6 302 6 1.678808 1 6 (sum) C1_Q7_B6

C1_Q7_B7 302 7 1.788079 0 6 (sum) C1_Q7_B7

C1_Q7_A1 302 6 2.013245 1 6 (sum) C1_Q7_A1

C1_Q7_A2 302 6 1.94702 1 6 (sum) C1_Q7_A2

C1_Q7_A3 302 6 1.804636 1 6 (sum) C1_Q7_A3

C1_Q7_A4 302 6 1.609272 1 6 (sum) C1_Q7_A4

C1_Q7_A5 302 6 1.503311 1 6 (sum) C1_Q7_A5

C1_Q7_A6 302 5 1.384106 1 5 (sum) C1_Q7_A6

C1_Q7_A7 302 5 1.264901 1 5 (sum) C1_Q7_A7

C1_Q7_A8 302 6 1.274834 1 6 (sum) C1_Q7_A8

C1_Q7_A9 302 5 1.254967 1 5 (sum) C1_Q7_A9

C1_Q7_A10 302 4 1.245033 1 4 (sum) C1_Q7_A10

C1_Q8_B1 302 7 1.456954 0 6 (sum) C1_Q8_B1

C1_Q8_B2 302 7 1.470199 0 6 (sum) C1_Q8_B2

C1_Q8_B3 302 6 1.42053 1 6 (sum) C1_Q8_B3

C1_Q8_B4 302 6 1.377483 1 6 (sum) C1_Q8_B4

C1_Q8_B5 302 7 1.443709 0 6 (sum) C1_Q8_B5

C1_Q8_B6 302 6 1.423841 1 6 (sum) C1_Q8_B6

C1_Q8_B7 302 7 1.403974 0 6 (sum) C1_Q8_B7

C1_Q8_A1 302 6 1.476821 1 6 (sum) C1_Q8_A1

C1_Q8_A2 302 6 1.533113 1 6 (sum) C1_Q8_A2

C1_Q8_A3 302 5 1.460265 1 5 (sum) C1_Q8_A3

C1_Q8_A4 302 5 1.427152 1 5 (sum) C1_Q8_A4

C1_Q8_A5 302 5 1.31457 1 5 (sum) C1_Q8_A5

C1_Q8_A6 302 5 1.311258 1 5 (sum) C1_Q8_A6

C1_Q8_A7 302 5 1.192053 1 5 (sum) C1_Q8_A7

C1_Q8_A8 302 5 1.168874 1 6 (sum) C1_Q8_A8

C1_Q8_A9 302 5 1.182119 1 5 (sum) C1_Q8_A9

C1_Q8_A10 302 5 1.172185 1 5 (sum) C1_Q8_A10

C1_Q9_B1 302 7 1.55298 0 6 (sum) C1_Q9_B1

C1_Q9_B2 302 7 1.569536 0 6 (sum) C1_Q9_B2

C1_Q9_B3 302 6 1.652318 1 6 (sum) C1_Q9_B3

C1_Q9_B4 302 6 1.589404 1 6 (sum) C1_Q9_B4

C1_Q9_B5 302 7 1.609272 0 6 (sum) C1_Q9_B5

C1_Q9_B6 302 6 1.57947 1 6 (sum) C1_Q9_B6

C1_Q9_B7 302 7 1.665563 0 6 (sum) C1_Q9_B7

C1_Q9_A1 302 6 1.956954 1 6 (sum) C1_Q9_A1

C1_Q9_A2 302 6 1.986755 1 6 (sum) C1_Q9_A2

C1_Q9_A3 302 6 1.788079 1 6 (sum) C1_Q9_A3

C1_Q9_A4 302 6 1.513245 1 6 (sum) C1_Q9_A4

C1_Q9_A5 302 4 1.466887 1 4 (sum) C1_Q9_A5

C1_Q9_A6 302 5 1.39404 1 5 (sum) C1_Q9_A6

C1_Q9_A7 302 5 1.334437 1 5 (sum) C1_Q9_A7

C1_Q9_A8 302 5 1.291391 1 5 (sum) C1_Q9_A8

C1_Q9_A9 302 4 1.271523 1 4 (sum) C1_Q9_A9

C1_Q9_A10 302 5 1.337748 1 5 (sum) C1_Q9_A10

C1_Q10_B1 302 7 1.887417 0 6 (sum) C1_Q10_B1

C1_Q10_B2 302 7 1.844371 0 6 (sum) C1_Q10_B2

C1_Q10_B3 302 6 1.890728 1 6 (sum) C1_Q10_B3

C1_Q10_B4 302 6 1.940397 1 6 (sum) C1_Q10_B4

C1_Q10_B5 302 7 1.86755 0 6 (sum) C1_Q10_B5

C1_Q10_B6 302 6 1.900662 1 6 (sum) C1_Q10_B6

C1_Q10_B7 302 7 1.930464 0 6 (sum) C1_Q10_B7

C1_Q10_A1 302 6 2.152318 1 6 (sum) C1_Q10_A1

C1_Q10_A2 302 6 2.07947 1 6 (sum) C1_Q10_A2

C1_Q10_A3 302 6 1.956954 1 6 (sum) C1_Q10_A3

C1_Q10_A4 302 6 1.884106 1 6 (sum) C1_Q10_A4

C1_Q10_A5 302 6 1.645695 1 6 (sum) C1_Q10_A5

C1_Q10_A6 302 6 1.615894 1 6 (sum) C1_Q10_A6

C1_Q10_A7 302 6 1.599338 1 6 (sum) C1_Q10_A7

C1_Q10_A8 302 6 1.493377 1 6 (sum) C1_Q10_A8

C1_Q10_A9 302 5 1.523179 1 5 (sum) C1_Q10_A9

C1_Q10_A10 302 6 1.566225 1 6 (sum) C1_Q10_A10

C1_Q11_B1 302 7 1.950331 0 6 (sum) C1_Q11_B1

C1_Q11_B2 302 7 1.910596 0 6 (sum) C1_Q11_B2

C1_Q11_B3 302 6 1.884106 1 6 (sum) C1_Q11_B3

C1_Q11_B4 302 6 1.874172 1 6 (sum) C1_Q11_B4

C1_Q11_B5 302 7 1.903974 0 6 (sum) C1_Q11_B5

C1_Q11_B6 302 6 1.880795 1 6 (sum) C1_Q11_B6

C1_Q11_B7 302 7 2.062914 0 6 (sum) C1_Q11_B7

C1_Q11_A1 302 6 2.516556 1 6 (sum) C1_Q11_A1

C1_Q11_A2 302 6 2.480132 1 6 (sum) C1_Q11_A2

C1_Q11_A3 302 6 2.135762 1 6 (sum) C1_Q11_A3

C1_Q11_A4 302 6 1.903974 1 6 (sum) C1_Q11_A4

C1_Q11_A5 302 6 1.698675 1 6 (sum) C1_Q11_A5

C1_Q11_A6 302 6 1.639073 1 6 (sum) C1_Q11_A6

C1_Q11_A7 302 6 1.612583 1 6 (sum) C1_Q11_A7

C1_Q11_A8 302 5 1.503311 1 5 (sum) C1_Q11_A8

C1_Q11_A9 302 5 1.490066 1 5 (sum) C1_Q11_A9

C1_Q11_A10 302 6 1.576159 1 6 (sum) C1_Q11_A10

C1_Q12_B1 302 7 1.711921 0 6 (sum) C1_Q12_B1

C1_Q12_B2 302 7 1.629139 0 6 (sum) C1_Q12_B2

C1_Q12_B3 302 6 1.678808 1 6 (sum) C1_Q12_B3

C1_Q12_B4 302 6 1.665563 1 6 (sum) C1_Q12_B4

C1_Q12_B5 302 8 1.728477 0 11 (sum) C1_Q12_B5

C1_Q12_B6 302 6 1.745033 1 6 (sum) C1_Q12_B6

C1_Q12_B7 302 7 1.715232 0 6 (sum) C1_Q12_B7

C1_Q12_A1 302 6 1.781457 1 6 (sum) C1_Q12_A1

C1_Q12_A2 302 6 1.715232 1 6 (sum) C1_Q12_A2

C1_Q12_A3 302 6 1.645695 1 6 (sum) C1_Q12_A3

C1_Q12_A4 302 6 1.503311 1 6 (sum) C1_Q12_A4

C1_Q12_A5 302 5 1.390728 1 5 (sum) C1_Q12_A5

C1_Q12_A6 302 5 1.311258 1 5 (sum) C1_Q12_A6

C1_Q12_A7 302 5 1.307947 1 5 (sum) C1_Q12_A7

C1_Q12_A8 302 5 1.221854 1 6 (sum) C1_Q12_A8

C1_Q12_A9 302 4 1.198675 1 4 (sum) C1_Q12_A9

C1_Q12_A10 302 5 1.231788 1 5 (sum) C1_Q12_A10

C1_Q13_B1 302 7 1.582781 0 6 (sum) C1_Q13_B1

C1_Q13_B2 302 6 1.5 0 5 (sum) C1_Q13_B2

C1_Q13_B3 302 6 1.592715 1 6 (sum) C1_Q13_B3

C1_Q13_B4 302 6 1.586093 1 6 (sum) C1_Q13_B4

C1_Q13_B5 302 7 1.629139 0 6 (sum) C1_Q13_B5

C1_Q13_B6 302 6 1.63245 1 6 (sum) C1_Q13_B6

C1_Q13_B7 302 7 1.652318 0 6 (sum) C1_Q13_B7

C1_Q13_A1 302 6 1.711921 1 6 (sum) C1_Q13_A1

C1_Q13_A2 302 6 1.675497 1 6 (sum) C1_Q13_A2

C1_Q13_A3 302 6 1.529801 1 6 (sum) C1_Q13_A3

C1_Q13_A4 302 5 1.423841 1 5 (sum) C1_Q13_A4

C1_Q13_A5 302 6 1.384106 1 6 (sum) C1_Q13_A5

C1_Q13_A6 302 5 1.294702 1 6 (sum) C1_Q13_A6

C1_Q13_A7 302 5 1.281457 1 5 (sum) C1_Q13_A7

C1_Q13_A8 302 6 1.251656 1 6 (sum) C1_Q13_A8

C1_Q13_A9 302 4 1.251656 1 4 (sum) C1_Q13_A9

C1_Q13_A10 302 5 1.248344 1 6 (sum) C1_Q13_A10

C1_Q14_B1 302 7 1.890728 0 6 (sum) C1_Q14_B1

C1_Q14_B2 302 7 1.923841 0 6 (sum) C1_Q14_B2

C1_Q14_B3 302 6 1.983444 1 6 (sum) C1_Q14_B3

C1_Q14_B4 302 6 1.976821 1 6 (sum) C1_Q14_B4

C1_Q14_B5 302 7 1.963576 0 6 (sum) C1_Q14_B5

C1_Q14_B6 302 6 1.937086 1 6 (sum) C1_Q14_B6

C1_Q14_B7 302 7 2.062914 0 6 (sum) C1_Q14_B7

C1_Q14_A1 302 6 2.271523 1 6 (sum) C1_Q14_A1

C1_Q14_A2 302 6 2.291391 1 6 (sum) C1_Q14_A2

C1_Q14_A3 302 6 2.099338 1 6 (sum) C1_Q14_A3

C1_Q14_A4 302 6 1.950331 1 6 (sum) C1_Q14_A4

C1_Q14_A5 302 6 1.741722 1 6 (sum) C1_Q14_A5

C1_Q14_A6 302 6 1.642384 1 6 (sum) C1_Q14_A6

C1_Q14_A7 302 6 1.665563 1 6 (sum) C1_Q14_A7

C1_Q14_A8 302 6 1.506623 1 6 (sum) C1_Q14_A8

C1_Q14_A9 302 6 1.496689 1 6 (sum) C1_Q14_A9

C1_Q14_A10 302 5 1.5 1 5 (sum) C1_Q14_A10

C1_Q15_B1 302 6 1.453642 0 5 (sum) C1_Q15_B1

C1_Q15_B2 302 6 1.437086 0 5 (sum) C1_Q15_B2

C1_Q15_B3 302 6 1.493377 1 6 (sum) C1_Q15_B3

C1_Q15_B4 302 6 1.437086 1 6 (sum) C1_Q15_B4

C1_Q15_B5 302 6 1.437086 0 5 (sum) C1_Q15_B5

C1_Q15_B6 302 6 1.543046 1 6 (sum) C1_Q15_B6

C1_Q15_B7 302 7 1.609272 0 6 (sum) C1_Q15_B7

C1_Q15_A1 302 6 1.655629 1 6 (sum) C1_Q15_A1

C1_Q15_A2 302 6 1.675497 1 6 (sum) C1_Q15_A2

C1_Q15_A3 302 5 1.516556 1 5 (sum) C1_Q15_A3

C1_Q15_A4 302 6 1.496689 1 6 (sum) C1_Q15_A4

C1_Q15_A5 302 5 1.337748 1 6 (sum) C1_Q15_A5

C1_Q15_A6 302 5 1.377483 1 5 (sum) C1_Q15_A6

C1_Q15_A7 302 6 1.31457 1 6 (sum) C1_Q15_A7

C1_Q15_A8 302 5 1.278146 1 5 (sum) C1_Q15_A8

C1_Q15_A9 302 5 1.31457 1 5 (sum) C1_Q15_A9

C1_Q15_A10 302 5 1.311258 1 5 (sum) C1_Q15_A10

C1_Q16_B1 302 7 1.668874 0 6 (sum) C1_Q16_B1

C1_Q16_B2 302 7 1.615894 0 6 (sum) C1_Q16_B2

C1_Q16_B3 302 5 1.655629 1 5 (sum) C1_Q16_B3

C1_Q16_B4 302 6 1.672185 1 6 (sum) C1_Q16_B4

C1_Q16_B5 302 7 1.625828 0 6 (sum) C1_Q16_B5

C1_Q16_B6 302 6 1.652318 1 6 (sum) C1_Q16_B6

C1_Q16_B7 302 7 1.629139 0 6 (sum) C1_Q16_B7

C1_Q16_A1 302 6 1.781457 1 6 (sum) C1_Q16_A1

C1_Q16_A2 302 6 1.758278 1 6 (sum) C1_Q16_A2

C1_Q16_A3 302 6 1.745033 1 6 (sum) C1_Q16_A3

C1_Q16_A4 302 6 1.546358 1 6 (sum) C1_Q16_A4

C1_Q16_A5 302 5 1.47351 1 5 (sum) C1_Q16_A5

C1_Q16_A6 302 5 1.384106 1 5 (sum) C1_Q16_A6

C1_Q16_A7 302 6 1.427152 1 6 (sum) C1_Q16_A7

C1_Q16_A8 302 6 1.39404 1 6 (sum) C1_Q16_A8

C1_Q16_A9 302 5 1.344371 1 5 (sum) C1_Q16_A9

C1_Q16_A10 302 6 1.400662 1 6 (sum) C1_Q16_A10

C1_Q17_B1 302 7 1.602649 0 6 (sum) C1_Q17_B1

C1_Q17_B2 302 7 1.562914 0 6 (sum) C1_Q17_B2

C1_Q17_B3 302 6 1.536424 1 6 (sum) C1_Q17_B3

C1_Q17_B4 302 6 1.539735 1 6 (sum) C1_Q17_B4

C1_Q17_B5 302 7 1.609272 0 6 (sum) C1_Q17_B5

C1_Q17_B6 302 6 1.60596 1 6 (sum) C1_Q17_B6

C1_Q17_B7 302 7 1.589404 0 6 (sum) C1_Q17_B7

C1_Q17_A1 302 6 1.831126 1 6 (sum) C1_Q17_A1

C1_Q17_A2 302 6 1.735099 1 6 (sum) C1_Q17_A2

C1_Q17_A3 302 6 1.65894 1 6 (sum) C1_Q17_A3

C1_Q17_A4 302 6 1.533113 1 6 (sum) C1_Q17_A4

C1_Q17_A5 302 5 1.440397 1 5 (sum) C1_Q17_A5

C1_Q17_A6 302 5 1.347682 1 5 (sum) C1_Q17_A6

C1_Q17_A7 302 5 1.264901 1 5 (sum) C1_Q17_A7

C1_Q17_A8 302 6 1.311258 1 6 (sum) C1_Q17_A8

C1_Q17_A9 302 4 1.241722 1 4 (sum) C1_Q17_A9

C1_Q17_A10 302 6 1.298013 1 6 (sum) C1_Q17_A10

C1_Q18_B1 302 6 1.480132 0 5 (sum) C1_Q18_B1

C1_Q18_B2 302 7 1.559603 0 6 (sum) C1_Q18_B2

C1_Q18_B3 302 6 1.672185 1 6 (sum) C1_Q18_B3

C1_Q18_B4 302 5 1.771523 1 5 (sum) C1_Q18_B4

C1_Q18_B5 302 6 1.847682 0 5 (sum) C1_Q18_B5

C1_Q18_B6 302 6 1.870861 1 6 (sum) C1_Q18_B6

C1_Q18_B7 302 7 1.976821 0 6 (sum) C1_Q18_B7

C1_Q18_A1 302 6 1.970199 1 6 (sum) C1_Q18_A1

C1_Q18_A2 302 6 1.758278 1 6 (sum) C1_Q18_A2

C1_Q18_A3 302 6 1.519868 1 6 (sum) C1_Q18_A3

C1_Q18_A4 302 4 1.331126 1 4 (sum) C1_Q18_A4

C1_Q18_A5 302 5 1.208609 1 5 (sum) C1_Q18_A5

C1_Q18_A6 302 4 1.122517 1 6 (sum) C1_Q18_A6

C1_Q18_A7 302 4 1.086093 1 4 (sum) C1_Q18_A7

C1_Q18_A8 302 5 1.076159 1 5 (sum) C1_Q18_A8

C1_Q18_A9 302 4 1.059603 1 4 (sum) C1_Q18_A9

C1_Q18_A10 302 4 1.066225 1 4 (sum) C1_Q18_A10

C1_Q19_B1 302 7 1.42053 0 6 (sum) C1_Q19_B1

C1_Q19_B2 302 7 1.483444 0 6 (sum) C1_Q19_B2

C1_Q19_B3 302 6 1.572848 1 6 (sum) C1_Q19_B3

C1_Q19_B4 302 5 1.602649 1 5 (sum) C1_Q19_B4

C1_Q19_B5 302 6 1.738411 0 5 (sum) C1_Q19_B5

C1_Q19_B6 302 6 1.718543 1 6 (sum) C1_Q19_B6

C1_Q19_B7 302 7 1.794702 0 6 (sum) C1_Q19_B7

C1_Q19_A1 302 6 2.069536 1 6 (sum) C1_Q19_A1

C1_Q19_A2 302 6 1.834437 1 6 (sum) C1_Q19_A2

C1_Q19_A3 302 6 1.562914 1 6 (sum) C1_Q19_A3

C1_Q19_A4 302 5 1.370861 1 5 (sum) C1_Q19_A4

C1_Q19_A5 302 4 1.195364 1 4 (sum) C1_Q19_A5

C1_Q19_A6 302 4 1.099338 1 4 (sum) C1_Q19_A6

C1_Q19_A7 302 4 1.086093 1 4 (sum) C1_Q19_A7

C1_Q19_A8 302 4 1.082781 1 4 (sum) C1_Q19_A8

C1_Q19_A9 302 4 1.062914 1 4 (sum) C1_Q19_A9

C1_Q19_A10 302 4 1.072848 1 4 (sum) C1_Q19_A10

C1_Q21_B1 302 7 1.483444 0 6 (sum) C1_Q20_B1

C1_Q21_B2 302 6 1.440397 0 5 (sum) C1_Q20_B2

C1_Q21_B3 302 6 1.496689 1 6 (sum) C1_Q20_B3

C1_Q21_B4 302 6 1.466887 1 6 (sum) C1_Q20_B4

C1_Q21_B5 302 7 1.562914 0 6 (sum) C1_Q20_B5

C1_Q21_B6 302 6 1.533113 1 6 (sum) C1_Q20_B6

C1_Q21_B7 302 7 1.65894 0 6 (sum) C1_Q20_B7

C1_Q21_A1 302 6 1.768212 1 6 (sum) C1_Q20_A1

C1_Q21_A2 302 6 1.791391 1 6 (sum) C1_Q20_A2

C1_Q21_A3 302 6 1.622517 1 6 (sum) C1_Q20_A3

C1_Q21_A4 302 5 1.52649 1 5 (sum) C1_Q20_A4

C1_Q21_A5 302 6 1.433775 1 6 (sum) C1_Q20_A5

C1_Q21_A6 302 6 1.400662 1 6 (sum) C1_Q20_A6

C1_Q21_A7 302 5 1.347682 1 6 (sum) C1_Q20_A7

C1_Q21_A8 302 5 1.278146 1 5 (sum) C1_Q20_A8

C1_Q21_A9 302 5 1.317881 1 5 (sum) C1_Q20_A9

C1_Q21_A10 302 5 1.350993 1 5 (sum) C1_Q20_A10

C1_Q20_B1 302 6 1.407285 0 5 (sum) C1_Q21_B1

C1_Q20_B2 302 7 1.44702 0 6 (sum) C1_Q21_B2

C1_Q20_B3 302 5 1.523179 1 5 (sum) C1_Q21_B3

C1_Q20_B4 302 5 1.483444 1 5 (sum) C1_Q21_B4

C1_Q20_B5 302 7 1.556291 0 6 (sum) C1_Q21_B5

C1_Q20_B6 302 6 1.596026 1 6 (sum) C1_Q21_B6

C1_Q20_B7 302 7 1.728477 0 6 (sum) C1_Q21_B7

C1_Q20_A1 302 6 2.152318 1 6 (sum) C1_Q21_A1

C1_Q20_A2 302 6 2.059603 1 6 (sum) C1_Q21_A2

C1_Q20_A3 302 6 1.735099 1 6 (sum) C1_Q21_A3

C1_Q20_A4 302 5 1.55298 1 5 (sum) C1_Q21_A4

C1_Q20_A5 302 5 1.36755 1 5 (sum) C1_Q21_A5

C1_Q20_A6 302 5 1.321192 1 5 (sum) C1_Q21_A6

C1_Q20_A7 302 6 1.288079 1 6 (sum) C1_Q21_A7

C1_Q20_A8 302 5 1.238411 1 5 (sum) C1_Q21_A8

C1_Q20_A9 302 5 1.228477 1 5 (sum) C1_Q21_A9

C1_Q20_A10 302 5 1.221854 1 5 (sum) C1_Q21_A10

C1_Q22_B1 302 6 1.781457 0 5 (sum) C1_Q22_B1

C1_Q22_B2 302 6 1.698675 0 5 (sum) C1_Q22_B2

C1_Q22_B3 302 5 1.705298 1 5 (sum) C1_Q22_B3

C1_Q22_B4 302 6 1.741722 1 6 (sum) C1_Q22_B4

C1_Q22_B5 302 7 1.754967 0 6 (sum) C1_Q22_B5

C1_Q22_B6 302 6 1.708609 1 6 (sum) C1_Q22_B6

C1_Q22_B7 302 7 1.837748 0 6 (sum) C1_Q22_B7

C1_Q22_A1 302 6 2.142384 1 6 (sum) C1_Q22_A1

C1_Q22_A2 302 6 2.072848 1 6 (sum) C1_Q22_A2

C1_Q22_A3 302 6 1.877483 1 6 (sum) C1_Q22_A3

C1_Q22_A4 302 6 1.705298 1 6 (sum) C1_Q22_A4

C1_Q22_A5 302 5 1.476821 1 5 (sum) C1_Q22_A5

C1_Q22_A6 302 5 1.453642 1 5 (sum) C1_Q22_A6

C1_Q22_A7 302 5 1.417219 1 5 (sum) C1_Q22_A7

C1_Q22_A8 302 5 1.380795 1 5 (sum) C1_Q22_A8

C1_Q22_A9 302 5 1.344371 1 5 (sum) C1_Q22_A9

C1_Q22_A10 302 6 1.387417 1 6 (sum) C1_Q22_A10

C1_Q23_B1 302 7 1.592715 0 6 (sum) C1_Q23_B1

C1_Q23_B2 302 6 1.562914 0 5 (sum) C1_Q23_B2

C1_Q23_B3 302 5 1.589404 1 5 (sum) C1_Q23_B3

C1_Q23_B4 302 6 1.559603 1 6 (sum) C1_Q23_B4

C1_Q23_B5 302 7 1.57947 0 6 (sum) C1_Q23_B5

C1_Q23_B6 302 6 1.592715 1 6 (sum) C1_Q23_B6

C1_Q23_B7 302 7 1.655629 0 6 (sum) C1_Q23_B7

C1_Q23_A1 302 6 2.006623 1 6 (sum) C1_Q23_A1

C1_Q23_A2 302 6 1.874172 1 6 (sum) C1_Q23_A2

C1_Q23_A3 302 6 1.705298 1 6 (sum) C1_Q23_A3

C1_Q23_A4 302 6 1.546358 1 6 (sum) C1_Q23_A4

C1_Q23_A5 302 4 1.397351 1 4 (sum) C1_Q23_A5

C1_Q23_A6 302 5 1.357616 1 5 (sum) C1_Q23_A6

C1_Q23_A7 302 5 1.304636 1 5 (sum) C1_Q23_A7

C1_Q23_A8 302 4 1.248344 1 4 (sum) C1_Q23_A8

C1_Q23_A9 302 5 1.268212 1 5 (sum) C1_Q23_A9

C1_Q23_A10 302 4 1.261589 1 4 (sum) C1_Q23_A10

C1_Q24_B1 302 7 1.513245 0 6 (sum) C1_Q24_B1

C1_Q24_B2 302 7 1.509934 0 6 (sum) C1_Q24_B2

C1_Q24_B3 302 6 1.496689 1 6 (sum) C1_Q24_B3

C1_Q24_B4 302 6 1.490066 1 6 (sum) C1_Q24_B4

C1_Q24_B5 302 7 1.523179 0 6 (sum) C1_Q24_B5

C1_Q24_B6 302 6 1.516556 1 6 (sum) C1_Q24_B6

C1_Q24_B7 302 7 1.592715 0 6 (sum) C1_Q24_B7

C1_Q24_A1 302 6 1.778146 1 6 (sum) C1_Q24_A1

C1_Q24_A2 302 6 1.708609 1 6 (sum) C1_Q24_A2

C1_Q24_A3 302 6 1.622517 1 6 (sum) C1_Q24_A3

C1_Q24_A4 302 7 1.433775 0 6 (sum) C1_Q24_A4

C1_Q24_A5 302 5 1.364238 1 5 (sum) C1_Q24_A5

C1_Q24_A6 302 5 1.291391 1 5 (sum) C1_Q24_A6

C1_Q24_A7 302 5 1.281457 1 5 (sum) C1_Q24_A7

C1_Q24_A8 302 4 1.235099 1 4 (sum) C1_Q24_A8

C1_Q24_A9 302 5 1.235099 1 5 (sum) C1_Q24_A9

C1_Q24_A10 302 5 1.225166 1 5 (sum) C1_Q24_A10

C1_Q25_B1 302 1 0 0 0 (sum) C1_Q25_B1

C1_Q25_B2 302 1 0 0 0 (sum) C1_Q25_B2

C1_Q25_B3 302 1 0 0 0 (sum) C1_Q25_B3

C1_Q25_B4 302 2 .0066225 0 2 (sum) C1_Q25_B4

C1_Q25_B5 302 1 0 0 0 (sum) C1_Q25_B5

C1_Q25_B6 302 2 .0016556 0 .5 (sum) C1_Q25_B6

C1_Q25_B7 302 5 .0596026 0 13 (sum) C1_Q25_B7

C1_Q25_A1 302 66 15.01026 0 129.5 (sum) C1_Q25_A1

C1_Q25_A2 302 91 26.57765 0 375 (sum) C1_Q25_A2

C1_Q25_A3 302 80 21.88444 0 125 (sum) C1_Q25_A3

C1_Q25_A4 302 58 11.27649 0 125 (sum) C1_Q25_A4

C1_Q25_A5 302 42 5.975166 0 150 (sum) C1_Q25_A5

C1_Q25_A6 302 27 2.19702 0 85 (sum) C1_Q25_A6

C1_Q25_A7 302 15 .7384106 0 55 (sum) C1_Q25_A7

C1_Q25_A8 302 11 .1788079 0 18 (sum) C1_Q25_A8

C1_Q25_A9 302 3 .0115894 0 1 (sum) C1_Q25_A9

C1_Q25_A10 302 4 .0711921 0 11 (sum) C1_Q25_A10

C2_Q1_B1 302 5 1.357616 1 5 (sum) C2_Q1_B1

C2_Q1_B2 302 6 1.433775 1 6 (sum) C2_Q1_B2

C2_Q1_B3 302 6 1.403974 0 5 (sum) C2_Q1_B3

C2_Q1_B4 302 6 1.410596 0 5 (sum) C2_Q1_B4

C2_Q1_B5 302 5 1.39404 0 4 (sum) C2_Q1_B5

C2_Q1_B6 302 7 1.536424 0 6 (sum) C2_Q1_B6

C2_Q1_B7 302 5 1.615894 1 5 (sum) C2_Q1_B7

C2_Q1_A1 302 6 1.768212 1 6 (sum) C2_Q1_A1

C2_Q1_A2 302 5 1.639073 1 5 (sum) C2_Q1_A2

C2_Q1_A3 302 5 1.592715 1 5 (sum) C2_Q1_A3

C2_Q1_A4 302 7 1.470199 0 6 (sum) C2_Q1_A4

C2_Q1_A5 302 5 1.437086 1 5 (sum) C2_Q1_A5

C2_Q1_A6 302 5 1.34106 1 6 (sum) C2_Q1_A6

C2_Q1_A7 302 6 1.364238 1 6 (sum) C2_Q1_A7

C2_Q1_A8 302 6 1.307947 0 6 (sum) C2_Q1_A8

C2_Q1_A9 302 5 1.311258 1 6 (sum) C2_Q1_A9

C2_Q1_A10 302 4 1.251656 1 4 (sum) C2_Q1_A10

C2_Q2_B1 302 5 1.264901 1 5 (sum) C2_Q2_B1

C2_Q2_B2 302 6 1.307947 1 6 (sum) C2_Q2_B2

C2_Q2_B3 302 6 1.284768 0 5 (sum) C2_Q2_B3

C2_Q2_B4 302 6 1.304636 0 5 (sum) C2_Q2_B4

C2_Q2_B5 302 6 1.344371 0 5 (sum) C2_Q2_B5

C2_Q2_B6 302 7 1.397351 0 6 (sum) C2_Q2_B6

C2_Q2_B7 302 5 1.433775 1 5 (sum) C2_Q2_B7

C2_Q2_A1 302 6 1.496689 1 6 (sum) C2_Q2_A1

C2_Q2_A2 302 5 1.47351 1 5 (sum) C2_Q2_A2

C2_Q2_A3 302 5 1.423841 1 5 (sum) C2_Q2_A3

C2_Q2_A4 302 7 1.34106 0 6 (sum) C2_Q2_A4

C2_Q2_A5 302 5 1.235099 1 5 (sum) C2_Q2_A5

C2_Q2_A6 302 5 1.245033 1 5 (sum) C2_Q2_A6

C2_Q2_A7 302 6 1.251656 1 6 (sum) C2_Q2_A7

C2_Q2_A8 302 5 1.218543 0 4 (sum) C2_Q2_A8

C2_Q2_A9 302 5 1.178808 1 6 (sum) C2_Q2_A9

C2_Q2_A10 302 4 1.172185 1 4 (sum) C2_Q2_A10

C2_Q3_B1 302 5 1.374172 1 5 (sum) C2_Q3_B1

C2_Q3_B2 302 5 1.42053 1 5 (sum) C2_Q3_B2

C2_Q3_B3 302 7 1.387417 0 6 (sum) C2_Q3_B3

C2_Q3_B4 302 6 1.374172 0 5 (sum) C2_Q3_B4

C2_Q3_B5 302 7 1.417219 0 6 (sum) C2_Q3_B5

C2_Q3_B6 302 7 1.509934 0 6 (sum) C2_Q3_B6

C2_Q3_B7 302 6 1.536424 1 6 (sum) C2_Q3_B7

C2_Q3_A1 302 6 1.602649 1 6 (sum) C2_Q3_A1

C2_Q3_A2 302 6 1.589404 1 6 (sum) C2_Q3_A2

C2_Q3_A3 302 6 1.539735 1 6 (sum) C2_Q3_A3

C2_Q3_A4 302 7 1.403974 0 6 (sum) C2_Q3_A4

C2_Q3_A5 302 6 1.390728 1 6 (sum) C2_Q3_A5

C2_Q3_A6 302 5 1.344371 1 5 (sum) C2_Q3_A6

C2_Q3_A7 302 6 1.317881 1 6 (sum) C2_Q3_A7

C2_Q3_A8 302 6 1.271523 0 5 (sum) C2_Q3_A8

C2_Q3_A9 302 6 1.264901 1 6 (sum) C2_Q3_A9

C2_Q3_A10 302 5 1.261589 1 5 (sum) C2_Q3_A10

C2_Q4_B1 302 6 1.503311 1 6 (sum) C2_Q4_B1

C2_Q4_B2 302 6 1.529801 1 6 (sum) C2_Q4_B2

C2_Q4_B3 302 6 1.55298 0 5 (sum) C2_Q4_B3

C2_Q4_B4 302 6 1.509934 0 5 (sum) C2_Q4_B4

C2_Q4_B5 302 6 1.509934 0 5 (sum) C2_Q4_B5

C2_Q4_B6 302 7 1.672185 0 6 (sum) C2_Q4_B6

C2_Q4_B7 302 6 1.804636 1 6 (sum) C2_Q4_B7

C2_Q4_A1 302 6 1.960265 1 6 (sum) C2_Q4_A1

C2_Q4_A2 302 6 1.794702 1 6 (sum) C2_Q4_A2

C2_Q4_A3 302 6 1.695364 1 6 (sum) C2_Q4_A3

C2_Q4_A4 302 7 1.586093 0 6 (sum) C2_Q4_A4

C2_Q4_A5 302 6 1.470199 1 6 (sum) C2_Q4_A5

C2_Q4_A6 302 6 1.437086 1 6 (sum) C2_Q4_A6

C2_Q4_A7 302 6 1.360927 1 6 (sum) C2_Q4_A7

C2_Q4_A8 302 6 1.334437 0 5 (sum) C2_Q4_A8

C2_Q4_A9 302 6 1.31457 1 6 (sum) C2_Q4_A9

C2_Q4_A10 302 5 1.317881 1 5 (sum) C2_Q4_A10

C2_Q5_B1 302 6 1.463576 1 6 (sum) C2_Q5_B1

C2_Q5_B2 302 6 1.543046 1 6 (sum) C2_Q5_B2

C2_Q5_B3 302 6 1.466887 0 5 (sum) C2_Q5_B3

C2_Q5_B4 302 7 1.543046 0 6 (sum) C2_Q5_B4

C2_Q5_B5 302 6 1.549669 0 5 (sum) C2_Q5_B5

C2_Q5_B6 302 7 1.645695 0 6 (sum) C2_Q5_B6

C2_Q5_B7 302 6 1.788079 1 6 (sum) C2_Q5_B7

C2_Q5_A1 302 6 1.966887 1 6 (sum) C2_Q5_A1

C2_Q5_A2 302 6 1.824503 1 6 (sum) C2_Q5_A2

C2_Q5_A3 302 6 1.748344 1 6 (sum) C2_Q5_A3

C2_Q5_A4 302 7 1.52649 0 6 (sum) C2_Q5_A4

C2_Q5_A5 302 6 1.437086 1 6 (sum) C2_Q5_A5

C2_Q5_A6 302 6 1.400662 1 6 (sum) C2_Q5_A6

C2_Q5_A7 302 6 1.34106 1 6 (sum) C2_Q5_A7

C2_Q5_A8 302 5 1.304636 0 4 (sum) C2_Q5_A8

C2_Q5_A9 302 5 1.298013 1 6 (sum) C2_Q5_A9

C2_Q5_A10 302 5 1.268212 1 5 (sum) C2_Q5_A10

C2_Q6_B1 302 6 1.380795 1 6 (sum) C2_Q6_B1

C2_Q6_B2 302 6 1.384106 1 6 (sum) C2_Q6_B2

C2_Q6_B3 302 7 1.410596 0 6 (sum) C2_Q6_B3

C2_Q6_B4 302 7 1.47351 0 6 (sum) C2_Q6_B4

C2_Q6_B5 302 6 1.450331 0 5 (sum) C2_Q6_B5

C2_Q6_B6 302 7 1.480132 0 6 (sum) C2_Q6_B6

C2_Q6_B7 302 6 1.655629 1 6 (sum) C2_Q6_B7

C2_Q6_A1 302 6 1.754967 1 6 (sum) C2_Q6_A1

C2_Q6_A2 302 6 1.668874 1 6 (sum) C2_Q6_A2

C2_Q6_A3 302 6 1.592715 1 6 (sum) C2_Q6_A3

C2_Q6_A4 302 7 1.433775 0 6 (sum) C2_Q6_A4

C2_Q6_A5 302 6 1.350993 1 6 (sum) C2_Q6_A5

C2_Q6_A6 302 6 1.294702 1 6 (sum) C2_Q6_A6

C2_Q6_A7 302 6 1.31457 1 6 (sum) C2_Q6_A7

C2_Q6_A8 302 6 1.245033 0 5 (sum) C2_Q6_A8

C2_Q6_A9 302 5 1.245033 1 6 (sum) C2_Q6_A9

C2_Q6_A10 302 5 1.238411 1 5 (sum) C2_Q6_A10

C2_Q7_B1 302 6 1.456954 1 6 (sum) C2_Q7_B1

C2_Q7_B2 302 6 1.450331 1 6 (sum) C2_Q7_B2

C2_Q7_B3 302 6 1.407285 0 5 (sum) C2_Q7_B3

C2_Q7_B4 302 7 1.490066 0 6 (sum) C2_Q7_B4

C2_Q7_B5 302 6 1.52649 0 5 (sum) C2_Q7_B5

C2_Q7_B6 302 7 1.543046 0 6 (sum) C2_Q7_B6

C2_Q7_B7 302 6 1.738411 1 6 (sum) C2_Q7_B7

C2_Q7_A1 302 6 1.910596 1 6 (sum) C2_Q7_A1

C2_Q7_A2 302 5 1.715232 1 5 (sum) C2_Q7_A2

C2_Q7_A3 302 6 1.625828 1 6 (sum) C2_Q7_A3

C2_Q7_A4 302 6 1.456954 0 5 (sum) C2_Q7_A4

C2_Q7_A5 302 6 1.370861 1 6 (sum) C2_Q7_A5

C2_Q7_A6 302 6 1.337748 1 6 (sum) C2_Q7_A6

C2_Q7_A7 302 5 1.284768 1 5 (sum) C2_Q7_A7

C2_Q7_A8 302 6 1.278146 0 5 (sum) C2_Q7_A8

C2_Q7_A9 302 5 1.274834 1 5 (sum) C2_Q7_A9

C2_Q7_A10 302 5 1.192053 1 5 (sum) C2_Q7_A10

C2_Q8_B1 302 6 1.311258 1 6 (sum) C2_Q8_B1

C2_Q8_B2 302 6 1.258278 1 6 (sum) C2_Q8_B2

C2_Q8_B3 302 7 1.284768 0 6 (sum) C2_Q8_B3

C2_Q8_B4 302 6 1.364238 0 5 (sum) C2_Q8_B4

C2_Q8_B5 302 6 1.321192 0 5 (sum) C2_Q8_B5

C2_Q8_B6 302 7 1.374172 0 6 (sum) C2_Q8_B6

C2_Q8_B7 302 5 1.476821 1 5 (sum) C2_Q8_B7

C2_Q8_A1 302 6 1.493377 1 6 (sum) C2_Q8_A1

C2_Q8_A2 302 6 1.44702 1 6 (sum) C2_Q8_A2

C2_Q8_A3 302 5 1.466887 1 6 (sum) C2_Q8_A3

C2_Q8_A4 302 6 1.317881 0 5 (sum) C2_Q8_A4

C2_Q8_A5 302 5 1.304636 1 6 (sum) C2_Q8_A5

C2_Q8_A6 302 6 1.221854 1 6 (sum) C2_Q8_A6

C2_Q8_A7 302 4 1.211921 1 4 (sum) C2_Q8_A7

C2_Q8_A8 302 5 1.182119 0 4 (sum) C2_Q8_A8

C2_Q8_A9 302 4 1.198675 1 4 (sum) C2_Q8_A9

C2_Q8_A10 302 5 1.188742 1 5 (sum) C2_Q8_A10

C2_Q9_B1 302 6 1.407285 1 6 (sum) C2_Q9_B1

C2_Q9_B2 302 6 1.466887 1 6 (sum) C2_Q9_B2

C2_Q9_B3 302 7 1.470199 0 6 (sum) C2_Q9_B3

C2_Q9_B4 302 6 1.533113 0 5 (sum) C2_Q9_B4

C2_Q9_B5 302 6 1.516556 0 5 (sum) C2_Q9_B5

C2_Q9_B6 302 7 1.57947 0 6 (sum) C2_Q9_B6

C2_Q9_B7 302 6 1.761589 1 6 (sum) C2_Q9_B7

C2_Q9_A1 302 6 1.94702 1 6 (sum) C2_Q9_A1

C2_Q9_A2 302 6 1.884106 1 6 (sum) C2_Q9_A2

C2_Q9_A3 302 6 1.675497 1 6 (sum) C2_Q9_A3

C2_Q9_A4 302 6 1.516556 0 5 (sum) C2_Q9_A4

C2_Q9_A5 302 5 1.456954 1 5 (sum) C2_Q9_A5

C2_Q9_A6 302 6 1.387417 1 6 (sum) C2_Q9_A6

C2_Q9_A7 302 6 1.370861 1 6 (sum) C2_Q9_A7

C2_Q9_A8 302 5 1.324503 0 4 (sum) C2_Q9_A8

C2_Q9_A9 302 5 1.311258 1 6 (sum) C2_Q9_A9

C2_Q9_A10 302 5 1.31457 1 5 (sum) C2_Q9_A10

C2_Q10_B1 302 6 1.701987 1 6 (sum) C2_Q10_B1

C2_Q10_B2 302 5 1.60596 1 5 (sum) C2_Q10_B2

C2_Q10_B3 302 7 1.665563 0 6 (sum) C2_Q10_B3

C2_Q10_B4 302 6 1.672185 0 5 (sum) C2_Q10_B4

C2_Q10_B5 302 7 1.738411 0 6 (sum) C2_Q10_B5

C2_Q10_B6 302 7 1.751656 0 6 (sum) C2_Q10_B6

C2_Q10_B7 302 6 1.937086 1 6 (sum) C2_Q10_B7

C2_Q10_A1 302 6 2.142384 1 6 (sum) C2_Q10_A1

C2_Q10_A2 302 6 1.917219 1 6 (sum) C2_Q10_A2

C2_Q10_A3 302 6 1.86755 1 6 (sum) C2_Q10_A3

C2_Q10_A4 302 7 1.728477 0 6 (sum) C2_Q10_A4

C2_Q10_A5 302 5 1.60596 1 5 (sum) C2_Q10_A5

C2_Q10_A6 302 6 1.562914 1 6 (sum) C2_Q10_A6

C2_Q10_A7 302 6 1.533113 1 6 (sum) C2_Q10_A7

C2_Q10_A8 302 6 1.506623 0 5 (sum) C2_Q10_A8

C2_Q10_A9 302 6 1.506623 1 6 (sum) C2_Q10_A9

C2_Q10_A10 302 5 1.523179 1 5 (sum) C2_Q10_A10

C2_Q11_B1 302 5 1.649007 1 5 (sum) C2_Q11_B1

C2_Q11_B2 302 5 1.655629 1 5 (sum) C2_Q11_B2

C2_Q11_B3 302 6 1.655629 0 5 (sum) C2_Q11_B3

C2_Q11_B4 302 7 1.711921 0 6 (sum) C2_Q11_B4

C2_Q11_B5 302 7 1.738411 0 6 (sum) C2_Q11_B5

C2_Q11_B6 302 7 1.817881 0 6 (sum) C2_Q11_B6

C2_Q11_B7 302 6 1.937086 1 6 (sum) C2_Q11_B7

C2_Q11_A1 302 6 2.254967 1 6 (sum) C2_Q11_A1

C2_Q11_A2 302 6 2.102649 1 6 (sum) C2_Q11_A2

C2_Q11_A3 302 6 1.956954 1 6 (sum) C2_Q11_A3

C2_Q11_A4 302 7 1.754967 0 6 (sum) C2_Q11_A4

C2_Q11_A5 302 6 1.619205 1 6 (sum) C2_Q11_A5

C2_Q11_A6 302 5 1.529801 1 5 (sum) C2_Q11_A6

C2_Q11_A7 302 6 1.513245 1 6 (sum) C2_Q11_A7

C2_Q11_A8 302 6 1.506623 0 5 (sum) C2_Q11_A8

C2_Q11_A9 302 5 1.493377 1 5 (sum) C2_Q11_A9

C2_Q11_A10 302 6 1.443709 1 6 (sum) C2_Q11_A10

C2_Q12_B1 302 6 1.360927 1 6 (sum) C2_Q12_B1

C2_Q12_B2 302 5 1.377483 1 5 (sum) C2_Q12_B2

C2_Q12_B3 302 6 1.413907 0 5 (sum) C2_Q12_B3

C2_Q12_B4 302 7 1.443709 0 6 (sum) C2_Q12_B4

C2_Q12_B5 302 7 1.460265 0 6 (sum) C2_Q12_B5

C2_Q12_B6 302 7 1.413907 0 6 (sum) C2_Q12_B6

C2_Q12_B7 302 6 1.466887 1 6 (sum) C2_Q12_B7

C2_Q12_A1 302 5 1.513245 1 5 (sum) C2_Q12_A1

C2_Q12_A2 302 6 1.536424 1 6 (sum) C2_Q12_A2

C2_Q12_A3 302 5 1.506623 1 5 (sum) C2_Q12_A3

C2_Q12_A4 302 6 1.337748 0 5 (sum) C2_Q12_A4

C2_Q12_A5 302 5 1.304636 1 5 (sum) C2_Q12_A5

C2_Q12_A6 302 5 1.284768 1 5 (sum) C2_Q12_A6

C2_Q12_A7 302 5 1.221854 1 5 (sum) C2_Q12_A7

C2_Q12_A8 302 5 1.221854 0 4 (sum) C2_Q12_A8

C2_Q12_A9 302 5 1.211921 1 5 (sum) C2_Q12_A9

C2_Q12_A10 302 5 1.228477 1 5 (sum) C2_Q12_A10

C2_Q13_B1 302 6 1.377483 1 6 (sum) C2_Q13_B1

C2_Q13_B2 302 5 1.433775 1 5 (sum) C2_Q13_B2

C2_Q13_B3 302 6 1.490066 0 5 (sum) C2_Q13_B3

C2_Q13_B4 302 6 1.430464 0 5 (sum) C2_Q13_B4

C2_Q13_B5 302 7 1.506623 0 6 (sum) C2_Q13_B5

C2_Q13_B6 302 7 1.44702 0 6 (sum) C2_Q13_B6

C2_Q13_B7 302 5 1.523179 1 5 (sum) C2_Q13_B7

C2_Q13_A1 302 5 1.589404 1 5 (sum) C2_Q13_A1

C2_Q13_A2 302 6 1.52649 1 6 (sum) C2_Q13_A2

C2_Q13_A3 302 6 1.453642 1 6 (sum) C2_Q13_A3

C2_Q13_A4 302 7 1.39404 0 6 (sum) C2_Q13_A4

C2_Q13_A5 302 5 1.301325 1 5 (sum) C2_Q13_A5

C2_Q13_A6 302 6 1.274834 1 6 (sum) C2_Q13_A6

C2_Q13_A7 302 4 1.218543 1 4 (sum) C2_Q13_A7

C2_Q13_A8 302 5 1.231788 0 4 (sum) C2_Q13_A8

C2_Q13_A9 302 5 1.195364 1 5 (sum) C2_Q13_A9

C2_Q13_A10 302 6 1.264901 1 6 (sum) C2_Q13_A10

C2_Q14_B1 302 6 1.629139 1 6 (sum) C2_Q14_B1

C2_Q14_B2 302 5 1.619205 1 5 (sum) C2_Q14_B2

C2_Q14_B3 302 7 1.672185 0 6 (sum) C2_Q14_B3

C2_Q14_B4 302 7 1.761589 0 6 (sum) C2_Q14_B4

C2_Q14_B5 302 7 1.731788 0 6 (sum) C2_Q14_B5

C2_Q14_B6 302 7 1.758278 0 6 (sum) C2_Q14_B6

C2_Q14_B7 302 6 1.907285 1 6 (sum) C2_Q14_B7

C2_Q14_A1 302 6 2.072848 1 6 (sum) C2_Q14_A1

C2_Q14_A2 302 6 1.963576 1 6 (sum) C2_Q14_A2

C2_Q14_A3 302 6 1.824503 1 6 (sum) C2_Q14_A3

C2_Q14_A4 302 7 1.748344 0 6 (sum) C2_Q14_A4

C2_Q14_A5 302 6 1.589404 1 6 (sum) C2_Q14_A5

C2_Q14_A6 302 6 1.569536 1 6 (sum) C2_Q14_A6

C2_Q14_A7 302 6 1.470199 1 6 (sum) C2_Q14_A7

C2_Q14_A8 302 7 1.483444 0 6 (sum) C2_Q14_A8

C2_Q14_A9 302 6 1.443709 1 6 (sum) C2_Q14_A9

C2_Q14_A10 302 6 1.519868 1 6 (sum) C2_Q14_A10

C2_Q15_B1 302 6 1.317881 1 6 (sum) C2_Q15_B1

C2_Q15_B2 302 5 1.298013 1 5 (sum) C2_Q15_B2

C2_Q15_B3 302 6 1.31457 0 5 (sum) C2_Q15_B3

C2_Q15_B4 302 6 1.344371 0 5 (sum) C2_Q15_B4

C2_Q15_B5 302 6 1.384106 0 5 (sum) C2_Q15_B5

C2_Q15_B6 302 7 1.466887 0 6 (sum) C2_Q15_B6

C2_Q15_B7 302 6 1.5 1 6 (sum) C2_Q15_B7

C2_Q15_A1 302 6 1.629139 1 6 (sum) C2_Q15_A1

C2_Q15_A2 302 6 1.516556 1 6 (sum) C2_Q15_A2

C2_Q15_A3 302 5 1.466887 1 5 (sum) C2_Q15_A3

C2_Q15_A4 302 6 1.433775 0 5 (sum) C2_Q15_A4

C2_Q15_A5 302 5 1.331126 1 6 (sum) C2_Q15_A5

C2_Q15_A6 302 5 1.288079 1 5 (sum) C2_Q15_A6

C2_Q15_A7 302 5 1.301325 1 5 (sum) C2_Q15_A7

C2_Q15_A8 302 7 1.245033 0 6 (sum) C2_Q15_A8

C2_Q15_A9 302 5 1.264901 1 5 (sum) C2_Q15_A9

C2_Q15_A10 302 5 1.231788 1 5 (sum) C2_Q15_A10

C2_Q16_B1 302 6 1.397351 1 6 (sum) C2_Q16_B1

C2_Q16_B2 302 6 1.486755 1 6 (sum) C2_Q16_B2

C2_Q16_B3 302 7 1.466887 0 6 (sum) C2_Q16_B3

C2_Q16_B4 302 6 1.503311 0 5 (sum) C2_Q16_B4

C2_Q16_B5 302 6 1.506623 0 5 (sum) C2_Q16_B5

C2_Q16_B6 302 7 1.533113 0 6 (sum) C2_Q16_B6

C2_Q16_B7 302 6 1.619205 1 6 (sum) C2_Q16_B7

C2_Q16_A1 302 6 1.728477 1 6 (sum) C2_Q16_A1

C2_Q16_A2 302 5 1.596026 1 5 (sum) C2_Q16_A2

C2_Q16_A3 302 6 1.619205 1 6 (sum) C2_Q16_A3

C2_Q16_A4 302 7 1.523179 0 6 (sum) C2_Q16_A4

C2_Q16_A5 302 5 1.403974 1 5 (sum) C2_Q16_A5

C2_Q16_A6 302 5 1.407285 1 5 (sum) C2_Q16_A6

C2_Q16_A7 302 6 1.317881 1 6 (sum) C2_Q16_A7

C2_Q16_A8 302 6 1.34106 0 5 (sum) C2_Q16_A8

C2_Q16_A9 302 6 1.354305 1 6 (sum) C2_Q16_A9

C2_Q16_A10 302 5 1.34106 1 5 (sum) C2_Q16_A10

C2_Q17_B1 302 6 1.347682 1 6 (sum) C2_Q17_B1

C2_Q17_B2 302 6 1.387417 1 6 (sum) C2_Q17_B2

C2_Q17_B3 302 6 1.397351 0 5 (sum) C2_Q17_B3

C2_Q17_B4 302 7 1.423841 0 6 (sum) C2_Q17_B4

C2_Q17_B5 302 6 1.42053 0 5 (sum) C2_Q17_B5

C2_Q17_B6 302 7 1.506623 0 6 (sum) C2_Q17_B6

C2_Q17_B7 302 6 1.592715 1 6 (sum) C2_Q17_B7

C2_Q17_A1 302 6 1.721854 1 6 (sum) C2_Q17_A1

C2_Q17_A2 302 6 1.619205 1 6 (sum) C2_Q17_A2

C2_Q17_A3 302 6 1.602649 1 6 (sum) C2_Q17_A3

C2_Q17_A4 302 7 1.407285 0 6 (sum) C2_Q17_A4

C2_Q17_A5 302 5 1.334437 1 6 (sum) C2_Q17_A5

C2_Q17_A6 302 6 1.31457 1 6 (sum) C2_Q17_A6

C2_Q17_A7 302 5 1.291391 1 6 (sum) C2_Q17_A7

C2_Q17_A8 302 6 1.238411 0 5 (sum) C2_Q17_A8

C2_Q17_A9 302 5 1.248344 1 5 (sum) C2_Q17_A9

C2_Q17_A10 302 5 1.258278 1 5 (sum) C2_Q17_A10

C2_Q18_B1 302 5 1.377483 1 5 (sum) C2_Q18_B1

C2_Q18_B2 302 5 1.44702 1 5 (sum) C2_Q18_B2

C2_Q18_B3 302 7 1.569536 0 6 (sum) C2_Q18_B3

C2_Q18_B4 302 7 1.592715 0 6 (sum) C2_Q18_B4

C2_Q18_B5 302 7 1.708609 0 6 (sum) C2_Q18_B5

C2_Q18_B6 302 7 1.731788 0 6 (sum) C2_Q18_B6

C2_Q18_B7 302 6 1.768212 1 6 (sum) C2_Q18_B7

C2_Q18_A1 302 6 1.81457 1 6 (sum) C2_Q18_A1

C2_Q18_A2 302 6 1.615894 1 6 (sum) C2_Q18_A2

C2_Q18_A3 302 6 1.430464 1 6 (sum) C2_Q18_A3

C2_Q18_A4 302 5 1.301325 0 4 (sum) C2_Q18_A4

C2_Q18_A5 302 4 1.225166 1 4 (sum) C2_Q18_A5

C2_Q18_A6 302 5 1.168874 1 5 (sum) C2_Q18_A6

C2_Q18_A7 302 3 1.109272 1 3 (sum) C2_Q18_A7

C2_Q18_A8 302 4 1.082781 0 3 (sum) C2_Q18_A8

C2_Q18_A9 302 4 1.096026 1 4 (sum) C2_Q18_A9

C2_Q18_A10 302 3 1.056291 1 3 (sum) C2_Q18_A10

C2_Q19_B1 302 5 1.321192 1 5 (sum) C2_Q19_B1

C2_Q19_B2 302 5 1.397351 1 5 (sum) C2_Q19_B2

C2_Q19_B3 302 7 1.476821 0 6 (sum) C2_Q19_B3

C2_Q19_B4 302 7 1.546358 0 6 (sum) C2_Q19_B4

C2_Q19_B5 302 6 1.629139 0 5 (sum) C2_Q19_B5

C2_Q19_B6 302 6 1.668874 0 5 (sum) C2_Q19_B6

C2_Q19_B7 302 5 1.665563 1 5 (sum) C2_Q19_B7

C2_Q19_A1 302 6 1.864238 1 6 (sum) C2_Q19_A1

C2_Q19_A2 302 6 1.63245 1 6 (sum) C2_Q19_A2

C2_Q19_A3 302 6 1.417219 1 6 (sum) C2_Q19_A3

C2_Q19_A4 302 5 1.284768 0 4 (sum) C2_Q19_A4

C2_Q19_A5 302 4 1.228477 1 4 (sum) C2_Q19_A5

C2_Q19_A6 302 4 1.129139 1 4 (sum) C2_Q19_A6

C2_Q19_A7 302 4 1.122517 1 4 (sum) C2_Q19_A7

C2_Q19_A8 302 5 1.092715 0 4 (sum) C2_Q19_A8

C2_Q19_A9 302 3 1.092715 1 3 (sum) C2_Q19_A9

C2_Q19_A10 302 3 1.059603 1 3 (sum) C2_Q19_A10

C2_Q21_B1 302 6 1.407285 1 6 (sum) C2_Q20_B1

C2_Q21_B2 302 5 1.39404 1 5 (sum) C2_Q20_B2

C2_Q21_B3 302 6 1.437086 0 5 (sum) C2_Q20_B3

C2_Q21_B4 302 6 1.486755 0 5 (sum) C2_Q20_B4

C2_Q21_B5 302 6 1.486755 0 5 (sum) C2_Q20_B5

C2_Q21_B6 302 7 1.566225 0 6 (sum) C2_Q20_B6

C2_Q21_B7 302 5 1.589404 1 5 (sum) C2_Q20_B7

C2_Q21_A1 302 6 1.844371 1 6 (sum) C2_Q20_A1

C2_Q21_A2 302 6 1.715232 1 6 (sum) C2_Q20_A2

C2_Q21_A3 302 6 1.602649 1 6 (sum) C2_Q20_A3

C2_Q21_A4 302 6 1.433775 0 5 (sum) C2_Q20_A4

C2_Q21_A5 302 4 1.360927 1 4 (sum) C2_Q20_A5

C2_Q21_A6 302 5 1.377483 1 5 (sum) C2_Q20_A6

C2_Q21_A7 302 6 1.31457 1 6 (sum) C2_Q20_A7

C2_Q21_A8 302 6 1.331126 0 5 (sum) C2_Q20_A8

C2_Q21_A9 302 6 1.294702 1 6 (sum) C2_Q20_A9

C2_Q21_A10 302 5 1.298013 1 5 (sum) C2_Q20_A10

C2_Q20_B1 302 6 1.36755 1 6 (sum) C2_Q21_B1

C2_Q20_B2 302 5 1.437086 1 5 (sum) C2_Q21_B2

C2_Q20_B3 302 6 1.397351 0 5 (sum) C2_Q21_B3

C2_Q20_B4 302 6 1.493377 0 5 (sum) C2_Q21_B4

C2_Q20_B5 302 6 1.5 0 5 (sum) C2_Q21_B5

C2_Q20_B6 302 7 1.486755 0 6 (sum) C2_Q21_B6

C2_Q20_B7 302 6 1.665563 1 6 (sum) C2_Q21_B7

C2_Q20_A1 302 6 2.13245 1 6 (sum) C2_Q21_A1

C2_Q20_A2 302 6 1.927152 1 6 (sum) C2_Q21_A2

C2_Q20_A3 302 6 1.672185 1 6 (sum) C2_Q21_A3

C2_Q20_A4 302 6 1.503311 0 5 (sum) C2_Q21_A4

C2_Q20_A5 302 5 1.39404 1 5 (sum) C2_Q21_A5

C2_Q20_A6 302 5 1.347682 1 5 (sum) C2_Q21_A6

C2_Q20_A7 302 6 1.208609 1 6 (sum) C2_Q21_A7

C2_Q20_A8 302 6 1.218543 0 5 (sum) C2_Q21_A8

C2_Q20_A9 302 5 1.208609 1 6 (sum) C2_Q21_A9

C2_Q20_A10 302 5 1.228477 1 5 (sum) C2_Q21_A10

C2_Q22_B1 302 5 1.470199 1 5 (sum) C2_Q22_B1

C2_Q22_B2 302 6 1.490066 1 6 (sum) C2_Q22_B2

C2_Q22_B3 302 6 1.493377 0 5 (sum) C2_Q22_B3

C2_Q22_B4 302 6 1.529801 0 5 (sum) C2_Q22_B4

C2_Q22_B5 302 6 1.55298 0 5 (sum) C2_Q22_B5

C2_Q22_B6 302 7 1.665563 0 6 (sum) C2_Q22_B6

C2_Q22_B7 302 5 1.798013 1 5 (sum) C2_Q22_B7

C2_Q22_A1 302 6 2.009934 1 6 (sum) C2_Q22_A1

C2_Q22_A2 302 6 1.943709 1 6 (sum) C2_Q22_A2

C2_Q22_A3 302 5 1.781457 1 5 (sum) C2_Q22_A3

C2_Q22_A4 302 6 1.60596 0 5 (sum) C2_Q22_A4

C2_Q22_A5 302 6 1.52649 1 6 (sum) C2_Q22_A5

C2_Q22_A6 302 6 1.470199 1 6 (sum) C2_Q22_A6

C2_Q22_A7 302 6 1.397351 1 6 (sum) C2_Q22_A7

C2_Q22_A8 302 6 1.344371 0 5 (sum) C2_Q22_A8

C2_Q22_A9 302 6 1.347682 1 6 (sum) C2_Q22_A9

C2_Q22_A10 302 5 1.370861 1 5 (sum) C2_Q22_A10

C2_Q23_B1 302 5 1.31457 1 5 (sum) C2_Q23_B1

C2_Q23_B2 302 6 1.331126 1 6 (sum) C2_Q23_B2

C2_Q23_B3 302 6 1.390728 0 5 (sum) C2_Q23_B3

C2_Q23_B4 302 6 1.443709 0 5 (sum) C2_Q23_B4

C2_Q23_B5 302 6 1.42053 0 5 (sum) C2_Q23_B5

C2_Q23_B6 302 7 1.543046 0 6 (sum) C2_Q23_B6

C2_Q23_B7 302 5 1.655629 1 5 (sum) C2_Q23_B7

C2_Q23_A1 302 6 1.930464 1 6 (sum) C2_Q23_A1

C2_Q23_A2 302 5 1.761589 1 5 (sum) C2_Q23_A2

C2_Q23_A3 302 5 1.622517 1 5 (sum) C2_Q23_A3

C2_Q23_A4 302 6 1.496689 0 5 (sum) C2_Q23_A4

C2_Q23_A5 302 5 1.417219 1 5 (sum) C2_Q23_A5

C2_Q23_A6 302 5 1.36755 1 5 (sum) C2_Q23_A6

C2_Q23_A7 302 6 1.298013 1 6 (sum) C2_Q23_A7

C2_Q23_A8 302 5 1.221854 0 4 (sum) C2_Q23_A8

C2_Q23_A9 302 6 1.288079 1 6 (sum) C2_Q23_A9

C2_Q23_A10 302 5 1.274834 1 5 (sum) C2_Q23_A10

C2_Q24_B1 302 5 1.298013 1 5 (sum) C2_Q24_B1

C2_Q24_B2 302 6 1.301325 1 6 (sum) C2_Q24_B2

C2_Q24_B3 302 7 1.321192 0 6 (sum) C2_Q24_B3

C2_Q24_B4 302 7 1.413907 0 6 (sum) C2_Q24_B4

C2_Q24_B5 302 6 1.384106 0 5 (sum) C2_Q24_B5

C2_Q24_B6 302 7 1.470199 0 6 (sum) C2_Q24_B6

C2_Q24_B7 302 5 1.539735 1 5 (sum) C2_Q24_B7

C2_Q24_A1 302 6 1.678808 1 6 (sum) C2_Q24_A1

C2_Q24_A2 302 5 1.536424 1 5 (sum) C2_Q24_A2

C2_Q24_A3 302 5 1.503311 1 5 (sum) C2_Q24_A3

C2_Q24_A4 302 5 1.370861 0 4 (sum) C2_Q24_A4

C2_Q24_A5 302 5 1.331126 1 6 (sum) C2_Q24_A5

C2_Q24_A6 302 5 1.271523 1 5 (sum) C2_Q24_A6

C2_Q24_A7 302 5 1.241722 1 5 (sum) C2_Q24_A7

C2_Q24_A8 302 5 1.182119 0 4 (sum) C2_Q24_A8

C2_Q24_A9 302 4 1.192053 1 4 (sum) C2_Q24_A9

C2_Q24_A10 302 4 1.205298 1 4 (sum) C2_Q24_A10

C2_Q25_B1 302 2 .0860927 0 26 (sum) C2_Q25_B1

C2_Q25_B2 302 2 .0298013 0 9 (sum) C2_Q25_B2

C2_Q25_B3 302 1 0 0 0 (sum) C2_Q25_B3

C2_Q25_B4 302 1 0 0 0 (sum) C2_Q25_B4

C2_Q25_B5 302 1 0 0 0 (sum) C2_Q25_B5

C2_Q25_B6 302 1 0 0 0 (sum) C2_Q25_B6

C2_Q25_B7 302 2 .013245 0 2 (sum) C2_Q25_B7

C2_Q25_A1 302 71 16.10166 0 125 (sum) C2_Q25_A1

C2_Q25_A2 302 87 24.29636 0 225 (sum) C2_Q25_A2

C2_Q25_A3 302 67 17.67053 0 130 (sum) C2_Q25_A3

C2_Q25_A4 302 60 9.766556 0 125 (sum) C2_Q25_A4

C2_Q25_A5 302 34 3.950662 0 55 (sum) C2_Q25_A5

C2_Q25_A6 302 27 1.354636 0 40 (sum) C2_Q25_A6

C2_Q25_A7 302 11 .2698675 0 13 (sum) C2_Q25_A7

C2_Q25_A8 302 5 .0678808 0 8 (sum) C2_Q25_A8

C2_Q25_A9 302 3 .0099338 0 2 (sum) C2_Q25_A9

C2_Q25_A10 302 2 3.307947 0 999 (sum) C2_Q25_A10

C3_Q1_B1 302 6 .1059603 0 5 (sum) C3_Q1_B1

C3_Q1_B2 302 6 .102649 0 5 (sum) C3_Q1_B2

C3_Q1_B3 302 6 .1125828 0 5 (sum) C3_Q1_B3

C3_Q1_B4 302 6 .1291391 0 5 (sum) C3_Q1_B4

C3_Q1_B5 302 6 .1192053 0 5 (sum) C3_Q1_B5

C3_Q1_B6 302 5 .1225166 0 5 (sum) C3_Q1_B6

C3_Q1_B7 302 7 .1291391 0 6 (sum) C3_Q1_B7

C3_Q1_A1 302 5 .1059603 0 6 (sum) C3_Q1_A1

C3_Q1_A2 302 6 .1059603 0 5 (sum) C3_Q1_A2

C3_Q1_A3 302 5 .1059603 0 5 (sum) C3_Q1_A3

C3_Q1_A4 302 5 .0860927 0 5 (sum) C3_Q1_A4

C3_Q1_A5 302 4 .0728477 0 4 (sum) C3_Q1_A5

C3_Q1_A6 302 5 .0860927 0 5 (sum) C3_Q1_A6

C3_Q1_A7 302 4 .0860927 0 5 (sum) C3_Q1_A7

C3_Q1_A8 302 5 .0860927 0 5 (sum) C3_Q1_A8

C3_Q1_A9 302 4 .0629139 0 3 (sum) C3_Q1_A9

C3_Q1_A10 302 4 .0728477 0 4 (sum) C3_Q1_A10

C3_Q2_B1 302 5 .089404 0 4 (sum) C3_Q2_B1

C3_Q2_B2 302 5 .089404 0 4 (sum) C3_Q2_B2

C3_Q2_B3 302 6 .1092715 0 5 (sum) C3_Q2_B3

C3_Q2_B4 302 6 .102649 0 5 (sum) C3_Q2_B4

C3_Q2_B5 302 6 .0927152 0 5 (sum) C3_Q2_B5

C3_Q2_B6 302 6 .0927152 0 5 (sum) C3_Q2_B6

C3_Q2_B7 302 6 .1125828 0 5 (sum) C3_Q2_B7

C3_Q2_A1 302 5 .102649 0 5 (sum) C3_Q2_A1

C3_Q2_A2 302 5 .0993377 0 4 (sum) C3_Q2_A2

C3_Q2_A3 302 6 .0993377 0 5 (sum) C3_Q2_A3

C3_Q2_A4 302 5 .0860927 0 5 (sum) C3_Q2_A4

C3_Q2_A5 302 4 .0662252 0 3 (sum) C3_Q2_A5

C3_Q2_A6 302 4 .0695364 0 4 (sum) C3_Q2_A6

C3_Q2_A7 302 4 .0695364 0 5 (sum) C3_Q2_A7

C3_Q2_A8 302 4 .0695364 0 4 (sum) C3_Q2_A8

C3_Q2_A9 302 2 .0529801 0 1 (sum) C3_Q2_A9

C3_Q2_A10 302 4 .0695364 0 4 (sum) C3_Q2_A10

C3_Q3_B1 302 6 .1092715 0 5 (sum) C3_Q3_B1

C3_Q3_B2 302 6 .1192053 0 5 (sum) C3_Q3_B2

C3_Q3_B3 302 6 .1225166 0 5 (sum) C3_Q3_B3

C3_Q3_B4 302 7 .1225166 0 6 (sum) C3_Q3_B4

C3_Q3_B5 302 5 .1125828 0 5 (sum) C3_Q3_B5

C3_Q3_B6 302 7 .1225166 0 6 (sum) C3_Q3_B6

C3_Q3_B7 302 6 .115894 0 6 (sum) C3_Q3_B7

C3_Q3_A1 302 6 .1092715 0 6 (sum) C3_Q3_A1

C3_Q3_A2 302 5 .1225166 0 5 (sum) C3_Q3_A2

C3_Q3_A3 302 6 .1092715 0 5 (sum) C3_Q3_A3

C3_Q3_A4 302 6 .0960265 0 5 (sum) C3_Q3_A4

C3_Q3_A5 302 5 .0827815 0 5 (sum) C3_Q3_A5

C3_Q3_A6 302 6 .089404 0 5 (sum) C3_Q3_A6

C3_Q3_A7 302 4 .0860927 0 5 (sum) C3_Q3_A7

C3_Q3_A8 302 6 .0927152 0 5 (sum) C3_Q3_A8

C3_Q3_A9 302 4 .0728477 0 5 (sum) C3_Q3_A9

C3_Q3_A10 302 4 .0794702 0 4 (sum) C3_Q3_A10

C3_Q4_B1 302 6 .102649 0 5 (sum) C3_Q4_B1

C3_Q4_B2 302 6 .1192053 0 5 (sum) C3_Q4_B2

C3_Q4_B3 302 6 .115894 0 5 (sum) C3_Q4_B3

C3_Q4_B4 302 7 .1225166 0 6 (sum) C3_Q4_B4

C3_Q4_B5 302 6 .1125828 0 6 (sum) C3_Q4_B5

C3_Q4_B6 302 5 .1059603 0 4 (sum) C3_Q4_B6

C3_Q4_B7 302 7 .1291391 0 6 (sum) C3_Q4_B7

C3_Q4_A1 302 6 .1225166 0 6 (sum) C3_Q4_A1

C3_Q4_A2 302 5 .102649 0 4 (sum) C3_Q4_A2

C3_Q4_A3 302 6 .1092715 0 5 (sum) C3_Q4_A3

C3_Q4_A4 302 5 .0827815 0 5 (sum) C3_Q4_A4

C3_Q4_A5 302 4 .0695364 0 4 (sum) C3_Q4_A5

C3_Q4_A6 302 5 .0794702 0 4 (sum) C3_Q4_A6

C3_Q4_A7 302 6 .0860927 0 5 (sum) C3_Q4_A7

C3_Q4_A8 302 4 .0827815 0 4 (sum) C3_Q4_A8

C3_Q4_A9 302 5 .0794702 0 4 (sum) C3_Q4_A9

C3_Q4_A10 302 4 .0728477 0 4 (sum) C3_Q4_A10

C3_Q5_B1 302 5 .0960265 0 5 (sum) C3_Q5_B1

C3_Q5_B2 302 6 .1125828 0 5 (sum) C3_Q5_B2

C3_Q5_B3 302 6 .115894 0 5 (sum) C3_Q5_B3

C3_Q5_B4 302 7 .1225166 0 6 (sum) C3_Q5_B4

C3_Q5_B5 302 6 .1092715 0 6 (sum) C3_Q5_B5

C3_Q5_B6 302 7 .1225166 0 6 (sum) C3_Q5_B6

C3_Q5_B7 302 6 .1192053 0 5 (sum) C3_Q5_B7

C3_Q5_A1 302 5 .1125828 0 5 (sum) C3_Q5_A1

C3_Q5_A2 302 6 .1059603 0 5 (sum) C3_Q5_A2

C3_Q5_A3 302 6 .0993377 0 5 (sum) C3_Q5_A3

C3_Q5_A4 302 5 .0927152 0 5 (sum) C3_Q5_A4

C3_Q5_A5 302 4 .0695364 0 4 (sum) C3_Q5_A5

C3_Q5_A6 302 4 .0728477 0 4 (sum) C3_Q5_A6

C3_Q5_A7 302 4 .0761589 0 5 (sum) C3_Q5_A7

C3_Q5_A8 302 5 .0827815 0 4 (sum) C3_Q5_A8

C3_Q5_A9 302 4 .0662252 0 3 (sum) C3_Q5_A9

C3_Q5_A10 302 5 .0827815 0 4 (sum) C3_Q5_A10

C3_Q6_B1 302 5 .0960265 0 5 (sum) C3_Q6_B1

C3_Q6_B2 302 6 .1092715 0 5 (sum) C3_Q6_B2

C3_Q6_B3 302 6 .1125828 0 5 (sum) C3_Q6_B3

C3_Q6_B4 302 7 .1225166 0 6 (sum) C3_Q6_B4

C3_Q6_B5 302 6 .1092715 0 6 (sum) C3_Q6_B5

C3_Q6_B6 302 7 .1192053 0 6 (sum) C3_Q6_B6

C3_Q6_B7 302 7 .1125828 0 6 (sum) C3_Q6_B7

C3_Q6_A1 302 6 .115894 0 6 (sum) C3_Q6_A1

C3_Q6_A2 302 4 .0960265 0 4 (sum) C3_Q6_A2

C3_Q6_A3 302 6 .0993377 0 5 (sum) C3_Q6_A3

C3_Q6_A4 302 4 .0827815 0 5 (sum) C3_Q6_A4

C3_Q6_A5 302 4 .0662252 0 3 (sum) C3_Q6_A5

C3_Q6_A6 302 4 .0695364 0 4 (sum) C3_Q6_A6

C3_Q6_A7 302 4 .0662252 0 4 (sum) C3_Q6_A7

C3_Q6_A8 302 4 .0728477 0 4 (sum) C3_Q6_A8

C3_Q6_A9 302 4 .0662252 0 3 (sum) C3_Q6_A9

C3_Q6_A10 302 4 .0695364 0 4 (sum) C3_Q6_A10

C3_Q7_B1 302 5 .102649 0 5 (sum) C3_Q7_B1

C3_Q7_B2 302 6 .1092715 0 5 (sum) C3_Q7_B2

C3_Q7_B3 302 6 .1125828 0 5 (sum) C3_Q7_B3

C3_Q7_B4 302 5 .1059603 0 5 (sum) C3_Q7_B4

C3_Q7_B5 302 6 .1092715 0 6 (sum) C3_Q7_B5

C3_Q7_B6 302 7 .1192053 0 6 (sum) C3_Q7_B6

C3_Q7_B7 302 4 .1225166 0 6 (sum) C3_Q7_B7

C3_Q7_A1 302 6 .115894 0 6 (sum) C3_Q7_A1

C3_Q7_A2 302 5 .0960265 0 4 (sum) C3_Q7_A2

C3_Q7_A3 302 5 .0927152 0 4 (sum) C3_Q7_A3

C3_Q7_A4 302 4 .0827815 0 5 (sum) C3_Q7_A4

C3_Q7_A5 302 5 .0761589 0 4 (sum) C3_Q7_A5

C3_Q7_A6 302 4 .0695364 0 4 (sum) C3_Q7_A6

C3_Q7_A7 302 3 .0662252 0 3 (sum) C3_Q7_A7

C3_Q7_A8 302 4 .0662252 0 3 (sum) C3_Q7_A8

C3_Q7_A9 302 4 .0629139 0 3 (sum) C3_Q7_A9

C3_Q7_A10 302 4 .0695364 0 3 (sum) C3_Q7_A10

C3_Q8_B1 302 5 .0860927 0 4 (sum) C3_Q8_B1

C3_Q8_B2 302 6 .1059603 0 5 (sum) C3_Q8_B2

C3_Q8_B3 302 5 .1059603 0 4 (sum) C3_Q8_B3

C3_Q8_B4 302 5 .0993377 0 4 (sum) C3_Q8_B4

C3_Q8_B5 302 5 .0960265 0 4 (sum) C3_Q8_B5

C3_Q8_B6 302 5 .102649 0 4 (sum) C3_Q8_B6

C3_Q8_B7 302 5 .1092715 0 4 (sum) C3_Q8_B7

C3_Q8_A1 302 5 .1059603 0 5 (sum) C3_Q8_A1

C3_Q8_A2 302 5 .0993377 0 4 (sum) C3_Q8_A2

C3_Q8_A3 302 5 .102649 0 4 (sum) C3_Q8_A3

C3_Q8_A4 302 4 .0662252 0 3 (sum) C3_Q8_A4

C3_Q8_A5 302 3 .0695364 0 3 (sum) C3_Q8_A5

C3_Q8_A6 302 4 .0794702 0 5 (sum) C3_Q8_A6

C3_Q8_A7 302 3 .0662252 0 3 (sum) C3_Q8_A7

C3_Q8_A8 302 4 .0728477 0 3 (sum) C3_Q8_A8

C3_Q8_A9 302 3 .0662252 0 3 (sum) C3_Q8_A9

C3_Q8_A10 302 4 .0761589 0 3 (sum) C3_Q8_A10

C3_Q9_B1 302 4 .1059603 0 4 (sum) C3_Q9_B1

C3_Q9_B2 302 7 .115894 0 6 (sum) C3_Q9_B2

C3_Q9_B3 302 6 .1092715 0 5 (sum) C3_Q9_B3

C3_Q9_B4 302 5 .115894 0 5 (sum) C3_Q9_B4

C3_Q9_B5 302 5 .1291391 0 5 (sum) C3_Q9_B5

C3_Q9_B6 302 7 .1225166 0 6 (sum) C3_Q9_B6

C3_Q9_B7 302 6 .1225166 0 6 (sum) C3_Q9_B7

C3_Q9_A1 302 6 .1192053 0 5 (sum) C3_Q9_A1

C3_Q9_A2 302 6 .1125828 0 5 (sum) C3_Q9_A2

C3_Q9_A3 302 6 .1059603 0 5 (sum) C3_Q9_A3

C3_Q9_A4 302 5 .0927152 0 5 (sum) C3_Q9_A4

C3_Q9_A5 302 5 .0761589 0 4 (sum) C3_Q9_A5

C3_Q9_A6 302 6 .089404 0 5 (sum) C3_Q9_A6

C3_Q9_A7 302 5 .0827815 0 5 (sum) C3_Q9_A7

C3_Q9_A8 302 4 .0794702 0 3 (sum) C3_Q9_A8

C3_Q9_A9 302 4 .0695364 0 5 (sum) C3_Q9_A9

C3_Q9_A10 302 4 .0662252 0 3 (sum) C3_Q9_A10

C3_Q10_B1 302 5 .1092715 0 4 (sum) C3_Q10_B1

C3_Q10_B2 302 6 .1225166 0 6 (sum) C3_Q10_B2

C3_Q10_B3 302 6 .1125828 0 5 (sum) C3_Q10_B3

C3_Q10_B4 302 6 .115894 0 5 (sum) C3_Q10_B4

C3_Q10_B5 302 7 .1258278 0 6 (sum) C3_Q10_B5

C3_Q10_B6 302 7 .1125828 0 6 (sum) C3_Q10_B6

C3_Q10_B7 302 5 .1357616 0 6 (sum) C3_Q10_B7

C3_Q10_A1 302 7 .1125828 0 6 (sum) C3_Q10_A1

C3_Q10_A2 302 6 .1324503 0 5 (sum) C3_Q10_A2

C3_Q10_A3 302 6 .1225166 0 5 (sum) C3_Q10_A3

C3_Q10_A4 302 4 .089404 0 3 (sum) C3_Q10_A4

C3_Q10_A5 302 5 .089404 0 4 (sum) C3_Q10_A5

C3_Q10_A6 302 5 .0860927 0 4 (sum) C3_Q10_A6

C3_Q10_A7 302 5 .0927152 0 5 (sum) C3_Q10_A7

C3_Q10_A8 302 5 .089404 0 4 (sum) C3_Q10_A8

C3_Q10_A9 302 5 .089404 0 5 (sum) C3_Q10_A9

C3_Q10_A10 302 5 .0993377 0 4 (sum) C3_Q10_A10

C3_Q11_B1 302 6 .1092715 0 5 (sum) C3_Q11_B1

C3_Q11_B2 302 6 .1357616 0 6 (sum) C3_Q11_B2

C3_Q11_B3 302 6 .1192053 0 6 (sum) C3_Q11_B3

C3_Q11_B4 302 7 .1225166 0 6 (sum) C3_Q11_B4

C3_Q11_B5 302 6 .1225166 0 5 (sum) C3_Q11_B5

C3_Q11_B6 302 7 .1258278 0 6 (sum) C3_Q11_B6

C3_Q11_B7 302 7 .1291391 0 6 (sum) C3_Q11_B7

C3_Q11_A1 302 5 .1291391 0 6 (sum) C3_Q11_A1

C3_Q11_A2 302 7 .1291391 0 6 (sum) C3_Q11_A2

C3_Q11_A3 302 6 .1225166 0 6 (sum) C3_Q11_A3

C3_Q11_A4 302 5 .0960265 0 4 (sum) C3_Q11_A4

C3_Q11_A5 302 6 .089404 0 5 (sum) C3_Q11_A5

C3_Q11_A6 302 6 .0927152 0 5 (sum) C3_Q11_A6

C3_Q11_A7 302 6 .089404 0 6 (sum) C3_Q11_A7

C3_Q11_A8 302 6 .0927152 0 5 (sum) C3_Q11_A8

C3_Q11_A9 302 5 .0860927 0 5 (sum) C3_Q11_A9

C3_Q11_A10 302 6 .089404 0 5 (sum) C3_Q11_A10

C3_Q12_B1 302 4 .0728477 0 5 (sum) C3_Q12_B1

C3_Q12_B2 302 6 .1092715 0 6 (sum) C3_Q12_B2

C3_Q12_B3 302 5 .102649 0 6 (sum) C3_Q12_B3

C3_Q12_B4 302 6 .0960265 0 6 (sum) C3_Q12_B4

C3_Q12_B5 302 6 .0993377 0 6 (sum) C3_Q12_B5

C3_Q12_B6 302 5 .1059603 0 6 (sum) C3_Q12_B6

C3_Q12_B7 302 5 .0927152 0 5 (sum) C3_Q12_B7

C3_Q12_A1 302 5 .1059603 0 5 (sum) C3_Q12_A1

C3_Q12_A2 302 6 .1059603 0 5 (sum) C3_Q12_A2

C3_Q12_A3 302 6 .0993377 0 5 (sum) C3_Q12_A3

C3_Q12_A4 302 5 .0827815 0 5 (sum) C3_Q12_A4

C3_Q12_A5 302 4 .0761589 0 5 (sum) C3_Q12_A5

C3_Q12_A6 302 5 .0794702 0 5 (sum) C3_Q12_A6

C3_Q12_A7 302 4 .0728477 0 5 (sum) C3_Q12_A7

C3_Q12_A8 302 4 .0761589 0 5 (sum) C3_Q12_A8

C3_Q12_A9 302 4 .0728477 0 5 (sum) C3_Q12_A9

C3_Q12_A10 302 4 .0761589 0 5 (sum) C3_Q12_A10

C3_Q13_B1 302 5 .0761589 0 5 (sum) C3_Q13_B1

C3_Q13_B2 302 6 .0927152 0 6 (sum) C3_Q13_B2

C3_Q13_B3 302 6 .0993377 0 6 (sum) C3_Q13_B3

C3_Q13_B4 302 6 .102649 0 6 (sum) C3_Q13_B4

C3_Q13_B5 302 6 .1059603 0 6 (sum) C3_Q13_B5

C3_Q13_B6 302 7 .1059603 0 6 (sum) C3_Q13_B6

C3_Q13_B7 302 5 .102649 0 5 (sum) C3_Q13_B7

C3_Q13_A1 302 6 .1059603 0 6 (sum) C3_Q13_A1

C3_Q13_A2 302 5 .089404 0 4 (sum) C3_Q13_A2

C3_Q13_A3 302 6 .102649 0 5 (sum) C3_Q13_A3

C3_Q13_A4 302 5 .0927152 0 5 (sum) C3_Q13_A4

C3_Q13_A5 302 4 .0761589 0 5 (sum) C3_Q13_A5

C3_Q13_A6 302 5 .0794702 0 5 (sum) C3_Q13_A6

C3_Q13_A7 302 5 .0761589 0 5 (sum) C3_Q13_A7

C3_Q13_A8 302 4 .0761589 0 5 (sum) C3_Q13_A8

C3_Q13_A9 302 4 .0728477 0 5 (sum) C3_Q13_A9

C3_Q13_A10 302 5 .0794702 0 5 (sum) C3_Q13_A10

C3_Q14_B1 302 7 .115894 0 6 (sum) C3_Q14_B1

C3_Q14_B2 302 6 .1291391 0 6 (sum) C3_Q14_B2

C3_Q14_B3 302 7 .1225166 0 6 (sum) C3_Q14_B3

C3_Q14_B4 302 6 .1291391 0 6 (sum) C3_Q14_B4

C3_Q14_B5 302 5 .1291391 0 6 (sum) C3_Q14_B5

C3_Q14_B6 302 7 .1291391 0 6 (sum) C3_Q14_B6

C3_Q14_B7 302 7 .1258278 0 6 (sum) C3_Q14_B7

C3_Q14_A1 302 6 .1092715 0 6 (sum) C3_Q14_A1

C3_Q14_A2 302 6 .1192053 0 6 (sum) C3_Q14_A2

C3_Q14_A3 302 6 .1324503 0 6 (sum) C3_Q14_A3

C3_Q14_A4 302 6 .1092715 0 5 (sum) C3_Q14_A4

C3_Q14_A5 302 4 .089404 0 5 (sum) C3_Q14_A5

C3_Q14_A6 302 5 .089404 0 6 (sum) C3_Q14_A6

C3_Q14_A7 302 5 .0993377 0 6 (sum) C3_Q14_A7

C3_Q14_A8 302 5 .089404 0 6 (sum) C3_Q14_A8

C3_Q14_A9 302 5 .0761589 0 5 (sum) C3_Q14_A9

C3_Q14_A10 302 5 .089404 0 5 (sum) C3_Q14_A10

C3_Q15_B1 302 6 .0993377 0 5 (sum) C3_Q15_B1

C3_Q15_B2 302 6 .0993377 0 5 (sum) C3_Q15_B2

C3_Q15_B3 302 6 .1059603 0 5 (sum) C3_Q15_B3

C3_Q15_B4 302 5 .102649 0 4 (sum) C3_Q15_B4

C3_Q15_B5 302 5 .0960265 0 4 (sum) C3_Q15_B5

C3_Q15_B6 302 5 .0960265 0 5 (sum) C3_Q15_B6

C3_Q15_B7 302 4 .0993377 0 4 (sum) C3_Q15_B7

C3_Q15_A1 302 6 .0993377 0 5 (sum) C3_Q15_A1

C3_Q15_A2 302 5 .0960265 0 4 (sum) C3_Q15_A2

C3_Q15_A3 302 5 .1059603 0 4 (sum) C3_Q15_A3

C3_Q15_A4 302 5 .0794702 0 4 (sum) C3_Q15_A4

C3_Q15_A5 302 4 .0794702 0 4 (sum) C3_Q15_A5

C3_Q15_A6 302 5 .0794702 0 4 (sum) C3_Q15_A6

C3_Q15_A7 302 4 .0761589 0 3 (sum) C3_Q15_A7

C3_Q15_A8 302 4 .0761589 0 3 (sum) C3_Q15_A8

C3_Q15_A9 302 4 .0695364 0 3 (sum) C3_Q15_A9

C3_Q15_A10 302 4 .0794702 0 4 (sum) C3_Q15_A10

C3_Q16_B1 302 5 .0993377 0 5 (sum) C3_Q16_B1

C3_Q16_B2 302 5 .1092715 0 5 (sum) C3_Q16_B2

C3_Q16_B3 302 5 .0993377 0 4 (sum) C3_Q16_B3

C3_Q16_B4 302 6 .1092715 0 5 (sum) C3_Q16_B4

C3_Q16_B5 302 6 .1059603 0 5 (sum) C3_Q16_B5

C3_Q16_B6 302 7 .1092715 0 6 (sum) C3_Q16_B6

C3_Q16_B7 302 6 .1092715 0 5 (sum) C3_Q16_B7

C3_Q16_A1 302 6 .1092715 0 6 (sum) C3_Q16_A1

C3_Q16_A2 302 5 .0993377 0 4 (sum) C3_Q16_A2

C3_Q16_A3 302 5 .102649 0 4 (sum) C3_Q16_A3

C3_Q16_A4 302 4 .089404 0 4 (sum) C3_Q16_A4

C3_Q16_A5 302 5 .0761589 0 4 (sum) C3_Q16_A5

C3_Q16_A6 302 4 .0794702 0 4 (sum) C3_Q16_A6

C3_Q16_A7 302 4 .0794702 0 5 (sum) C3_Q16_A7

C3_Q16_A8 302 5 .0860927 0 6 (sum) C3_Q16_A8

C3_Q16_A9 302 4 .0629139 0 3 (sum) C3_Q16_A9

C3_Q16_A10 302 5 .0761589 0 4 (sum) C3_Q16_A10

C3_Q17_B1 302 6 .1059603 0 5 (sum) C3_Q17_B1

C3_Q17_B2 302 6 .1092715 0 5 (sum) C3_Q17_B2

C3_Q17_B3 302 6 .0993377 0 5 (sum) C3_Q17_B3

C3_Q17_B4 302 6 .102649 0 5 (sum) C3_Q17_B4

C3_Q17_B5 302 6 .0960265 0 5 (sum) C3_Q17_B5

C3_Q17_B6 302 7 .1125828 0 6 (sum) C3_Q17_B6

C3_Q17_B7 302 6 .1125828 0 5 (sum) C3_Q17_B7

C3_Q17_A1 302 5 .1059603 0 5 (sum) C3_Q17_A1

C3_Q17_A2 302 5 .0960265 0 4 (sum) C3_Q17_A2

C3_Q17_A3 302 5 .1092715 0 4 (sum) C3_Q17_A3

C3_Q17_A4 302 6 .0993377 0 5 (sum) C3_Q17_A4

C3_Q17_A5 302 5 .0794702 0 4 (sum) C3_Q17_A5

C3_Q17_A6 302 4 .0794702 0 4 (sum) C3_Q17_A6

C3_Q17_A7 302 4 .0761589 0 5 (sum) C3_Q17_A7

C3_Q17_A8 302 4 .0794702 0 4 (sum) C3_Q17_A8

C3_Q17_A9 302 4 .0728477 0 3 (sum) C3_Q17_A9

C3_Q17_A10 302 5 .0794702 0 4 (sum) C3_Q17_A10

C3_Q18_B1 302 5 .0794702 0 5 (sum) C3_Q18_B1

C3_Q18_B2 302 6 .089404 0 5 (sum) C3_Q18_B2

C3_Q18_B3 302 5 .0927152 0 5 (sum) C3_Q18_B3

C3_Q18_B4 302 5 .102649 0 5 (sum) C3_Q18_B4

C3_Q18_B5 302 5 .089404 0 6 (sum) C3_Q18_B5

C3_Q18_B6 302 5 .0993377 0 6 (sum) C3_Q18_B6

C3_Q18_B7 302 7 .1092715 0 6 (sum) C3_Q18_B7

C3_Q18_A1 302 5 .0927152 0 5 (sum) C3_Q18_A1

C3_Q18_A2 302 5 .0860927 0 4 (sum) C3_Q18_A2

C3_Q18_A3 302 5 .0794702 0 4 (sum) C3_Q18_A3

C3_Q18_A4 302 4 .0728477 0 4 (sum) C3_Q18_A4

C3_Q18_A5 302 3 .0662252 0 4 (sum) C3_Q18_A5

C3_Q18_A6 302 3 .0596026 0 2 (sum) C3_Q18_A6

C3_Q18_A7 302 3 .0596026 0 3 (sum) C3_Q18_A7

C3_Q18_A8 302 3 .0629139 0 3 (sum) C3_Q18_A8

C3_Q18_A9 302 3 .0596026 0 3 (sum) C3_Q18_A9

C3_Q18_A10 302 3 .0629139 0 3 (sum) C3_Q18_A10

C3_Q19_B1 302 5 .0827815 0 5 (sum) C3_Q19_B1

C3_Q19_B2 302 6 .0960265 0 5 (sum) C3_Q19_B2

C3_Q19_B3 302 5 .089404 0 5 (sum) C3_Q19_B3

C3_Q19_B4 302 5 .0993377 0 5 (sum) C3_Q19_B4

C3_Q19_B5 302 5 .089404 0 6 (sum) C3_Q19_B5

C3_Q19_B6 302 6 .1092715 0 6 (sum) C3_Q19_B6

C3_Q19_B7 302 6 .1192053 0 6 (sum) C3_Q19_B7

C3_Q19_A1 302 6 .1125828 0 5 (sum) C3_Q19_A1

C3_Q19_A2 302 6 .0993377 0 5 (sum) C3_Q19_A2

C3_Q19_A3 302 4 .0827815 0 4 (sum) C3_Q19_A3

C3_Q19_A4 302 5 .0761589 0 4 (sum) C3_Q19_A4

C3_Q19_A5 302 3 .0629139 0 3 (sum) C3_Q19_A5

C3_Q19_A6 302 4 .0662252 0 3 (sum) C3_Q19_A6

C3_Q19_A7 302 3 .0662252 0 3 (sum) C3_Q19_A7

C3_Q19_A8 302 3 .0761589 0 4 (sum) C3_Q19_A8

C3_Q19_A9 302 4 .0629139 0 3 (sum) C3_Q19_A9

C3_Q19_A10 302 4 .0662252 0 3 (sum) C3_Q19_A10

C3_Q21_B1 302 6 .0993377 0 5 (sum) C3_Q20_B1

C3_Q21_B2 302 6 .1092715 0 5 (sum) C3_Q20_B2

C3_Q21_B3 302 6 .0993377 0 5 (sum) C3_Q20_B3

C3_Q21_B4 302 5 .1092715 0 5 (sum) C3_Q20_B4

C3_Q21_B5 302 5 .102649 0 5 (sum) C3_Q20_B5

C3_Q21_B6 302 7 .115894 0 6 (sum) C3_Q20_B6

C3_Q21_B7 302 6 .1192053 0 6 (sum) C3_Q20_B7

C3_Q21_A1 302 6 .1225166 0 6 (sum) C3_Q20_A1

C3_Q21_A2 302 5 .0993377 0 4 (sum) C3_Q20_A2

C3_Q21_A3 302 5 .1092715 0 4 (sum) C3_Q20_A3

C3_Q21_A4 302 5 .0960265 0 4 (sum) C3_Q20_A4

C3_Q21_A5 302 5 .0794702 0 4 (sum) C3_Q20_A5

C3_Q21_A6 302 4 .0827815 0 3 (sum) C3_Q20_A6

C3_Q21_A7 302 5 .0827815 0 4 (sum) C3_Q20_A7

C3_Q21_A8 302 5 .089404 0 4 (sum) C3_Q20_A8

C3_Q21_A9 302 4 .0728477 0 3 (sum) C3_Q20_A9

C3_Q21_A10 302 5 .0860927 0 5 (sum) C3_Q20_A10

C3_Q20_B1 302 5 .0728477 0 4 (sum) C3_Q21_B1

C3_Q20_B2 302 5 .102649 0 5 (sum) C3_Q21_B2

C3_Q20_B3 302 6 .0960265 0 5 (sum) C3_Q21_B3

C3_Q20_B4 302 6 .102649 0 5 (sum) C3_Q21_B4

C3_Q20_B5 302 6 .102649 0 5 (sum) C3_Q21_B5

C3_Q20_B6 302 6 .0927152 0 6 (sum) C3_Q21_B6

C3_Q20_B7 302 7 .1059603 0 6 (sum) C3_Q21_B7

C3_Q20_A1 302 7 .1059603 0 6 (sum) C3_Q21_A1

C3_Q20_A2 302 4 .0860927 0 3 (sum) C3_Q21_A2

C3_Q20_A3 302 5 .0960265 0 4 (sum) C3_Q21_A3

C3_Q20_A4 302 4 .0860927 0 4 (sum) C3_Q21_A4

C3_Q20_A5 302 4 .0695364 0 4 (sum) C3_Q21_A5

C3_Q20_A6 302 4 .0728477 0 4 (sum) C3_Q21_A6

C3_Q20_A7 302 3 .0662252 0 3 (sum) C3_Q21_A7

C3_Q20_A8 302 5 .0761589 0 4 (sum) C3_Q21_A8

C3_Q20_A9 302 5 .0728477 0 4 (sum) C3_Q21_A9

C3_Q20_A10 302 5 .0761589 0 4 (sum) C3_Q21_A10

C3_Q22_B1 302 6 .1092715 0 5 (sum) C3_Q22_B1

C3_Q22_B2 302 6 .1192053 0 5 (sum) C3_Q22_B2

C3_Q22_B3 302 6 .1192053 0 5 (sum) C3_Q22_B3

C3_Q22_B4 302 5 .115894 0 5 (sum) C3_Q22_B4

C3_Q22_B5 302 6 .1192053 0 6 (sum) C3_Q22_B5

C3_Q22_B6 302 5 .1125828 0 5 (sum) C3_Q22_B6

C3_Q22_B7 302 6 .1258278 0 6 (sum) C3_Q22_B7

C3_Q22_A1 302 6 .1125828 0 6 (sum) C3_Q22_A1

C3_Q22_A2 302 6 .0993377 0 5 (sum) C3_Q22_A2

C3_Q22_A3 302 6 .1059603 0 5 (sum) C3_Q22_A3

C3_Q22_A4 302 5 .089404 0 5 (sum) C3_Q22_A4

C3_Q22_A5 302 4 .0794702 0 5 (sum) C3_Q22_A5

C3_Q22_A6 302 6 .0927152 0 6 (sum) C3_Q22_A6

C3_Q22_A7 302 4 .0827815 0 6 (sum) C3_Q22_A7

C3_Q22_A8 302 4 .0827815 0 4 (sum) C3_Q22_A8

C3_Q22_A9 302 4 .0728477 0 5 (sum) C3_Q22_A9

C3_Q22_A10 302 5 .0860927 0 5 (sum) C3_Q22_A10

C3_Q23_B1 302 4 .0960265 0 4 (sum) C3_Q23_B1

C3_Q23_B2 302 5 .102649 0 4 (sum) C3_Q23_B2

C3_Q23_B3 302 6 .1059603 0 5 (sum) C3_Q23_B3

C3_Q23_B4 302 4 .1092715 0 4 (sum) C3_Q23_B4

C3_Q23_B5 302 6 .1125828 0 5 (sum) C3_Q23_B5

C3_Q23_B6 302 6 .1092715 0 5 (sum) C3_Q23_B6

C3_Q23_B7 302 7 .1258278 0 6 (sum) C3_Q23_B7

C3_Q23_A1 302 5 .1059603 0 5 (sum) C3_Q23_A1

C3_Q23_A2 302 5 .0960265 0 5 (sum) C3_Q23_A2

C3_Q23_A3 302 6 .1092715 0 5 (sum) C3_Q23_A3

C3_Q23_A4 302 4 .0761589 0 3 (sum) C3_Q23_A4

C3_Q23_A5 302 4 .0695364 0 4 (sum) C3_Q23_A5

C3_Q23_A6 302 5 .0827815 0 5 (sum) C3_Q23_A6

C3_Q23_A7 302 4 .0827815 0 6 (sum) C3_Q23_A7

C3_Q23_A8 302 4 .0827815 0 4 (sum) C3_Q23_A8

C3_Q23_A9 302 4 .0728477 0 5 (sum) C3_Q23_A9

C3_Q23_A10 302 5 .0860927 0 5 (sum) C3_Q23_A10

C3_Q24_B1 302 4 .0993377 0 4 (sum) C3_Q24_B1

C3_Q24_B2 302 4 .0960265 0 4 (sum) C3_Q24_B2

C3_Q24_B3 302 6 .1125828 0 5 (sum) C3_Q24_B3

C3_Q24_B4 302 5 .1059603 0 4 (sum) C3_Q24_B4

C3_Q24_B5 302 6 .1059603 0 5 (sum) C3_Q24_B5

C3_Q24_B6 302 5 .1059603 0 5 (sum) C3_Q24_B6

C3_Q24_B7 302 6 .1125828 0 6 (sum) C3_Q24_B7

C3_Q24_A1 302 5 .1059603 0 5 (sum) C3_Q24_A1

C3_Q24_A2 302 4 .089404 0 3 (sum) C3_Q24_A2

C3_Q24_A3 302 5 .0993377 0 4 (sum) C3_Q24_A3

C3_Q24_A4 302 3 .0695364 0 3 (sum) C3_Q24_A4

C3_Q24_A5 302 4 .0695364 0 4 (sum) C3_Q24_A5

C3_Q24_A6 302 4 .0695364 0 4 (sum) C3_Q24_A6

C3_Q24_A7 302 4 .0695364 0 4 (sum) C3_Q24_A7

C3_Q24_A8 302 4 .0695364 0 4 (sum) C3_Q24_A8

C3_Q24_A9 302 3 .0596026 0 3 (sum) C3_Q24_A9

C3_Q24_A10 302 5 .0761589 0 4 (sum) C3_Q24_A10

C3_Q25_B1 302 1 0 0 0 (sum) C3_Q25_B1

C3_Q25_B2 302 1 0 0 0 (sum) C3_Q25_B2

C3_Q25_B3 302 1 0 0 0 (sum) C3_Q25_B3

C3_Q25_B4 302 1 0 0 0 (sum) C3_Q25_B4

C3_Q25_B5 302 1 0 0 0 (sum) C3_Q25_B5

C3_Q25_B6 302 1 0 0 0 (sum) C3_Q25_B6

C3_Q25_B7 302 1 0 0 0 (sum) C3_Q25_B7

C3_Q25_A1 302 13 1.175497 0 50 (sum) C3_Q25_A1

C3_Q25_A2 302 16 1.627483 0 75 (sum) C3_Q25_A2

C3_Q25_A3 302 13 .9205298 0 55 (sum) C3_Q25_A3

C3_Q25_A4 302 12 .410596 0 30 (sum) C3_Q25_A4

C3_Q25_A5 302 9 .160596 0 15 (sum) C3_Q25_A5

C3_Q25_A6 302 6 .0413907 0 5 (sum) C3_Q25_A6

C3_Q25_A7 302 2 .0049669 0 1.5 (sum) C3_Q25_A7

C3_Q25_A8 302 2 .0016556 0 .5 (sum) C3_Q25_A8

C3_Q25_A9 302 2 .0016556 0 .5 (sum) C3_Q25_A9

C3_Q25_A10 302 1 0 0 0 (sum) C3_Q25_A10

namsinh 302 284 13366.83 5541 14974 Year of Birth

dantoc 302 2 .0397351 0 1 Ethnic group

nganhhoc 302 8 2.556291 1 8 Medical specialty

b1 302 4 2.129139 1 4 PSST - item 1: Anger/irritability

b2 302 4 2.023179 1 4 PSST - item 2: Anxiety/tension

b3 302 4 1.662252 1 4 PSST - item 3: Tearfulness

b4 302 4 1.774834 1 4 PSST - item 4: Depressed mood

b5 302 4 2.178808 1 4 PSST - item 5: Decrease interest in work

b6 302 4 2.115894 1 4 PSST - item 6: Decrease interest in home

b7 302 4 2.135762 1 4 PSST - item 7: Decrease interest in social activities

b8 302 4 2.145695 1 4 PSST - item 8: Difficulty concentrating

b9 302 4 2.278146 1 4 PSST - item 9: Fatigue/lack of energy

b10 302 4 1.784768 1 4 PSST - item 10: Overeating/food craving

b11 302 4 1.55298 1 4 PSST - item 11: Insomnia

b12 302 4 2.009934 1 4 PSST - item 12: Hypersomnia

b13 302 4 1.721854 1 4 PSST - item 13: Feeling overwhelmed

b14 302 4 2.460265 1 4 PSST - item 14: Physical symptoms

b15 302 4 2.145695 1 4 PSST - item 15: School/work efficiency or productivity

b16 302 4 1.784768 1 4 PSST - item 16: Relationships with friends, classmates/co-workers

b17 302 3 1.569536 1 3 PSST - item 17: Relationship with your family

b18 302 4 1.850993 1 4 PSST - item 18: Social life activities

b19 302 4 1.513245 1 4 PSST - item 19: Home responsibilities

c1 302 8 13.56623 10 17 Age of menarche (Years)

c3 302 8 5.043046 3 53 Menstrual days (Days)

c4 302 19 29.87748 0 45 Cycle days (Days)

c6 302 2 .0695364 0 1 Had more than one pregnancy

d1 302 5 2.900662 1 5 ABO blood type

d2 302 3 1.182119 1 3 Rh blood type

d7 302 2 .0198675 0 1 Psychological disorders in 1st degree relatives

e1 302 155 48.40099 35.5 70.6 Weight

e2 302 50 155.805 136.5 171 High

e20 302 1 0 0 0 Smoking

f1 302 2 1.245033 1 2 IPAQ: Vigorous physical activities during last 7 days

f1a 74 7 3.581081 1 7 IPAQ: Vigorous physical activities days

f2a 46 6 1.195652 0 7 IPAQ: Vigorous physical activities hours

f2b 51 11 26.17647 0 90 IPAQ: Vigorous physical activities minutes

f3a 134 7 4.037313 1 7 IPAQ: Moderate physical activities days

f4a 70 9 1.5 0 10 IPAQ: Moderate physical activities hours

f4b 76 12 26.25 0 90 IPAQ: Moderate physical activities minutes

f5a 241 7 4.614108 1 7 IPAQ: Walking physical activities days

f6a 99 8 1.242424 0 30 IPAQ: Walking physical activities hours

f6b 166 14 22.40361 0 60 IPAQ: Walking physical activities minutes

f7a 258 20 8.085271 1 96 IPAQ: sitting hours

f7b 66 7 8.80303 0 96 IPAQ: sitting minutes

h1 301 4 1.92691 1 4 PHQ-9 - item 1: Little interest or pleasure in doing things?

h2 301 4 1.551495 1 4 PHQ-9 - item 2: Feeling down, depressed, or hopeless?

h3 301 4 2.016611 1 4 PHQ-9 - item 3: Trouble falling or staying asleep, or sleeping too much?

h4 301 4 2.016611 1 4 PHQ-9 - item 4: Feeling tired or having little energy?

h5 301 4 1.767442 1 4 PHQ-9 - item 5: Poor appetite or overeating?

h6 301 4 1.647841 1 4 PHQ-9 - item 6: Feeling bad about yourself - or that you are a failure or have l

h7 301 4 1.730897 1 4 PHQ-9 - item 7: Trouble concentrating on things, such as reading the newspaper o

h8 301 4 1.269103 1 4 PHQ-9 - item 8: Moving or speaking so slowly that other people could have notice

h9 301 3 1.053156 1 3 PHQ-9 - item 9: Thoughts that you would be better off dead, or of hurting yourse

PSST 302 3 .3046358 0 2 PSST diagnosis

BMI 302 291 19.94214 14.25161 29.57656 BMI (kg/m2)

level_BMI 302 4 1.827815 1 4 BMI classification

IPAQ 302 3 1.682119 1 3 Physical Activity in the last 7 days

level_PHQ9 302 3 1.778146 1 3 Depression level based on PHQ-9

C7 302 2 .0264901 0 1 History of C-section

C8 302 2 .0629139 0 1 History of Term births

C9 302 2 .0099338 0 1 History of Preterm births

C10 302 2 .013245 0 1 History of Abortions

E9 302 3 1.933775 1 3 Alcohol consumption in the last 12 months

E15 302 3 1.566225 1 3 Caffein consumption in the last 12 months

b1_2 276 4 2.213768 1 4 Post-PSST - item 1: Anger/irritability

b2_2 276 4 2.119565 1 4 Post-PSST - item 2: Anxiety/tension

b3_2 276 4 1.775362 1 4 Post-PSST - item 3: Tearfulness

b4_2 276 4 1.844203 1 4 Post-PSST - item 4: Depressed mood

b5_2 276 4 2.184783 1 4 Post-PSST - item 5: Decrease interest in work

b6_2 276 4 2.097826 1 4 Post-PSST - item 6: Decrease interest in home

b7_2 276 4 2.032609 1 4 Post-PSST - item 7: Decrease interest in social activities

b8_2 276 4 2.155797 1 4 Post-PSST - item 8: Difficulty concentrating

b9_2 276 4 2.26087 1 4 Post-PSST - item 9: Fatigue/lack of energy

b10_2 276 4 1.717391 1 4 Post-PSST - item 10: Overeating/food craving

b11_2 276 4 1.550725 1 4 Post-PSST - item 11: Insomnia

b12_2 276 4 1.985507 1 4 Post-PSST - item 12: Hypersomnia

b13_2 276 4 1.782609 1 4 Post-PSST - item 13: Feeling overwhelmed

b14_2 276 4 2.300725 1 4 Post-PSST - item 14: Physical symptoms

b15_2 276 4 2.050725 1 4 Post-PSST - item 15: School/work efficiency or productivity

b16_2 276 4 1.673913 1 4 Post-PSST - item 16: Relationships with friends, classmates/co-workers

b17_2 276 4 1.521739 1 4 Post-PSST - item 17: Relationship with your family

b18_2 276 4 1.764493 1 4 Post-PSST - item 18: Social life activities

b19_2 276 4 1.449275 1 4 Post-PSST - item 19: Home responsibilities

PSST_2 276 3 .2971014 0 2 PSST 2 diagnosis

CPASS_final 302 3 .1291391 0 2 CPASS diagnosis

---------------------------------------------------------------------------------------------------------------------------------------------------------------------------------------------------------------------------------------------------------------

. codebook

---------------------------------------------------------------------------------------------------------------------------------------------------------------------------------------------------------------------------------------------------------------

maso Participant ID

---------------------------------------------------------------------------------------------------------------------------------------------------------------------------------------------------------------------------------------------------------------

type: numeric (double)

range: [101,1153] units: 1

unique values: 302 missing .: 0/302

mean: 600.195

std. dev: 339.212

percentiles: 10% 25% 50% 75% 90%

132 308 606.5 913 1113

---------------------------------------------------------------------------------------------------------------------------------------------------------------------------------------------------------------------------------------------------------------

C1_Q1_B1 (sum) C1_Q1_B1

---------------------------------------------------------------------------------------------------------------------------------------------------------------------------------------------------------------------------------------------------------------

type: numeric (double)

label: CPASS1, but 1 nonmissing value is not labeled

range: [0,6] units: 1

unique values: 7 missing .: 0/302

tabulation: Freq. Numeric Label

1 0

199 1 not at all

50 2 minimal

26 3 mild

18 4 moderate

7 5 severe

1 6 extreme

---------------------------------------------------------------------------------------------------------------------------------------------------------------------------------------------------------------------------------------------------------------

C1_Q1_B2 (sum) C1_Q1_B2

---------------------------------------------------------------------------------------------------------------------------------------------------------------------------------------------------------------------------------------------------------------

type: numeric (double)

label: CPASS1, but 1 nonmissing value is not labeled

range: [0,6] units: 1

unique values: 7 missing .: 0/302

tabulation: Freq. Numeric Label

1 0

200 1 not at all

46 2 minimal

28 3 mild

18 4 moderate

6 5 severe

3 6 extreme

---------------------------------------------------------------------------------------------------------------------------------------------------------------------------------------------------------------------------------------------------------------

C1_Q1_B3 (sum) C1_Q1_B3

---------------------------------------------------------------------------------------------------------------------------------------------------------------------------------------------------------------------------------------------------------------

type: numeric (double)

label: CPASS1

range: [1,6] units: 1

unique values: 6 missing .: 0/302

tabulation: Freq. Numeric Label

197 1 not at all

49 2 minimal

30 3 mild

14 4 moderate

11 5 severe

1 6 extreme

---------------------------------------------------------------------------------------------------------------------------------------------------------------------------------------------------------------------------------------------------------------

C1_Q1_B4 (sum) C1_Q1_B4

---------------------------------------------------------------------------------------------------------------------------------------------------------------------------------------------------------------------------------------------------------------

type: numeric (double)

label: CPASS1

range: [1,6] units: 1

unique values: 6 missing .: 0/302

tabulation: Freq. Numeric Label

201 1 not at all

46 2 minimal

31 3 mild

17 4 moderate

5 5 severe

2 6 extreme

---------------------------------------------------------------------------------------------------------------------------------------------------------------------------------------------------------------------------------------------------------------

C1_Q1_B5 (sum) C1_Q1_B5

---------------------------------------------------------------------------------------------------------------------------------------------------------------------------------------------------------------------------------------------------------------

type: numeric (double)

label: CPASS1, but 1 nonmissing value is not labeled

range: [0,6] units: 1

unique values: 7 missing .: 0/302

tabulation: Freq. Numeric Label

1 0

198 1 not at all

50 2 minimal

35 3 mild

14 4 moderate

3 5 severe

1 6 extreme

---------------------------------------------------------------------------------------------------------------------------------------------------------------------------------------------------------------------------------------------------------------

C1_Q1_B6 (sum) C1_Q1_B6

---------------------------------------------------------------------------------------------------------------------------------------------------------------------------------------------------------------------------------------------------------------

type: numeric (double)

label: CPASS1

range: [1,6] units: 1

unique values: 6 missing .: 0/302

tabulation: Freq. Numeric Label

196 1 not at all

50 2 minimal

31 3 mild

16 4 moderate

6 5 severe

3 6 extreme

---------------------------------------------------------------------------------------------------------------------------------------------------------------------------------------------------------------------------------------------------------------

C1_Q1_B7 (sum) C1_Q1_B7

---------------------------------------------------------------------------------------------------------------------------------------------------------------------------------------------------------------------------------------------------------------

type: numeric (double)

label: CPASS1, but 1 nonmissing value is not labeled

range: [0,6] units: 1

unique values: 7 missing .: 0/302

tabulation: Freq. Numeric Label

1 0

192 1 not at all

52 2 minimal

35 3 mild

15 4 moderate

5 5 severe

2 6 extreme

---------------------------------------------------------------------------------------------------------------------------------------------------------------------------------------------------------------------------------------------------------------

C1_Q1_A1 (sum) C1_Q1_A1

---------------------------------------------------------------------------------------------------------------------------------------------------------------------------------------------------------------------------------------------------------------

type: numeric (double)

label: CPASS1

range: [1,6] units: 1

unique values: 6 missing .: 0/302

tabulation: Freq. Numeric Label

169 1 not at all

65 2 minimal

41 3 mild

16 4 moderate

9 5 severe

2 6 extreme

---------------------------------------------------------------------------------------------------------------------------------------------------------------------------------------------------------------------------------------------------------------

C1_Q1_A2 (sum) C1_Q1_A2

---------------------------------------------------------------------------------------------------------------------------------------------------------------------------------------------------------------------------------------------------------------

type: numeric (double)

label: CPASS1

range: [1,6] units: 1

unique values: 6 missing .: 0/302

tabulation: Freq. Numeric Label

166 1 not at all

65 2 minimal

38 3 mild

21 4 moderate

7 5 severe

5 6 extreme

---------------------------------------------------------------------------------------------------------------------------------------------------------------------------------------------------------------------------------------------------------------

C1_Q1_A3 (sum) C1_Q1_A3

---------------------------------------------------------------------------------------------------------------------------------------------------------------------------------------------------------------------------------------------------------------

type: numeric (double)

label: CPASS1

range: [1,6] units: 1

unique values: 6 missing .: 0/302

tabulation: Freq. Numeric Label

183 1 not at all

56 2 minimal

34 3 mild

21 4 moderate

4 5 severe

4 6 extreme

---------------------------------------------------------------------------------------------------------------------------------------------------------------------------------------------------------------------------------------------------------------

C1_Q1_A4 (sum) C1_Q1_A4

---------------------------------------------------------------------------------------------------------------------------------------------------------------------------------------------------------------------------------------------------------------

type: numeric (double)

label: CPASS1

range: [1,6] units: 1

unique values: 6 missing .: 0/302

tabulation: Freq. Numeric Label

200 1 not at all

56 2 minimal

27 3 mild

12 4 moderate

5 5 severe

2 6 extreme

---------------------------------------------------------------------------------------------------------------------------------------------------------------------------------------------------------------------------------------------------------------

C1_Q1_A5 (sum) C1_Q1_A5

---------------------------------------------------------------------------------------------------------------------------------------------------------------------------------------------------------------------------------------------------------------

type: numeric (double)

label: CPASS1

range: [1,6] units: 1

unique values: 6 missing .: 0/302

tabulation: Freq. Numeric Label

215 1 not at all

52 2 minimal

19 3 mild

14 4 moderate

1 5 severe

1 6 extreme

---------------------------------------------------------------------------------------------------------------------------------------------------------------------------------------------------------------------------------------------------------------

C1_Q1_A6 (sum) C1_Q1_A6

---------------------------------------------------------------------------------------------------------------------------------------------------------------------------------------------------------------------------------------------------------------

type: numeric (double)

label: CPASS1

range: [1,6] units: 1

unique values: 6 missing .: 0/302

tabulation: Freq. Numeric Label

226 1 not at all

46 2 minimal

23 3 mild

5 4 moderate

1 5 severe

1 6 extreme

---------------------------------------------------------------------------------------------------------------------------------------------------------------------------------------------------------------------------------------------------------------

C1_Q1_A7 (sum) C1_Q1_A7

---------------------------------------------------------------------------------------------------------------------------------------------------------------------------------------------------------------------------------------------------------------

type: numeric (double)

label: CPASS1

range: [1,5] units: 1

unique values: 5 missing .: 0/302

tabulation: Freq. Numeric Label

238 1 not at all

37 2 minimal

17 3 mild

8 4 moderate

2 5 severe

---------------------------------------------------------------------------------------------------------------------------------------------------------------------------------------------------------------------------------------------------------------

C1_Q1_A8 (sum) C1_Q1_A8

---------------------------------------------------------------------------------------------------------------------------------------------------------------------------------------------------------------------------------------------------------------

type: numeric (double)

label: CPASS1

range: [1,5] units: 1

unique values: 5 missing .: 0/302

tabulation: Freq. Numeric Label

239 1 not at all

39 2 minimal

13 3 mild

8 4 moderate

3 5 severe

---------------------------------------------------------------------------------------------------------------------------------------------------------------------------------------------------------------------------------------------------------------

C1_Q1_A9 (sum) C1_Q1_A9

---------------------------------------------------------------------------------------------------------------------------------------------------------------------------------------------------------------------------------------------------------------

type: numeric (double)

label: CPASS1

range: [1,6] units: 1

unique values: 6 missing .: 0/302

tabulation: Freq. Numeric Label

245 1 not at all

35 2 minimal

12 3 mild

8 4 moderate

1 5 severe

1 6 extreme

---------------------------------------------------------------------------------------------------------------------------------------------------------------------------------------------------------------------------------------------------------------

C1_Q1_A10 (sum) C1_Q1_A10

---------------------------------------------------------------------------------------------------------------------------------------------------------------------------------------------------------------------------------------------------------------

type: numeric (double)

label: CPASS1

range: [1,6] units: 1

unique values: 6 missing .: 0/302

tabulation: Freq. Numeric Label

234 1 not at all

40 2 minimal

21 3 mild

5 4 moderate

1 5 severe

1 6 extreme

---------------------------------------------------------------------------------------------------------------------------------------------------------------------------------------------------------------------------------------------------------------

C1_Q2_B1 (sum) C1_Q2_B1

---------------------------------------------------------------------------------------------------------------------------------------------------------------------------------------------------------------------------------------------------------------

type: numeric (double)

label: CPASS1, but 1 nonmissing value is not labeled

range: [0,5] units: 1

unique values: 6 missing .: 0/302

tabulation: Freq. Numeric Label

1 0

243 1 not at all

24 2 minimal

19 3 mild

11 4 moderate

4 5 severe

---------------------------------------------------------------------------------------------------------------------------------------------------------------------------------------------------------------------------------------------------------------

C1_Q2_B2 (sum) C1_Q2_B2

---------------------------------------------------------------------------------------------------------------------------------------------------------------------------------------------------------------------------------------------------------------

type: numeric (double)

label: CPASS1, but 1 nonmissing value is not labeled

range: [0,6] units: 1

unique values: 7 missing .: 0/302

tabulation: Freq. Numeric Label

1 0

239 1 not at all

31 2 minimal

17 3 mild

9 4 moderate

4 5 severe

1 6 extreme

---------------------------------------------------------------------------------------------------------------------------------------------------------------------------------------------------------------------------------------------------------------

C1_Q2_B3 (sum) C1_Q2_B3

---------------------------------------------------------------------------------------------------------------------------------------------------------------------------------------------------------------------------------------------------------------

type: numeric (double)

label: CPASS1

range: [1,6] units: 1

unique values: 6 missing .: 0/302

tabulation: Freq. Numeric Label

250 1 not at all

21 2 minimal

14 3 mild

13 4 moderate

3 5 severe

1 6 extreme

---------------------------------------------------------------------------------------------------------------------------------------------------------------------------------------------------------------------------------------------------------------

C1_Q2_B4 (sum) C1_Q2_B4

---------------------------------------------------------------------------------------------------------------------------------------------------------------------------------------------------------------------------------------------------------------

type: numeric (double)

label: CPASS1

range: [1,6] units: 1

unique values: 6 missing .: 0/302

tabulation: Freq. Numeric Label

240 1 not at all

27 2 minimal

20 3 mild

7 4 moderate

7 5 severe

1 6 extreme

---------------------------------------------------------------------------------------------------------------------------------------------------------------------------------------------------------------------------------------------------------------

C1_Q2_B5 (sum) C1_Q2_B5

---------------------------------------------------------------------------------------------------------------------------------------------------------------------------------------------------------------------------------------------------------------

type: numeric (double)

label: CPASS1, but 1 nonmissing value is not labeled

range: [0,6] units: 1

unique values: 7 missing .: 0/302

tabulation: Freq. Numeric Label

1 0

236 1 not at all

28 2 minimal

29 3 mild

5 4 moderate

2 5 severe

1 6 extreme

---------------------------------------------------------------------------------------------------------------------------------------------------------------------------------------------------------------------------------------------------------------

C1_Q2_B6 (sum) C1_Q2_B6

---------------------------------------------------------------------------------------------------------------------------------------------------------------------------------------------------------------------------------------------------------------

type: numeric (double)

label: CPASS1

range: [1,6] units: 1

unique values: 6 missing .: 0/302

tabulation: Freq. Numeric Label

239 1 not at all

31 2 minimal

20 3 mild

6 4 moderate

5 5 severe

1 6 extreme

---------------------------------------------------------------------------------------------------------------------------------------------------------------------------------------------------------------------------------------------------------------

C1_Q2_B7 (sum) C1_Q2_B7

---------------------------------------------------------------------------------------------------------------------------------------------------------------------------------------------------------------------------------------------------------------

type: numeric (double)

label: CPASS1, but 1 nonmissing value is not labeled

range: [0,6] units: 1

unique values: 7 missing .: 0/302

tabulation: Freq. Numeric Label

1 0

232 1 not at all

29 2 minimal

30 3 mild

6 4 moderate

1 5 severe

3 6 extreme

---------------------------------------------------------------------------------------------------------------------------------------------------------------------------------------------------------------------------------------------------------------

C1_Q2_A1 (sum) C1_Q2_A1

---------------------------------------------------------------------------------------------------------------------------------------------------------------------------------------------------------------------------------------------------------------

type: numeric (double)

label: CPASS1

range: [1,6] units: 1

unique values: 6 missing .: 0/302

tabulation: Freq. Numeric Label

230 1 not at all

37 2 minimal

19 3 mild

9 4 moderate

5 5 severe

2 6 extreme

---------------------------------------------------------------------------------------------------------------------------------------------------------------------------------------------------------------------------------------------------------------

C1_Q2_A2 (sum) C1_Q2_A2

---------------------------------------------------------------------------------------------------------------------------------------------------------------------------------------------------------------------------------------------------------------

type: numeric (double)

label: CPASS1

range: [1,6] units: 1

unique values: 6 missing .: 0/302

tabulation: Freq. Numeric Label

226 1 not at all

29 2 minimal

27 3 mild

13 4 moderate

4 5 severe

3 6 extreme

---------------------------------------------------------------------------------------------------------------------------------------------------------------------------------------------------------------------------------------------------------------

C1_Q2_A3 (sum) C1_Q2_A3

---------------------------------------------------------------------------------------------------------------------------------------------------------------------------------------------------------------------------------------------------------------

type: numeric (double)

label: CPASS1

range: [1,6] units: 1

unique values: 6 missing .: 0/302

tabulation: Freq. Numeric Label

223 1 not at all

35 2 minimal

29 3 mild

8 4 moderate

4 5 severe

3 6 extreme

---------------------------------------------------------------------------------------------------------------------------------------------------------------------------------------------------------------------------------------------------------------

C1_Q2_A4 (sum) C1_Q2_A4

---------------------------------------------------------------------------------------------------------------------------------------------------------------------------------------------------------------------------------------------------------------

type: numeric (double)

label: CPASS1

range: [1,6] units: 1

unique values: 6 missing .: 0/302

tabulation: Freq. Numeric Label

243 1 not at all

30 2 minimal

16 3 mild

9 4 moderate

2 5 severe

2 6 extreme

---------------------------------------------------------------------------------------------------------------------------------------------------------------------------------------------------------------------------------------------------------------

C1_Q2_A5 (sum) C1_Q2_A5

---------------------------------------------------------------------------------------------------------------------------------------------------------------------------------------------------------------------------------------------------------------

type: numeric (double)

label: CPASS1

range: [1,6] units: 1

unique values: 6 missing .: 0/302

tabulation: Freq. Numeric Label

245 1 not at all

31 2 minimal

17 3 mild

7 4 moderate

1 5 severe

1 6 extreme

---------------------------------------------------------------------------------------------------------------------------------------------------------------------------------------------------------------------------------------------------------------

C1_Q2_A6 (sum) C1_Q2_A6

---------------------------------------------------------------------------------------------------------------------------------------------------------------------------------------------------------------------------------------------------------------

type: numeric (double)

label: CPASS1

range: [1,6] units: 1

unique values: 6 missing .: 0/302

tabulation: Freq. Numeric Label

252 1 not at all

29 2 minimal

14 3 mild

5 4 moderate

1 5 severe

1 6 extreme

---------------------------------------------------------------------------------------------------------------------------------------------------------------------------------------------------------------------------------------------------------------

C1_Q2_A7 (sum) C1_Q2_A7

---------------------------------------------------------------------------------------------------------------------------------------------------------------------------------------------------------------------------------------------------------------

type: numeric (double)

label: CPASS1

range: [1,6] units: 1

unique values: 5 missing .: 0/302

tabulation: Freq. Numeric Label

263 1 not at all

23 2 minimal

9 3 mild

6 4 moderate

1 6 extreme

---------------------------------------------------------------------------------------------------------------------------------------------------------------------------------------------------------------------------------------------------------------

C1_Q2_A8 (sum) C1_Q2_A8

---------------------------------------------------------------------------------------------------------------------------------------------------------------------------------------------------------------------------------------------------------------

type: numeric (double)

label: CPASS1

range: [1,5] units: 1

unique values: 5 missing .: 0/302

tabulation: Freq. Numeric Label

262 1 not at all

27 2 minimal

6 3 mild

6 4 moderate

1 5 severe

---------------------------------------------------------------------------------------------------------------------------------------------------------------------------------------------------------------------------------------------------------------

C1_Q2_A9 (sum) C1_Q2_A9

---------------------------------------------------------------------------------------------------------------------------------------------------------------------------------------------------------------------------------------------------------------

type: numeric (double)

label: CPASS1

range: [1,6] units: 1

unique values: 5 missing .: 0/302

tabulation: Freq. Numeric Label

266 1 not at all

25 2 minimal

5 3 mild

5 4 moderate

1 6 extreme

---------------------------------------------------------------------------------------------------------------------------------------------------------------------------------------------------------------------------------------------------------------

C1_Q2_A10 (sum) C1_Q2_A10

---------------------------------------------------------------------------------------------------------------------------------------------------------------------------------------------------------------------------------------------------------------

type: numeric (double)

label: CPASS1

range: [1,6] units: 1

unique values: 6 missing .: 0/302

tabulation: Freq. Numeric Label

267 1 not at all

20 2 minimal

8 3 mild

4 4 moderate

2 5 severe

1 6 extreme

---------------------------------------------------------------------------------------------------------------------------------------------------------------------------------------------------------------------------------------------------------------

C1_Q3_B1 (sum) C1_Q3_B1

---------------------------------------------------------------------------------------------------------------------------------------------------------------------------------------------------------------------------------------------------------------

type: numeric (double)

label: CPASS1, but 1 nonmissing value is not labeled

range: [0,5] units: 1

unique values: 6 missing .: 0/302

tabulation: Freq. Numeric Label

1 0

209 1 not at all

40 2 minimal

29 3 mild

17 4 moderate

6 5 severe

---------------------------------------------------------------------------------------------------------------------------------------------------------------------------------------------------------------------------------------------------------------

C1_Q3_B2 (sum) C1_Q3_B2

---------------------------------------------------------------------------------------------------------------------------------------------------------------------------------------------------------------------------------------------------------------

type: numeric (double)

label: CPASS1, but 1 nonmissing value is not labeled

range: [0,6] units: 1

unique values: 7 missing .: 0/302

tabulation: Freq. Numeric Label

1 0

215 1 not at all

48 2 minimal

18 3 mild

13 4 moderate

6 5 severe

1 6 extreme

---------------------------------------------------------------------------------------------------------------------------------------------------------------------------------------------------------------------------------------------------------------

C1_Q3_B3 (sum) C1_Q3_B3

---------------------------------------------------------------------------------------------------------------------------------------------------------------------------------------------------------------------------------------------------------------

type: numeric (double)

label: CPASS1

range: [1,6] units: 1

unique values: 6 missing .: 0/302

tabulation: Freq. Numeric Label

223 1 not at all

32 2 minimal

21 3 mild

16 4 moderate

8 5 severe

2 6 extreme

---------------------------------------------------------------------------------------------------------------------------------------------------------------------------------------------------------------------------------------------------------------

C1_Q3_B4 (sum) C1_Q3_B4

---------------------------------------------------------------------------------------------------------------------------------------------------------------------------------------------------------------------------------------------------------------

type: numeric (double)

label: CPASS1

range: [1,6] units: 1

unique values: 6 missing .: 0/302

tabulation: Freq. Numeric Label

210 1 not at all

44 2 minimal

28 3 mild

9 4 moderate

10 5 severe

1 6 extreme

---------------------------------------------------------------------------------------------------------------------------------------------------------------------------------------------------------------------------------------------------------------

C1_Q3_B5 (sum) C1_Q3_B5

---------------------------------------------------------------------------------------------------------------------------------------------------------------------------------------------------------------------------------------------------------------

type: numeric (double)

label: CPASS1, but 1 nonmissing value is not labeled

range: [0,6] units: 1

unique values: 7 missing .: 0/302

tabulation: Freq. Numeric Label

1 0

214 1 not at all

39 2 minimal

26 3 mild

16 4 moderate

5 5 severe

1 6 extreme

---------------------------------------------------------------------------------------------------------------------------------------------------------------------------------------------------------------------------------------------------------------

C1_Q3_B6 (sum) C1_Q3_B6

---------------------------------------------------------------------------------------------------------------------------------------------------------------------------------------------------------------------------------------------------------------

type: numeric (double)

label: CPASS1

range: [1,6] units: 1

unique values: 6 missing .: 0/302

tabulation: Freq. Numeric Label

219 1 not at all

38 2 minimal

33 3 mild

4 4 moderate

7 5 severe

1 6 extreme

---------------------------------------------------------------------------------------------------------------------------------------------------------------------------------------------------------------------------------------------------------------

C1_Q3_B7 (sum) C1_Q3_B7

---------------------------------------------------------------------------------------------------------------------------------------------------------------------------------------------------------------------------------------------------------------

type: numeric (double)

label: CPASS1, but 1 nonmissing value is not labeled

range: [0,6] units: 1

unique values: 7 missing .: 0/302

tabulation: Freq. Numeric Label

1 0

204 1 not at all

43 2 minimal

30 3 mild

17 4 moderate

2 5 severe

5 6 extreme

---------------------------------------------------------------------------------------------------------------------------------------------------------------------------------------------------------------------------------------------------------------

C1_Q3_A1 (sum) C1_Q3_A1

---------------------------------------------------------------------------------------------------------------------------------------------------------------------------------------------------------------------------------------------------------------

type: numeric (double)

label: CPASS1

range: [1,6] units: 1

unique values: 6 missing .: 0/302

tabulation: Freq. Numeric Label

207 1 not at all

46 2 minimal

24 3 mild

16 4 moderate

5 5 severe

4 6 extreme

---------------------------------------------------------------------------------------------------------------------------------------------------------------------------------------------------------------------------------------------------------------

C1_Q3_A2 (sum) C1_Q3_A2

---------------------------------------------------------------------------------------------------------------------------------------------------------------------------------------------------------------------------------------------------------------

type: numeric (double)

label: CPASS1

range: [1,6] units: 1

unique values: 6 missing .: 0/302

tabulation: Freq. Numeric Label

216 1 not at all

38 2 minimal

23 3 mild

16 4 moderate

5 5 severe

4 6 extreme

---------------------------------------------------------------------------------------------------------------------------------------------------------------------------------------------------------------------------------------------------------------

C1_Q3_A3 (sum) C1_Q3_A3

---------------------------------------------------------------------------------------------------------------------------------------------------------------------------------------------------------------------------------------------------------------

type: numeric (double)

label: CPASS1

range: [1,6] units: 1

unique values: 6 missing .: 0/302

tabulation: Freq. Numeric Label

207 1 not at all

44 2 minimal

24 3 mild

16 4 moderate

7 5 severe

4 6 extreme

---------------------------------------------------------------------------------------------------------------------------------------------------------------------------------------------------------------------------------------------------------------

C1_Q3_A4 (sum) C1_Q3_A4

---------------------------------------------------------------------------------------------------------------------------------------------------------------------------------------------------------------------------------------------------------------

type: numeric (double)

label: CPASS1

range: [1,6] units: 1

unique values: 6 missing .: 0/302

tabulation: Freq. Numeric Label

213 1 not at all

45 2 minimal

22 3 mild

14 4 moderate

5 5 severe

3 6 extreme

---------------------------------------------------------------------------------------------------------------------------------------------------------------------------------------------------------------------------------------------------------------

C1_Q3_A5 (sum) C1_Q3_A5

---------------------------------------------------------------------------------------------------------------------------------------------------------------------------------------------------------------------------------------------------------------

type: numeric (double)

label: CPASS1

range: [1,6] units: 1

unique values: 6 missing .: 0/302

tabulation: Freq. Numeric Label

225 1 not at all

45 2 minimal

14 3 mild

14 4 moderate

2 5 severe

2 6 extreme

---------------------------------------------------------------------------------------------------------------------------------------------------------------------------------------------------------------------------------------------------------------

C1_Q3_A6 (sum) C1_Q3_A6

---------------------------------------------------------------------------------------------------------------------------------------------------------------------------------------------------------------------------------------------------------------

type: numeric (double)

label: CPASS1

range: [1,5] units: 1

unique values: 5 missing .: 0/302

tabulation: Freq. Numeric Label

236 1 not at all

34 2 minimal

16 3 mild

12 4 moderate

4 5 severe

---------------------------------------------------------------------------------------------------------------------------------------------------------------------------------------------------------------------------------------------------------------

C1_Q3_A7 (sum) C1_Q3_A7

---------------------------------------------------------------------------------------------------------------------------------------------------------------------------------------------------------------------------------------------------------------

type: numeric (double)

label: CPASS1

range: [1,6] units: 1

unique values: 6 missing .: 0/302

tabulation: Freq. Numeric Label

242 1 not at all

29 2 minimal

18 3 mild

9 4 moderate

3 5 severe

1 6 extreme

---------------------------------------------------------------------------------------------------------------------------------------------------------------------------------------------------------------------------------------------------------------

C1_Q3_A8 (sum) C1_Q3_A8

---------------------------------------------------------------------------------------------------------------------------------------------------------------------------------------------------------------------------------------------------------------

type: numeric (double)

label: CPASS1

range: [1,5] units: 1

unique values: 5 missing .: 0/302

tabulation: Freq. Numeric Label

252 1 not at all

35 2 minimal

7 3 mild

5 4 moderate

3 5 severe

---------------------------------------------------------------------------------------------------------------------------------------------------------------------------------------------------------------------------------------------------------------

C1_Q3_A9 (sum) C1_Q3_A9

---------------------------------------------------------------------------------------------------------------------------------------------------------------------------------------------------------------------------------------------------------------

type: numeric (double)

label: CPASS1

range: [1,6] units: 1

unique values: 5 missing .: 0/302

tabulation: Freq. Numeric Label

250 1 not at all

31 2 minimal

12 3 mild

7 4 moderate

2 6 extreme

---------------------------------------------------------------------------------------------------------------------------------------------------------------------------------------------------------------------------------------------------------------

C1_Q3_A10 (sum) C1_Q3_A10

---------------------------------------------------------------------------------------------------------------------------------------------------------------------------------------------------------------------------------------------------------------

type: numeric (double)

label: CPASS1

range: [1,6] units: 1

unique values: 6 missing .: 0/302

tabulation: Freq. Numeric Label

256 1 not at all

23 2 minimal

10 3 mild

8 4 moderate

3 5 severe

2 6 extreme

---------------------------------------------------------------------------------------------------------------------------------------------------------------------------------------------------------------------------------------------------------------

C1_Q4_B1 (sum) C1_Q4_B1

---------------------------------------------------------------------------------------------------------------------------------------------------------------------------------------------------------------------------------------------------------------

type: numeric (double)

label: CPASS1, but 1 nonmissing value is not labeled

range: [0,6] units: 1

unique values: 7 missing .: 0/302

tabulation: Freq. Numeric Label

1 0

187 1 not at all

47 2 minimal

36 3 mild

21 4 moderate

9 5 severe

1 6 extreme

---------------------------------------------------------------------------------------------------------------------------------------------------------------------------------------------------------------------------------------------------------------

C1_Q4_B2 (sum) C1_Q4_B2

---------------------------------------------------------------------------------------------------------------------------------------------------------------------------------------------------------------------------------------------------------------

type: numeric (double)

label: CPASS1, but 1 nonmissing value is not labeled

range: [0,6] units: 1

unique values: 7 missing .: 0/302

tabulation: Freq. Numeric Label

1 0

180 1 not at all

58 2 minimal

34 3 mild

24 4 moderate

3 5 severe

2 6 extreme

---------------------------------------------------------------------------------------------------------------------------------------------------------------------------------------------------------------------------------------------------------------

C1_Q4_B3 (sum) C1_Q4_B3

---------------------------------------------------------------------------------------------------------------------------------------------------------------------------------------------------------------------------------------------------------------

type: numeric (double)

label: CPASS1

range: [1,6] units: 1

unique values: 6 missing .: 0/302

tabulation: Freq. Numeric Label

177 1 not at all

66 2 minimal

31 3 mild

21 4 moderate

5 5 severe

2 6 extreme

---------------------------------------------------------------------------------------------------------------------------------------------------------------------------------------------------------------------------------------------------------------

C1_Q4_B4 (sum) C1_Q4_B4

---------------------------------------------------------------------------------------------------------------------------------------------------------------------------------------------------------------------------------------------------------------

type: numeric (double)

label: CPASS1

range: [1,6] units: 1

unique values: 6 missing .: 0/302

tabulation: Freq. Numeric Label

184 1 not at all

56 2 minimal

34 3 mild

19 4 moderate

8 5 severe

1 6 extreme

---------------------------------------------------------------------------------------------------------------------------------------------------------------------------------------------------------------------------------------------------------------

C1_Q4_B5 (sum) C1_Q4_B5

---------------------------------------------------------------------------------------------------------------------------------------------------------------------------------------------------------------------------------------------------------------

type: numeric (double)

label: CPASS1, but 1 nonmissing value is not labeled

range: [0,6] units: 1

unique values: 7 missing .: 0/302

tabulation: Freq. Numeric Label

1 0

178 1 not at all

56 2 minimal

39 3 mild

22 4 moderate

4 5 severe

2 6 extreme

---------------------------------------------------------------------------------------------------------------------------------------------------------------------------------------------------------------------------------------------------------------

C1_Q4_B6 (sum) C1_Q4_B6

---------------------------------------------------------------------------------------------------------------------------------------------------------------------------------------------------------------------------------------------------------------

type: numeric (double)

label: CPASS1

range: [1,6] units: 1

unique values: 6 missing .: 0/302

tabulation: Freq. Numeric Label

174 1 not at all

60 2 minimal

41 3 mild

18 4 moderate

5 5 severe

4 6 extreme

---------------------------------------------------------------------------------------------------------------------------------------------------------------------------------------------------------------------------------------------------------------

C1_Q4_B7 (sum) C1_Q4_B7

---------------------------------------------------------------------------------------------------------------------------------------------------------------------------------------------------------------------------------------------------------------

type: numeric (double)

label: CPASS1, but 1 nonmissing value is not labeled

range: [0,6] units: 1

unique values: 7 missing .: 0/302

tabulation: Freq. Numeric Label

1 0

156 1 not at all

67 2 minimal

44 3 mild

24 4 moderate

6 5 severe

4 6 extreme

---------------------------------------------------------------------------------------------------------------------------------------------------------------------------------------------------------------------------------------------------------------

C1_Q4_A1 (sum) C1_Q4_A1

---------------------------------------------------------------------------------------------------------------------------------------------------------------------------------------------------------------------------------------------------------------

type: numeric (double)

label: CPASS1

range: [1,6] units: 1

unique values: 6 missing .: 0/302

tabulation: Freq. Numeric Label

136 1 not at all

75 2 minimal

43 3 mild

34 4 moderate

11 5 severe

3 6 extreme

---------------------------------------------------------------------------------------------------------------------------------------------------------------------------------------------------------------------------------------------------------------

C1_Q4_A2 (sum) C1_Q4_A2

---------------------------------------------------------------------------------------------------------------------------------------------------------------------------------------------------------------------------------------------------------------

type: numeric (double)

label: CPASS1

range: [1,6] units: 1

unique values: 6 missing .: 0/302

tabulation: Freq. Numeric Label

152 1 not at all

57 2 minimal

49 3 mild

24 4 moderate

15 5 severe

5 6 extreme

---------------------------------------------------------------------------------------------------------------------------------------------------------------------------------------------------------------------------------------------------------------

C1_Q4_A3 (sum) C1_Q4_A3

---------------------------------------------------------------------------------------------------------------------------------------------------------------------------------------------------------------------------------------------------------------

type: numeric (double)

label: CPASS1

range: [1,6] units: 1

unique values: 6 missing .: 0/302

tabulation: Freq. Numeric Label

158 1 not at all

62 2 minimal

47 3 mild

22 4 moderate

9 5 severe

4 6 extreme

---------------------------------------------------------------------------------------------------------------------------------------------------------------------------------------------------------------------------------------------------------------

C1_Q4_A4 (sum) C1_Q4_A4

---------------------------------------------------------------------------------------------------------------------------------------------------------------------------------------------------------------------------------------------------------------

type: numeric (double)

label: CPASS1

range: [1,6] units: 1

unique values: 6 missing .: 0/302

tabulation: Freq. Numeric Label

184 1 not at all

50 2 minimal

47 3 mild

14 4 moderate

5 5 severe

2 6 extreme

---------------------------------------------------------------------------------------------------------------------------------------------------------------------------------------------------------------------------------------------------------------

C1_Q4_A5 (sum) C1_Q4_A5

---------------------------------------------------------------------------------------------------------------------------------------------------------------------------------------------------------------------------------------------------------------

type: numeric (double)

label: CPASS1

range: [1,5] units: 1

unique values: 5 missing .: 0/302

tabulation: Freq. Numeric Label

198 1 not at all

55 2 minimal

28 3 mild

19 4 moderate

2 5 severe

---------------------------------------------------------------------------------------------------------------------------------------------------------------------------------------------------------------------------------------------------------------

C1_Q4_A6 (sum) C1_Q4_A6

---------------------------------------------------------------------------------------------------------------------------------------------------------------------------------------------------------------------------------------------------------------

type: numeric (double)

label: CPASS1

range: [1,5] units: 1

unique values: 5 missing .: 0/302

tabulation: Freq. Numeric Label

220 1 not at all

45 2 minimal

22 3 mild

13 4 moderate

2 5 severe

---------------------------------------------------------------------------------------------------------------------------------------------------------------------------------------------------------------------------------------------------------------

C1_Q4_A7 (sum) C1_Q4_A7

---------------------------------------------------------------------------------------------------------------------------------------------------------------------------------------------------------------------------------------------------------------

type: numeric (double)

label: CPASS1

range: [1,5] units: 1

unique values: 5 missing .: 0/302

tabulation: Freq. Numeric Label

232 1 not at all

39 2 minimal

20 3 mild

9 4 moderate

2 5 severe

---------------------------------------------------------------------------------------------------------------------------------------------------------------------------------------------------------------------------------------------------------------

C1_Q4_A8 (sum) C1_Q4_A8

---------------------------------------------------------------------------------------------------------------------------------------------------------------------------------------------------------------------------------------------------------------

type: numeric (double)

label: CPASS1

range: [1,6] units: 1

unique values: 6 missing .: 0/302

tabulation: Freq. Numeric Label

231 1 not at all

35 2 minimal

24 3 mild

9 4 moderate

2 5 severe

1 6 extreme

---------------------------------------------------------------------------------------------------------------------------------------------------------------------------------------------------------------------------------------------------------------

C1_Q4_A9 (sum) C1_Q4_A9

---------------------------------------------------------------------------------------------------------------------------------------------------------------------------------------------------------------------------------------------------------------

type: numeric (double)

label: CPASS1

range: [1,5] units: 1

unique values: 5 missing .: 0/302

tabulation: Freq. Numeric Label

222 1 not at all

52 2 minimal

16 3 mild

9 4 moderate

3 5 severe

---------------------------------------------------------------------------------------------------------------------------------------------------------------------------------------------------------------------------------------------------------------

C1_Q4_A10 (sum) C1_Q4_A10

---------------------------------------------------------------------------------------------------------------------------------------------------------------------------------------------------------------------------------------------------------------

type: numeric (double)

label: CPASS1

range: [1,6] units: 1

unique values: 6 missing .: 0/302

tabulation: Freq. Numeric Label

237 1 not at all

37 2 minimal

15 3 mild

8 4 moderate

4 5 severe

1 6 extreme

---------------------------------------------------------------------------------------------------------------------------------------------------------------------------------------------------------------------------------------------------------------

C1_Q5_B1 (sum) C1_Q5_B1

---------------------------------------------------------------------------------------------------------------------------------------------------------------------------------------------------------------------------------------------------------------

type: numeric (double)

label: CPASS1, but 1 nonmissing value is not labeled

range: [0,6] units: 1

unique values: 7 missing .: 0/302

tabulation: Freq. Numeric Label

1 0

178 1 not at all

55 2 minimal

33 3 mild

24 4 moderate

9 5 severe

2 6 extreme

---------------------------------------------------------------------------------------------------------------------------------------------------------------------------------------------------------------------------------------------------------------

C1_Q5_B2 (sum) C1_Q5_B2

---------------------------------------------------------------------------------------------------------------------------------------------------------------------------------------------------------------------------------------------------------------

type: numeric (double)

label: CPASS1, but 1 nonmissing value is not labeled

range: [0,6] units: 1

unique values: 7 missing .: 0/302

tabulation: Freq. Numeric Label

1 0

183 1 not at all

59 2 minimal

28 3 mild

21 4 moderate

9 5 severe

1 6 extreme

---------------------------------------------------------------------------------------------------------------------------------------------------------------------------------------------------------------------------------------------------------------

C1_Q5_B3 (sum) C1_Q5_B3

---------------------------------------------------------------------------------------------------------------------------------------------------------------------------------------------------------------------------------------------------------------

type: numeric (double)

label: CPASS1

range: [1,6] units: 1

unique values: 6 missing .: 0/302

tabulation: Freq. Numeric Label

188 1 not at all

56 2 minimal

26 3 mild

21 4 moderate

10 5 severe

1 6 extreme

---------------------------------------------------------------------------------------------------------------------------------------------------------------------------------------------------------------------------------------------------------------

C1_Q5_B4 (sum) C1_Q5_B4

---------------------------------------------------------------------------------------------------------------------------------------------------------------------------------------------------------------------------------------------------------------

type: numeric (double)

label: CPASS1

range: [1,6] units: 1

unique values: 6 missing .: 0/302

tabulation: Freq. Numeric Label

186 1 not at all

60 2 minimal

30 3 mild

19 4 moderate

6 5 severe

1 6 extreme

---------------------------------------------------------------------------------------------------------------------------------------------------------------------------------------------------------------------------------------------------------------

C1_Q5_B5 (sum) C1_Q5_B5

---------------------------------------------------------------------------------------------------------------------------------------------------------------------------------------------------------------------------------------------------------------

type: numeric (double)

label: CPASS1, but 1 nonmissing value is not labeled

range: [0,6] units: 1

unique values: 7 missing .: 0/302

tabulation: Freq. Numeric Label

1 0

168 1 not at all

69 2 minimal

41 3 mild

17 4 moderate

4 5 severe

2 6 extreme

---------------------------------------------------------------------------------------------------------------------------------------------------------------------------------------------------------------------------------------------------------------

C1_Q5_B6 (sum) C1_Q5_B6

---------------------------------------------------------------------------------------------------------------------------------------------------------------------------------------------------------------------------------------------------------------

type: numeric (double)

label: CPASS1

range: [1,6] units: 1

unique values: 6 missing .: 0/302

tabulation: Freq. Numeric Label

178 1 not at all

58 2 minimal

40 3 mild

16 4 moderate

7 5 severe

3 6 extreme

---------------------------------------------------------------------------------------------------------------------------------------------------------------------------------------------------------------------------------------------------------------

C1_Q5_B7 (sum) C1_Q5_B7

---------------------------------------------------------------------------------------------------------------------------------------------------------------------------------------------------------------------------------------------------------------

type: numeric (double)

label: CPASS1, but 1 nonmissing value is not labeled

range: [0,6] units: 1

unique values: 7 missing .: 0/302

tabulation: Freq. Numeric Label

1 0

160 1 not at all

61 2 minimal

48 3 mild

21 4 moderate

6 5 severe

5 6 extreme

---------------------------------------------------------------------------------------------------------------------------------------------------------------------------------------------------------------------------------------------------------------

C1_Q5_A1 (sum) C1_Q5_A1

---------------------------------------------------------------------------------------------------------------------------------------------------------------------------------------------------------------------------------------------------------------

type: numeric (double)

label: CPASS1

range: [1,6] units: 1

unique values: 6 missing .: 0/302

tabulation: Freq. Numeric Label

137 1 not at all

68 2 minimal

51 3 mild

33 4 moderate

10 5 severe

3 6 extreme

---------------------------------------------------------------------------------------------------------------------------------------------------------------------------------------------------------------------------------------------------------------

C1_Q5_A2 (sum) C1_Q5_A2

---------------------------------------------------------------------------------------------------------------------------------------------------------------------------------------------------------------------------------------------------------------

type: numeric (double)

label: CPASS1

range: [1,6] units: 1

unique values: 6 missing .: 0/302

tabulation: Freq. Numeric Label

153 1 not at all

60 2 minimal

45 3 mild

29 4 moderate

10 5 severe

5 6 extreme

---------------------------------------------------------------------------------------------------------------------------------------------------------------------------------------------------------------------------------------------------------------

C1_Q5_A3 (sum) C1_Q5_A3

---------------------------------------------------------------------------------------------------------------------------------------------------------------------------------------------------------------------------------------------------------------

type: numeric (double)

label: CPASS1

range: [1,6] units: 1

unique values: 6 missing .: 0/302

tabulation: Freq. Numeric Label

164 1 not at all

69 2 minimal

35 3 mild

26 4 moderate

4 5 severe

4 6 extreme

---------------------------------------------------------------------------------------------------------------------------------------------------------------------------------------------------------------------------------------------------------------

C1_Q5_A4 (sum) C1_Q5_A4

---------------------------------------------------------------------------------------------------------------------------------------------------------------------------------------------------------------------------------------------------------------

type: numeric (double)

label: CPASS1

range: [1,6] units: 1

unique values: 6 missing .: 0/302

tabulation: Freq. Numeric Label

189 1 not at all

59 2 minimal

33 3 mild

14 4 moderate

6 5 severe

1 6 extreme

---------------------------------------------------------------------------------------------------------------------------------------------------------------------------------------------------------------------------------------------------------------

C1_Q5_A5 (sum) C1_Q5_A5

---------------------------------------------------------------------------------------------------------------------------------------------------------------------------------------------------------------------------------------------------------------

type: numeric (double)

label: CPASS1

range: [1,6] units: 1

unique values: 5 missing .: 0/302

tabulation: Freq. Numeric Label

205 1 not at all

48 2 minimal

32 3 mild

15 4 moderate

2 6 extreme

---------------------------------------------------------------------------------------------------------------------------------------------------------------------------------------------------------------------------------------------------------------

C1_Q5_A6 (sum) C1_Q5_A6

---------------------------------------------------------------------------------------------------------------------------------------------------------------------------------------------------------------------------------------------------------------

type: numeric (double)

label: CPASS1

range: [1,5] units: 1

unique values: 5 missing .: 0/302

tabulation: Freq. Numeric Label

220 1 not at all

44 2 minimal

21 3 mild

13 4 moderate

4 5 severe

---------------------------------------------------------------------------------------------------------------------------------------------------------------------------------------------------------------------------------------------------------------

C1_Q5_A7 (sum) C1_Q5_A7

---------------------------------------------------------------------------------------------------------------------------------------------------------------------------------------------------------------------------------------------------------------

type: numeric (double)

label: CPASS1

range: [1,4] units: 1

unique values: 4 missing .: 0/302

tabulation: Freq. Numeric Label

233 1 not at all

42 2 minimal

22 3 mild

5 4 moderate

---------------------------------------------------------------------------------------------------------------------------------------------------------------------------------------------------------------------------------------------------------------

C1_Q5_A8 (sum) C1_Q5_A8

---------------------------------------------------------------------------------------------------------------------------------------------------------------------------------------------------------------------------------------------------------------

type: numeric (double)

label: CPASS1

range: [1,6] units: 1

unique values: 6 missing .: 0/302

tabulation: Freq. Numeric Label

228 1 not at all

43 2 minimal

18 3 mild

10 4 moderate

2 5 severe

1 6 extreme

---------------------------------------------------------------------------------------------------------------------------------------------------------------------------------------------------------------------------------------------------------------

C1_Q5_A9 (sum) C1_Q5_A9

---------------------------------------------------------------------------------------------------------------------------------------------------------------------------------------------------------------------------------------------------------------

type: numeric (double)

label: CPASS1

range: [1,5] units: 1

unique values: 5 missing .: 0/302

tabulation: Freq. Numeric Label

235 1 not at all

42 2 minimal

18 3 mild

5 4 moderate

2 5 severe

---------------------------------------------------------------------------------------------------------------------------------------------------------------------------------------------------------------------------------------------------------------

C1_Q5_A10 (sum) C1_Q5_A10

---------------------------------------------------------------------------------------------------------------------------------------------------------------------------------------------------------------------------------------------------------------

type: numeric (double)

label: CPASS1

range: [1,6] units: 1

unique values: 6 missing .: 0/302

tabulation: Freq. Numeric Label

234 1 not at all

39 2 minimal

16 3 mild

11 4 moderate

1 5 severe

1 6 extreme

---------------------------------------------------------------------------------------------------------------------------------------------------------------------------------------------------------------------------------------------------------------

C1_Q6_B1 (sum) C1_Q6_B1

---------------------------------------------------------------------------------------------------------------------------------------------------------------------------------------------------------------------------------------------------------------

type: numeric (double)

label: CPASS1, but 1 nonmissing value is not labeled

range: [0,6] units: 1

unique values: 7 missing .: 0/302

tabulation: Freq. Numeric Label

1 0

206 1 not at all

46 2 minimal

19 3 mild

25 4 moderate

2 5 severe

3 6 extreme

---------------------------------------------------------------------------------------------------------------------------------------------------------------------------------------------------------------------------------------------------------------

C1_Q6_B2 (sum) C1_Q6_B2

---------------------------------------------------------------------------------------------------------------------------------------------------------------------------------------------------------------------------------------------------------------

type: numeric (double)

label: CPASS1, but 1 nonmissing value is not labeled

range: [0,6] units: 1

unique values: 7 missing .: 0/302

tabulation: Freq. Numeric Label

1 0

202 1 not at all

49 2 minimal

25 3 mild

17 4 moderate

5 5 severe

3 6 extreme

---------------------------------------------------------------------------------------------------------------------------------------------------------------------------------------------------------------------------------------------------------------

C1_Q6_B3 (sum) C1_Q6_B3

---------------------------------------------------------------------------------------------------------------------------------------------------------------------------------------------------------------------------------------------------------------

type: numeric (double)

label: CPASS1

range: [1,6] units: 1

unique values: 6 missing .: 0/302

tabulation: Freq. Numeric Label

205 1 not at all

49 2 minimal

24 3 mild

16 4 moderate

6 5 severe

2 6 extreme

---------------------------------------------------------------------------------------------------------------------------------------------------------------------------------------------------------------------------------------------------------------

C1_Q6_B4 (sum) C1_Q6_B4

---------------------------------------------------------------------------------------------------------------------------------------------------------------------------------------------------------------------------------------------------------------

type: numeric (double)

label: CPASS1

range: [1,6] units: 1

unique values: 6 missing .: 0/302

tabulation: Freq. Numeric Label

209 1 not at all

44 2 minimal

29 3 mild

16 4 moderate

3 5 severe

1 6 extreme

---------------------------------------------------------------------------------------------------------------------------------------------------------------------------------------------------------------------------------------------------------------

C1_Q6_B5 (sum) C1_Q6_B5

---------------------------------------------------------------------------------------------------------------------------------------------------------------------------------------------------------------------------------------------------------------

type: numeric (double)

label: CPASS1, but 1 nonmissing value is not labeled

range: [0,6] units: 1

unique values: 7 missing .: 0/302

tabulation: Freq. Numeric Label

1 0

196 1 not at all

57 2 minimal

28 3 mild

14 4 moderate

4 5 severe

2 6 extreme

---------------------------------------------------------------------------------------------------------------------------------------------------------------------------------------------------------------------------------------------------------------

C1_Q6_B6 (sum) C1_Q6_B6

---------------------------------------------------------------------------------------------------------------------------------------------------------------------------------------------------------------------------------------------------------------

type: numeric (double)

label: CPASS1

range: [1,6] units: 1

unique values: 6 missing .: 0/302

tabulation: Freq. Numeric Label

212 1 not at all

38 2 minimal

32 3 mild

11 4 moderate

6 5 severe

3 6 extreme

---------------------------------------------------------------------------------------------------------------------------------------------------------------------------------------------------------------------------------------------------------------

C1_Q6_B7 (sum) C1_Q6_B7

---------------------------------------------------------------------------------------------------------------------------------------------------------------------------------------------------------------------------------------------------------------

type: numeric (double)

label: CPASS1, but 1 nonmissing value is not labeled

range: [0,6] units: 1

unique values: 7 missing .: 0/302

tabulation: Freq. Numeric Label

1 0

190 1 not at all

45 2 minimal

36 3 mild

19 4 moderate

6 5 severe

5 6 extreme

---------------------------------------------------------------------------------------------------------------------------------------------------------------------------------------------------------------------------------------------------------------

C1_Q6_A1 (sum) C1_Q6_A1

---------------------------------------------------------------------------------------------------------------------------------------------------------------------------------------------------------------------------------------------------------------

type: numeric (double)

label: CPASS1

range: [1,6] units: 1

unique values: 6 missing .: 0/302

tabulation: Freq. Numeric Label

173 1 not at all

53 2 minimal

38 3 mild

26 4 moderate

10 5 severe

2 6 extreme

---------------------------------------------------------------------------------------------------------------------------------------------------------------------------------------------------------------------------------------------------------------

C1_Q6_A2 (sum) C1_Q6_A2

---------------------------------------------------------------------------------------------------------------------------------------------------------------------------------------------------------------------------------------------------------------

type: numeric (double)

label: CPASS1

range: [1,6] units: 1

unique values: 6 missing .: 0/302

tabulation: Freq. Numeric Label

184 1 not at all

49 2 minimal

30 3 mild

26 4 moderate

8 5 severe

5 6 extreme

---------------------------------------------------------------------------------------------------------------------------------------------------------------------------------------------------------------------------------------------------------------

C1_Q6_A3 (sum) C1_Q6_A3

---------------------------------------------------------------------------------------------------------------------------------------------------------------------------------------------------------------------------------------------------------------

type: numeric (double)

label: CPASS1

range: [1,6] units: 1

unique values: 6 missing .: 0/302

tabulation: Freq. Numeric Label

187 1 not at all

53 2 minimal

31 3 mild

21 4 moderate

6 5 severe

4 6 extreme

---------------------------------------------------------------------------------------------------------------------------------------------------------------------------------------------------------------------------------------------------------------

C1_Q6_A4 (sum) C1_Q6_A4

---------------------------------------------------------------------------------------------------------------------------------------------------------------------------------------------------------------------------------------------------------------

type: numeric (double)

label: CPASS1

range: [1,6] units: 1

unique values: 6 missing .: 0/302

tabulation: Freq. Numeric Label

215 1 not at all

36 2 minimal

32 3 mild

13 4 moderate

3 5 severe

3 6 extreme

---------------------------------------------------------------------------------------------------------------------------------------------------------------------------------------------------------------------------------------------------------------

C1_Q6_A5 (sum) C1_Q6_A5

---------------------------------------------------------------------------------------------------------------------------------------------------------------------------------------------------------------------------------------------------------------

type: numeric (double)

label: CPASS1

range: [1,6] units: 1

unique values: 6 missing .: 0/302

tabulation: Freq. Numeric Label

223 1 not at all

44 2 minimal

22 3 mild

10 4 moderate

2 5 severe

1 6 extreme

---------------------------------------------------------------------------------------------------------------------------------------------------------------------------------------------------------------------------------------------------------------

C1_Q6_A6 (sum) C1_Q6_A6

---------------------------------------------------------------------------------------------------------------------------------------------------------------------------------------------------------------------------------------------------------------

type: numeric (double)

label: CPASS1

range: [1,5] units: 1

unique values: 5 missing .: 0/302

tabulation: Freq. Numeric Label

235 1 not at all

33 2 minimal

21 3 mild

10 4 moderate

3 5 severe

---------------------------------------------------------------------------------------------------------------------------------------------------------------------------------------------------------------------------------------------------------------

C1_Q6_A7 (sum) C1_Q6_A7

---------------------------------------------------------------------------------------------------------------------------------------------------------------------------------------------------------------------------------------------------------------

type: numeric (double)

label: CPASS1

range: [1,4] units: 1

unique values: 4 missing .: 0/302

tabulation: Freq. Numeric Label

252 1 not at all

35 2 minimal

7 3 mild

8 4 moderate

---------------------------------------------------------------------------------------------------------------------------------------------------------------------------------------------------------------------------------------------------------------

C1_Q6_A8 (sum) C1_Q6_A8

---------------------------------------------------------------------------------------------------------------------------------------------------------------------------------------------------------------------------------------------------------------

type: numeric (double)

label: CPASS1

range: [1,5] units: 1

unique values: 5 missing .: 0/302

tabulation: Freq. Numeric Label

251 1 not at all

32 2 minimal

11 3 mild

6 4 moderate

2 5 severe

---------------------------------------------------------------------------------------------------------------------------------------------------------------------------------------------------------------------------------------------------------------

C1_Q6_A9 (sum) C1_Q6_A9

---------------------------------------------------------------------------------------------------------------------------------------------------------------------------------------------------------------------------------------------------------------

type: numeric (double)

label: CPASS1

range: [1,5] units: 1

unique values: 5 missing .: 0/302

tabulation: Freq. Numeric Label

253 1 not at all

34 2 minimal

10 3 mild

3 4 moderate

2 5 severe

---------------------------------------------------------------------------------------------------------------------------------------------------------------------------------------------------------------------------------------------------------------

C1_Q6_A10 (sum) C1_Q6_A10

---------------------------------------------------------------------------------------------------------------------------------------------------------------------------------------------------------------------------------------------------------------

type: numeric (double)

label: CPASS1

range: [1,6] units: 1

unique values: 6 missing .: 0/302

tabulation: Freq. Numeric Label

252 1 not at all

27 2 minimal

16 3 mild

5 4 moderate

1 5 severe

1 6 extreme

---------------------------------------------------------------------------------------------------------------------------------------------------------------------------------------------------------------------------------------------------------------

C1_Q7_B1 (sum) C1_Q7_B1

---------------------------------------------------------------------------------------------------------------------------------------------------------------------------------------------------------------------------------------------------------------

type: numeric (double)

label: CPASS1, but 1 nonmissing value is not labeled

range: [0,6] units: 1

unique values: 7 missing .: 0/302

tabulation: Freq. Numeric Label

1 0

198 1 not at all

45 2 minimal

32 3 mild

19 4 moderate

5 5 severe

2 6 extreme

---------------------------------------------------------------------------------------------------------------------------------------------------------------------------------------------------------------------------------------------------------------

C1_Q7_B2 (sum) C1_Q7_B2

---------------------------------------------------------------------------------------------------------------------------------------------------------------------------------------------------------------------------------------------------------------

type: numeric (double)

label: CPASS1, but 1 nonmissing value is not labeled

range: [0,6] units: 1

unique values: 7 missing .: 0/302

tabulation: Freq. Numeric Label

1 0

200 1 not at all

48 2 minimal

25 3 mild

17 4 moderate

7 5 severe

4 6 extreme

---------------------------------------------------------------------------------------------------------------------------------------------------------------------------------------------------------------------------------------------------------------

C1_Q7_B3 (sum) C1_Q7_B3

---------------------------------------------------------------------------------------------------------------------------------------------------------------------------------------------------------------------------------------------------------------

type: numeric (double)

label: CPASS1

range: [1,6] units: 1

unique values: 6 missing .: 0/302

tabulation: Freq. Numeric Label

200 1 not at all

42 2 minimal

38 3 mild

13 4 moderate

7 5 severe

2 6 extreme

---------------------------------------------------------------------------------------------------------------------------------------------------------------------------------------------------------------------------------------------------------------

C1_Q7_B4 (sum) C1_Q7_B4

---------------------------------------------------------------------------------------------------------------------------------------------------------------------------------------------------------------------------------------------------------------

type: numeric (double)

label: CPASS1

range: [1,6] units: 1

unique values: 6 missing .: 0/302

tabulation: Freq. Numeric Label

201 1 not at all

46 2 minimal

32 3 mild

15 4 moderate

7 5 severe

1 6 extreme

---------------------------------------------------------------------------------------------------------------------------------------------------------------------------------------------------------------------------------------------------------------

C1_Q7_B5 (sum) C1_Q7_B5

---------------------------------------------------------------------------------------------------------------------------------------------------------------------------------------------------------------------------------------------------------------

type: numeric (double)

label: CPASS1, but 1 nonmissing value is not labeled

range: [0,6] units: 1

unique values: 7 missing .: 0/302

tabulation: Freq. Numeric Label

1 0

187 1 not at all

48 2 minimal

39 3 mild

21 4 moderate

4 5 severe

2 6 extreme

---------------------------------------------------------------------------------------------------------------------------------------------------------------------------------------------------------------------------------------------------------------

C1_Q7_B6 (sum) C1_Q7_B6

---------------------------------------------------------------------------------------------------------------------------------------------------------------------------------------------------------------------------------------------------------------

type: numeric (double)

label: CPASS1

range: [1,6] units: 1

unique values: 6 missing .: 0/302

tabulation: Freq. Numeric Label

198 1 not at all

44 2 minimal

34 3 mild

14 4 moderate

9 5 severe

3 6 extreme

---------------------------------------------------------------------------------------------------------------------------------------------------------------------------------------------------------------------------------------------------------------

C1_Q7_B7 (sum) C1_Q7_B7

---------------------------------------------------------------------------------------------------------------------------------------------------------------------------------------------------------------------------------------------------------------

type: numeric (double)

label: CPASS1, but 1 nonmissing value is not labeled

range: [0,6] units: 1

unique values: 7 missing .: 0/302

tabulation: Freq. Numeric Label

1 0

181 1 not at all

50 2 minimal

36 3 mild

23 4 moderate

7 5 severe

4 6 extreme

---------------------------------------------------------------------------------------------------------------------------------------------------------------------------------------------------------------------------------------------------------------

C1_Q7_A1 (sum) C1_Q7_A1

---------------------------------------------------------------------------------------------------------------------------------------------------------------------------------------------------------------------------------------------------------------

type: numeric (double)

label: CPASS1

range: [1,6] units: 1

unique values: 6 missing .: 0/302

tabulation: Freq. Numeric Label

156 1 not at all

58 2 minimal

37 3 mild

33 4 moderate

15 5 severe

3 6 extreme

---------------------------------------------------------------------------------------------------------------------------------------------------------------------------------------------------------------------------------------------------------------

C1_Q7_A2 (sum) C1_Q7_A2

---------------------------------------------------------------------------------------------------------------------------------------------------------------------------------------------------------------------------------------------------------------

type: numeric (double)

label: CPASS1

range: [1,6] units: 1

unique values: 6 missing .: 0/302

tabulation: Freq. Numeric Label

172 1 not at all

47 2 minimal

37 3 mild

25 4 moderate

15 5 severe

6 6 extreme

---------------------------------------------------------------------------------------------------------------------------------------------------------------------------------------------------------------------------------------------------------------

C1_Q7_A3 (sum) C1_Q7_A3

---------------------------------------------------------------------------------------------------------------------------------------------------------------------------------------------------------------------------------------------------------------

type: numeric (double)

label: CPASS1

range: [1,6] units: 1

unique values: 6 missing .: 0/302

tabulation: Freq. Numeric Label

179 1 not at all

49 2 minimal

41 3 mild

24 4 moderate

5 5 severe

4 6 extreme

---------------------------------------------------------------------------------------------------------------------------------------------------------------------------------------------------------------------------------------------------------------

C1_Q7_A4 (sum) C1_Q7_A4

---------------------------------------------------------------------------------------------------------------------------------------------------------------------------------------------------------------------------------------------------------------

type: numeric (double)

label: CPASS1

range: [1,6] units: 1

unique values: 6 missing .: 0/302

tabulation: Freq. Numeric Label

206 1 not at all

41 2 minimal

30 3 mild

18 4 moderate

6 5 severe

1 6 extreme

---------------------------------------------------------------------------------------------------------------------------------------------------------------------------------------------------------------------------------------------------------------

C1_Q7_A5 (sum) C1_Q7_A5

---------------------------------------------------------------------------------------------------------------------------------------------------------------------------------------------------------------------------------------------------------------

type: numeric (double)

label: CPASS1

range: [1,6] units: 1

unique values: 6 missing .: 0/302

tabulation: Freq. Numeric Label

219 1 not at all

41 2 minimal

23 3 mild

12 4 moderate

6 5 severe

1 6 extreme

---------------------------------------------------------------------------------------------------------------------------------------------------------------------------------------------------------------------------------------------------------------

C1_Q7_A6 (sum) C1_Q7_A6

---------------------------------------------------------------------------------------------------------------------------------------------------------------------------------------------------------------------------------------------------------------

type: numeric (double)

label: CPASS1

range: [1,5] units: 1

unique values: 5 missing .: 0/302

tabulation: Freq. Numeric Label

236 1 not at all

34 2 minimal

20 3 mild

6 4 moderate

6 5 severe

---------------------------------------------------------------------------------------------------------------------------------------------------------------------------------------------------------------------------------------------------------------

C1_Q7_A7 (sum) C1_Q7_A7

---------------------------------------------------------------------------------------------------------------------------------------------------------------------------------------------------------------------------------------------------------------

type: numeric (double)

label: CPASS1

range: [1,5] units: 1

unique values: 5 missing .: 0/302

tabulation: Freq. Numeric Label

245 1 not at all

40 2 minimal

12 3 mild

4 4 moderate

1 5 severe

---------------------------------------------------------------------------------------------------------------------------------------------------------------------------------------------------------------------------------------------------------------

C1_Q7_A8 (sum) C1_Q7_A8

---------------------------------------------------------------------------------------------------------------------------------------------------------------------------------------------------------------------------------------------------------------

type: numeric (double)

label: CPASS1

range: [1,6] units: 1

unique values: 6 missing .: 0/302

tabulation: Freq. Numeric Label

252 1 not at all

29 2 minimal

13 3 mild

5 4 moderate

2 5 severe

1 6 extreme

---------------------------------------------------------------------------------------------------------------------------------------------------------------------------------------------------------------------------------------------------------------

C1_Q7_A9 (sum) C1_Q7_A9

---------------------------------------------------------------------------------------------------------------------------------------------------------------------------------------------------------------------------------------------------------------

type: numeric (double)

label: CPASS1

range: [1,5] units: 1

unique values: 5 missing .: 0/302

tabulation: Freq. Numeric Label

253 1 not at all

29 2 minimal

14 3 mild

4 4 moderate

2 5 severe

---------------------------------------------------------------------------------------------------------------------------------------------------------------------------------------------------------------------------------------------------------------

C1_Q7_A10 (sum) C1_Q7_A10

---------------------------------------------------------------------------------------------------------------------------------------------------------------------------------------------------------------------------------------------------------------

type: numeric (double)

label: CPASS1

range: [1,4] units: 1

unique values: 4 missing .: 0/302

tabulation: Freq. Numeric Label

252 1 not at all

33 2 minimal

10 3 mild

7 4 moderate

---------------------------------------------------------------------------------------------------------------------------------------------------------------------------------------------------------------------------------------------------------------

C1_Q8_B1 (sum) C1_Q8_B1

---------------------------------------------------------------------------------------------------------------------------------------------------------------------------------------------------------------------------------------------------------------

type: numeric (double)

label: CPASS1, but 1 nonmissing value is not labeled

range: [0,6] units: 1

unique values: 7 missing .: 0/302

tabulation: Freq. Numeric Label

1 0

231 1 not at all

28 2 minimal

22 3 mild

14 4 moderate

5 5 severe

1 6 extreme

---------------------------------------------------------------------------------------------------------------------------------------------------------------------------------------------------------------------------------------------------------------

C1_Q8_B2 (sum) C1_Q8_B2

---------------------------------------------------------------------------------------------------------------------------------------------------------------------------------------------------------------------------------------------------------------

type: numeric (double)

label: CPASS1, but 1 nonmissing value is not labeled

range: [0,6] units: 1

unique values: 7 missing .: 0/302

tabulation: Freq. Numeric Label

1 0

232 1 not at all

23 2 minimal

25 3 mild

15 4 moderate

5 5 severe

1 6 extreme

---------------------------------------------------------------------------------------------------------------------------------------------------------------------------------------------------------------------------------------------------------------

C1_Q8_B3 (sum) C1_Q8_B3

---------------------------------------------------------------------------------------------------------------------------------------------------------------------------------------------------------------------------------------------------------------

type: numeric (double)

label: CPASS1

range: [1,6] units: 1

unique values: 6 missing .: 0/302

tabulation: Freq. Numeric Label

235 1 not at all

29 2 minimal

24 3 mild

8 4 moderate

4 5 severe

2 6 extreme

---------------------------------------------------------------------------------------------------------------------------------------------------------------------------------------------------------------------------------------------------------------

C1_Q8_B4 (sum) C1_Q8_B4

---------------------------------------------------------------------------------------------------------------------------------------------------------------------------------------------------------------------------------------------------------------

type: numeric (double)

label: CPASS1

range: [1,6] units: 1

unique values: 6 missing .: 0/302

tabulation: Freq. Numeric Label

240 1 not at all

28 2 minimal

21 3 mild

9 4 moderate

3 5 severe

1 6 extreme

---------------------------------------------------------------------------------------------------------------------------------------------------------------------------------------------------------------------------------------------------------------

C1_Q8_B5 (sum) C1_Q8_B5

---------------------------------------------------------------------------------------------------------------------------------------------------------------------------------------------------------------------------------------------------------------

type: numeric (double)

label: CPASS1, but 1 nonmissing value is not labeled

range: [0,6] units: 1

unique values: 7 missing .: 0/302

tabulation: Freq. Numeric Label

1 0

228 1 not at all

27 2 minimal

33 3 mild

11 4 moderate

1 5 severe

1 6 extreme

---------------------------------------------------------------------------------------------------------------------------------------------------------------------------------------------------------------------------------------------------------------

C1_Q8_B6 (sum) C1_Q8_B6

---------------------------------------------------------------------------------------------------------------------------------------------------------------------------------------------------------------------------------------------------------------

type: numeric (double)

label: CPASS1

range: [1,6] units: 1

unique values: 6 missing .: 0/302

tabulation: Freq. Numeric Label

231 1 not at all

35 2 minimal

21 3 mild

10 4 moderate

4 5 severe

1 6 extreme

---------------------------------------------------------------------------------------------------------------------------------------------------------------------------------------------------------------------------------------------------------------

C1_Q8_B7 (sum) C1_Q8_B7

---------------------------------------------------------------------------------------------------------------------------------------------------------------------------------------------------------------------------------------------------------------

type: numeric (double)

label: CPASS1, but 1 nonmissing value is not labeled

range: [0,6] units: 1

unique values: 7 missing .: 0/302

tabulation: Freq. Numeric Label

1 0

238 1 not at all

27 2 minimal

18 3 mild

14 4 moderate

2 5 severe

2 6 extreme

---------------------------------------------------------------------------------------------------------------------------------------------------------------------------------------------------------------------------------------------------------------

C1_Q8_A1 (sum) C1_Q8_A1

---------------------------------------------------------------------------------------------------------------------------------------------------------------------------------------------------------------------------------------------------------------

type: numeric (double)

label: CPASS1

range: [1,6] units: 1

unique values: 6 missing .: 0/302

tabulation: Freq. Numeric Label

227 1 not at all

33 2 minimal

24 3 mild

11 4 moderate

5 5 severe

2 6 extreme

---------------------------------------------------------------------------------------------------------------------------------------------------------------------------------------------------------------------------------------------------------------

C1_Q8_A2 (sum) C1_Q8_A2

---------------------------------------------------------------------------------------------------------------------------------------------------------------------------------------------------------------------------------------------------------------

type: numeric (double)

label: CPASS1

range: [1,6] units: 1

unique values: 6 missing .: 0/302

tabulation: Freq. Numeric Label

223 1 not at all

26 2 minimal

33 3 mild

13 4 moderate

5 5 severe

2 6 extreme

---------------------------------------------------------------------------------------------------------------------------------------------------------------------------------------------------------------------------------------------------------------

C1_Q8_A3 (sum) C1_Q8_A3

---------------------------------------------------------------------------------------------------------------------------------------------------------------------------------------------------------------------------------------------------------------

type: numeric (double)

label: CPASS1

range: [1,5] units: 1

unique values: 5 missing .: 0/302

tabulation: Freq. Numeric Label

232 1 not at all

28 2 minimal

20 3 mild

17 4 moderate

5 5 severe

---------------------------------------------------------------------------------------------------------------------------------------------------------------------------------------------------------------------------------------------------------------

C1_Q8_A4 (sum) C1_Q8_A4

---------------------------------------------------------------------------------------------------------------------------------------------------------------------------------------------------------------------------------------------------------------

type: numeric (double)

label: CPASS1

range: [1,5] units: 1

unique values: 5 missing .: 0/302

tabulation: Freq. Numeric Label

237 1 not at all

23 2 minimal

26 3 mild

10 4 moderate

6 5 severe

---------------------------------------------------------------------------------------------------------------------------------------------------------------------------------------------------------------------------------------------------------------

C1_Q8_A5 (sum) C1_Q8_A5

---------------------------------------------------------------------------------------------------------------------------------------------------------------------------------------------------------------------------------------------------------------

type: numeric (double)

label: CPASS1

range: [1,5] units: 1

unique values: 5 missing .: 0/302

tabulation: Freq. Numeric Label

245 1 not at all

32 2 minimal

13 3 mild

11 4 moderate

1 5 severe

---------------------------------------------------------------------------------------------------------------------------------------------------------------------------------------------------------------------------------------------------------------

C1_Q8_A6 (sum) C1_Q8_A6

---------------------------------------------------------------------------------------------------------------------------------------------------------------------------------------------------------------------------------------------------------------

type: numeric (double)

label: CPASS1

range: [1,5] units: 1

unique values: 5 missing .: 0/302

tabulation: Freq. Numeric Label

248 1 not at all

26 2 minimal

17 3 mild

10 4 moderate

1 5 severe

---------------------------------------------------------------------------------------------------------------------------------------------------------------------------------------------------------------------------------------------------------------

C1_Q8_A7 (sum) C1_Q8_A7

---------------------------------------------------------------------------------------------------------------------------------------------------------------------------------------------------------------------------------------------------------------

type: numeric (double)

label: CPASS1

range: [1,5] units: 1

unique values: 5 missing .: 0/302

tabulation: Freq. Numeric Label

266 1 not at all

21 2 minimal

9 3 mild

5 4 moderate

1 5 severe

---------------------------------------------------------------------------------------------------------------------------------------------------------------------------------------------------------------------------------------------------------------

C1_Q8_A8 (sum) C1_Q8_A8

---------------------------------------------------------------------------------------------------------------------------------------------------------------------------------------------------------------------------------------------------------------

type: numeric (double)

label: CPASS1

range: [1,6] units: 1

unique values: 5 missing .: 0/302

tabulation: Freq. Numeric Label

272 1 not at all

16 2 minimal

9 3 mild

4 4 moderate

1 6 extreme

---------------------------------------------------------------------------------------------------------------------------------------------------------------------------------------------------------------------------------------------------------------

C1_Q8_A9 (sum) C1_Q8_A9

---------------------------------------------------------------------------------------------------------------------------------------------------------------------------------------------------------------------------------------------------------------

type: numeric (double)

label: CPASS1

range: [1,5] units: 1

unique values: 5 missing .: 0/302

tabulation: Freq. Numeric Label

268 1 not at all

23 2 minimal

4 3 mild

4 4 moderate

3 5 severe

---------------------------------------------------------------------------------------------------------------------------------------------------------------------------------------------------------------------------------------------------------------

C1_Q8_A10 (sum) C1_Q8_A10

---------------------------------------------------------------------------------------------------------------------------------------------------------------------------------------------------------------------------------------------------------------

type: numeric (double)

label: CPASS1

range: [1,5] units: 1

unique values: 5 missing .: 0/302

tabulation: Freq. Numeric Label

266 1 not at all

26 2 minimal

6 3 mild

2 4 moderate

2 5 severe

---------------------------------------------------------------------------------------------------------------------------------------------------------------------------------------------------------------------------------------------------------------

C1_Q9_B1 (sum) C1_Q9_B1

---------------------------------------------------------------------------------------------------------------------------------------------------------------------------------------------------------------------------------------------------------------

type: numeric (double)

label: CPASS1, but 1 nonmissing value is not labeled

range: [0,6] units: 1

unique values: 7 missing .: 0/302

tabulation: Freq. Numeric Label

1 0

211 1 not at all

43 2 minimal

23 3 mild

18 4 moderate

5 5 severe

1 6 extreme

---------------------------------------------------------------------------------------------------------------------------------------------------------------------------------------------------------------------------------------------------------------

C1_Q9_B2 (sum) C1_Q9_B2

---------------------------------------------------------------------------------------------------------------------------------------------------------------------------------------------------------------------------------------------------------------

type: numeric (double)

label: CPASS1, but 1 nonmissing value is not labeled

range: [0,6] units: 1

unique values: 7 missing .: 0/302

tabulation: Freq. Numeric Label

1 0

209 1 not at all

41 2 minimal

30 3 mild

13 4 moderate

7 5 severe

1 6 extreme

---------------------------------------------------------------------------------------------------------------------------------------------------------------------------------------------------------------------------------------------------------------

C1_Q9_B3 (sum) C1_Q9_B3

---------------------------------------------------------------------------------------------------------------------------------------------------------------------------------------------------------------------------------------------------------------

type: numeric (double)

label: CPASS1

range: [1,6] units: 1

unique values: 6 missing .: 0/302

tabulation: Freq. Numeric Label

206 1 not at all

39 2 minimal

28 3 mild

17 4 moderate

9 5 severe

3 6 extreme

---------------------------------------------------------------------------------------------------------------------------------------------------------------------------------------------------------------------------------------------------------------

C1_Q9_B4 (sum) C1_Q9_B4

---------------------------------------------------------------------------------------------------------------------------------------------------------------------------------------------------------------------------------------------------------------

type: numeric (double)

label: CPASS1

range: [1,6] units: 1

unique values: 6 missing .: 0/302

tabulation: Freq. Numeric Label

211 1 not at all

38 2 minimal

29 3 mild

15 4 moderate

8 5 severe

1 6 extreme

---------------------------------------------------------------------------------------------------------------------------------------------------------------------------------------------------------------------------------------------------------------

C1_Q9_B5 (sum) C1_Q9_B5

---------------------------------------------------------------------------------------------------------------------------------------------------------------------------------------------------------------------------------------------------------------

type: numeric (double)

label: CPASS1, but 1 nonmissing value is not labeled

range: [0,6] units: 1

unique values: 7 missing .: 0/302

tabulation: Freq. Numeric Label

1 0

203 1 not at all

44 2 minimal

30 3 mild

16 4 moderate

7 5 severe

1 6 extreme

---------------------------------------------------------------------------------------------------------------------------------------------------------------------------------------------------------------------------------------------------------------

C1_Q9_B6 (sum) C1_Q9_B6

---------------------------------------------------------------------------------------------------------------------------------------------------------------------------------------------------------------------------------------------------------------

type: numeric (double)

label: CPASS1

range: [1,6] units: 1

unique values: 6 missing .: 0/302

tabulation: Freq. Numeric Label

211 1 not at all

39 2 minimal

32 3 mild

12 4 moderate

4 5 severe

4 6 extreme

---------------------------------------------------------------------------------------------------------------------------------------------------------------------------------------------------------------------------------------------------------------

C1_Q9_B7 (sum) C1_Q9_B7

---------------------------------------------------------------------------------------------------------------------------------------------------------------------------------------------------------------------------------------------------------------

type: numeric (double)

label: CPASS1, but 1 nonmissing value is not labeled

range: [0,6] units: 1

unique values: 7 missing .: 0/302

tabulation: Freq. Numeric Label

1 0

195 1 not at all

46 2 minimal

34 3 mild

19 4 moderate

4 5 severe

3 6 extreme

---------------------------------------------------------------------------------------------------------------------------------------------------------------------------------------------------------------------------------------------------------------

C1_Q9_A1 (sum) C1_Q9_A1

---------------------------------------------------------------------------------------------------------------------------------------------------------------------------------------------------------------------------------------------------------------

type: numeric (double)

label: CPASS1

range: [1,6] units: 1

unique values: 6 missing .: 0/302

tabulation: Freq. Numeric Label

160 1 not at all

54 2 minimal

44 3 mild

32 4 moderate

9 5 severe

3 6 extreme

---------------------------------------------------------------------------------------------------------------------------------------------------------------------------------------------------------------------------------------------------------------

C1_Q9_A2 (sum) C1_Q9_A2

---------------------------------------------------------------------------------------------------------------------------------------------------------------------------------------------------------------------------------------------------------------

type: numeric (double)

label: CPASS1

range: [1,6] units: 1

unique values: 6 missing .: 0/302

tabulation: Freq. Numeric Label

151 1 not at all

62 2 minimal

49 3 mild

25 4 moderate

12 5 severe

3 6 extreme

---------------------------------------------------------------------------------------------------------------------------------------------------------------------------------------------------------------------------------------------------------------

C1_Q9_A3 (sum) C1_Q9_A3

---------------------------------------------------------------------------------------------------------------------------------------------------------------------------------------------------------------------------------------------------------------

type: numeric (double)

label: CPASS1

range: [1,6] units: 1

unique values: 6 missing .: 0/302

tabulation: Freq. Numeric Label

172 1 not at all

61 2 minimal

39 3 mild

23 4 moderate

5 5 severe

2 6 extreme

---------------------------------------------------------------------------------------------------------------------------------------------------------------------------------------------------------------------------------------------------------------

C1_Q9_A4 (sum) C1_Q9_A4

---------------------------------------------------------------------------------------------------------------------------------------------------------------------------------------------------------------------------------------------------------------

type: numeric (double)

label: CPASS1

range: [1,6] units: 1

unique values: 6 missing .: 0/302

tabulation: Freq. Numeric Label

212 1 not at all

46 2 minimal

27 3 mild

14 4 moderate

2 5 severe

1 6 extreme

---------------------------------------------------------------------------------------------------------------------------------------------------------------------------------------------------------------------------------------------------------------

C1_Q9_A5 (sum) C1_Q9_A5

---------------------------------------------------------------------------------------------------------------------------------------------------------------------------------------------------------------------------------------------------------------

type: numeric (double)

label: CPASS1

range: [1,4] units: 1

unique values: 4 missing .: 0/302

tabulation: Freq. Numeric Label

213 1 not at all

52 2 minimal

22 3 mild

15 4 moderate

---------------------------------------------------------------------------------------------------------------------------------------------------------------------------------------------------------------------------------------------------------------

C1_Q9_A6 (sum) C1_Q9_A6

---------------------------------------------------------------------------------------------------------------------------------------------------------------------------------------------------------------------------------------------------------------

type: numeric (double)

label: CPASS1

range: [1,5] units: 1

unique values: 5 missing .: 0/302

tabulation: Freq. Numeric Label

228 1 not at all

38 2 minimal

29 3 mild

5 4 moderate

2 5 severe

---------------------------------------------------------------------------------------------------------------------------------------------------------------------------------------------------------------------------------------------------------------

C1_Q9_A7 (sum) C1_Q9_A7

---------------------------------------------------------------------------------------------------------------------------------------------------------------------------------------------------------------------------------------------------------------

type: numeric (double)

label: CPASS1

range: [1,5] units: 1

unique values: 5 missing .: 0/302

tabulation: Freq. Numeric Label

241 1 not at all

35 2 minimal

13 3 mild

12 4 moderate

1 5 severe

---------------------------------------------------------------------------------------------------------------------------------------------------------------------------------------------------------------------------------------------------------------

C1_Q9_A8 (sum) C1_Q9_A8

---------------------------------------------------------------------------------------------------------------------------------------------------------------------------------------------------------------------------------------------------------------

type: numeric (double)

label: CPASS1

range: [1,5] units: 1

unique values: 5 missing .: 0/302

tabulation: Freq. Numeric Label

246 1 not at all

35 2 minimal

12 3 mild

7 4 moderate

2 5 severe

---------------------------------------------------------------------------------------------------------------------------------------------------------------------------------------------------------------------------------------------------------------

C1_Q9_A9 (sum) C1_Q9_A9

---------------------------------------------------------------------------------------------------------------------------------------------------------------------------------------------------------------------------------------------------------------

type: numeric (double)

label: CPASS1

range: [1,4] units: 1

unique values: 4 missing .: 0/302

tabulation: Freq. Numeric Label

248 1 not at all

35 2 minimal

10 3 mild

9 4 moderate

---------------------------------------------------------------------------------------------------------------------------------------------------------------------------------------------------------------------------------------------------------------

C1_Q9_A10 (sum) C1_Q9_A10

---------------------------------------------------------------------------------------------------------------------------------------------------------------------------------------------------------------------------------------------------------------

type: numeric (double)

label: CPASS1

range: [1,5] units: 1

unique values: 5 missing .: 0/302

tabulation: Freq. Numeric Label

242 1 not at all

29 2 minimal

21 3 mild

9 4 moderate

1 5 severe

---------------------------------------------------------------------------------------------------------------------------------------------------------------------------------------------------------------------------------------------------------------

C1_Q10_B1 (sum) C1_Q10_B1

---------------------------------------------------------------------------------------------------------------------------------------------------------------------------------------------------------------------------------------------------------------

type: numeric (double)

label: CPASS1, but 1 nonmissing value is not labeled

range: [0,6] units: 1

unique values: 7 missing .: 0/302

tabulation: Freq. Numeric Label

1 0

166 1 not at all

53 2 minimal

48 3 mild

17 4 moderate

16 5 severe

1 6 extreme

---------------------------------------------------------------------------------------------------------------------------------------------------------------------------------------------------------------------------------------------------------------

C1_Q10_B2 (sum) C1_Q10_B2

---------------------------------------------------------------------------------------------------------------------------------------------------------------------------------------------------------------------------------------------------------------

type: numeric (double)

label: CPASS1, but 1 nonmissing value is not labeled

range: [0,6] units: 1

unique values: 7 missing .: 0/302

tabulation: Freq. Numeric Label

1 0

165 1 not at all

62 2 minimal

41 3 mild

21 4 moderate

11 5 severe

1 6 extreme

---------------------------------------------------------------------------------------------------------------------------------------------------------------------------------------------------------------------------------------------------------------

C1_Q10_B3 (sum) C1_Q10_B3

---------------------------------------------------------------------------------------------------------------------------------------------------------------------------------------------------------------------------------------------------------------

type: numeric (double)

label: CPASS1

range: [1,6] units: 1

unique values: 6 missing .: 0/302

tabulation: Freq. Numeric Label

170 1 not at all

53 2 minimal

39 3 mild

26 4 moderate

10 5 severe

4 6 extreme

---------------------------------------------------------------------------------------------------------------------------------------------------------------------------------------------------------------------------------------------------------------

C1_Q10_B4 (sum) C1_Q10_B4

---------------------------------------------------------------------------------------------------------------------------------------------------------------------------------------------------------------------------------------------------------------

type: numeric (double)

label: CPASS1

range: [1,6] units: 1

unique values: 6 missing .: 0/302

tabulation: Freq. Numeric Label

157 1 not at all

59 2 minimal

48 3 mild

26 4 moderate

9 5 severe

3 6 extreme

---------------------------------------------------------------------------------------------------------------------------------------------------------------------------------------------------------------------------------------------------------------

C1_Q10_B5 (sum) C1_Q10_B5

---------------------------------------------------------------------------------------------------------------------------------------------------------------------------------------------------------------------------------------------------------------

type: numeric (double)

label: CPASS1, but 1 nonmissing value is not labeled

range: [0,6] units: 1

unique values: 7 missing .: 0/302

tabulation: Freq. Numeric Label

1 0

160 1 not at all

59 2 minimal

54 3 mild

19 4 moderate

6 5 severe

3 6 extreme

---------------------------------------------------------------------------------------------------------------------------------------------------------------------------------------------------------------------------------------------------------------

C1_Q10_B6 (sum) C1_Q10_B6

---------------------------------------------------------------------------------------------------------------------------------------------------------------------------------------------------------------------------------------------------------------

type: numeric (double)

label: CPASS1

range: [1,6] units: 1

unique values: 6 missing .: 0/302

tabulation: Freq. Numeric Label

162 1 not at all

56 2 minimal

47 3 mild

28 4 moderate

7 5 severe

2 6 extreme

---------------------------------------------------------------------------------------------------------------------------------------------------------------------------------------------------------------------------------------------------------------

C1_Q10_B7 (sum) C1_Q10_B7

---------------------------------------------------------------------------------------------------------------------------------------------------------------------------------------------------------------------------------------------------------------

type: numeric (double)

label: CPASS1, but 1 nonmissing value is not labeled

range: [0,6] units: 1

unique values: 7 missing .: 0/302

tabulation: Freq. Numeric Label

1 0

157 1 not at all

58 2 minimal

46 3 mild

31 4 moderate

6 5 severe

3 6 extreme

---------------------------------------------------------------------------------------------------------------------------------------------------------------------------------------------------------------------------------------------------------------

C1_Q10_A1 (sum) C1_Q10_A1

---------------------------------------------------------------------------------------------------------------------------------------------------------------------------------------------------------------------------------------------------------------

type: numeric (double)

label: CPASS1

range: [1,6] units: 1

unique values: 6 missing .: 0/302

tabulation: Freq. Numeric Label

134 1 not at all

59 2 minimal

58 3 mild

35 4 moderate

12 5 severe

4 6 extreme

---------------------------------------------------------------------------------------------------------------------------------------------------------------------------------------------------------------------------------------------------------------

C1_Q10_A2 (sum) C1_Q10_A2

---------------------------------------------------------------------------------------------------------------------------------------------------------------------------------------------------------------------------------------------------------------

type: numeric (double)

label: CPASS1

range: [1,6] units: 1

unique values: 6 missing .: 0/302

tabulation: Freq. Numeric Label

139 1 not at all

69 2 minimal

48 3 mild

28 4 moderate

13 5 severe

5 6 extreme

---------------------------------------------------------------------------------------------------------------------------------------------------------------------------------------------------------------------------------------------------------------

C1_Q10_A3 (sum) C1_Q10_A3

---------------------------------------------------------------------------------------------------------------------------------------------------------------------------------------------------------------------------------------------------------------

type: numeric (double)

label: CPASS1

range: [1,6] units: 1

unique values: 6 missing .: 0/302

tabulation: Freq. Numeric Label

151 1 not at all

66 2 minimal

46 3 mild

28 4 moderate

8 5 severe

3 6 extreme

---------------------------------------------------------------------------------------------------------------------------------------------------------------------------------------------------------------------------------------------------------------

C1_Q10_A4 (sum) C1_Q10_A4

---------------------------------------------------------------------------------------------------------------------------------------------------------------------------------------------------------------------------------------------------------------

type: numeric (double)

label: CPASS1

range: [1,6] units: 1

unique values: 6 missing .: 0/302

tabulation: Freq. Numeric Label

162 1 not at all

58 2 minimal

48 3 mild

26 4 moderate

5 5 severe

3 6 extreme

---------------------------------------------------------------------------------------------------------------------------------------------------------------------------------------------------------------------------------------------------------------

C1_Q10_A5 (sum) C1_Q10_A5

---------------------------------------------------------------------------------------------------------------------------------------------------------------------------------------------------------------------------------------------------------------

type: numeric (double)

label: CPASS1

range: [1,6] units: 1

unique values: 6 missing .: 0/302

tabulation: Freq. Numeric Label

191 1 not at all

56 2 minimal

32 3 mild

18 4 moderate

4 5 severe

1 6 extreme

---------------------------------------------------------------------------------------------------------------------------------------------------------------------------------------------------------------------------------------------------------------

C1_Q10_A6 (sum) C1_Q10_A6

---------------------------------------------------------------------------------------------------------------------------------------------------------------------------------------------------------------------------------------------------------------

type: numeric (double)

label: CPASS1

range: [1,6] units: 1

unique values: 6 missing .: 0/302

tabulation: Freq. Numeric Label

199 1 not at all

50 2 minimal

30 3 mild

17 4 moderate

5 5 severe

1 6 extreme

---------------------------------------------------------------------------------------------------------------------------------------------------------------------------------------------------------------------------------------------------------------

C1_Q10_A7 (sum) C1_Q10_A7

---------------------------------------------------------------------------------------------------------------------------------------------------------------------------------------------------------------------------------------------------------------

type: numeric (double)

label: CPASS1

range: [1,6] units: 1

unique values: 6 missing .: 0/302

tabulation: Freq. Numeric Label

200 1 not at all

50 2 minimal

31 3 mild

16 4 moderate

4 5 severe

1 6 extreme

---------------------------------------------------------------------------------------------------------------------------------------------------------------------------------------------------------------------------------------------------------------

C1_Q10_A8 (sum) C1_Q10_A8

---------------------------------------------------------------------------------------------------------------------------------------------------------------------------------------------------------------------------------------------------------------

type: numeric (double)

label: CPASS1

range: [1,6] units: 1

unique values: 6 missing .: 0/302

tabulation: Freq. Numeric Label

222 1 not at all

35 2 minimal

26 3 mild

15 4 moderate

3 5 severe

1 6 extreme

---------------------------------------------------------------------------------------------------------------------------------------------------------------------------------------------------------------------------------------------------------------

C1_Q10_A9 (sum) C1_Q10_A9

---------------------------------------------------------------------------------------------------------------------------------------------------------------------------------------------------------------------------------------------------------------

type: numeric (double)

label: CPASS1

range: [1,5] units: 1

unique values: 5 missing .: 0/302

tabulation: Freq. Numeric Label

208 1 not at all

54 2 minimal

20 3 mild

16 4 moderate

4 5 severe

---------------------------------------------------------------------------------------------------------------------------------------------------------------------------------------------------------------------------------------------------------------

C1_Q10_A10 (sum) C1_Q10_A10

---------------------------------------------------------------------------------------------------------------------------------------------------------------------------------------------------------------------------------------------------------------

type: numeric (double)

label: CPASS1

range: [1,6] units: 1

unique values: 6 missing .: 0/302

tabulation: Freq. Numeric Label

209 1 not at all

44 2 minimal

26 3 mild

18 4 moderate

4 5 severe

1 6 extreme

---------------------------------------------------------------------------------------------------------------------------------------------------------------------------------------------------------------------------------------------------------------

C1_Q11_B1 (sum) C1_Q11_B1

---------------------------------------------------------------------------------------------------------------------------------------------------------------------------------------------------------------------------------------------------------------

type: numeric (double)

label: CPASS1, but 1 nonmissing value is not labeled

range: [0,6] units: 1

unique values: 7 missing .: 0/302

tabulation: Freq. Numeric Label

1 0

161 1 not at all

56 2 minimal

37 3 mild

33 4 moderate

11 5 severe

3 6 extreme

---------------------------------------------------------------------------------------------------------------------------------------------------------------------------------------------------------------------------------------------------------------

C1_Q11_B2 (sum) C1_Q11_B2

---------------------------------------------------------------------------------------------------------------------------------------------------------------------------------------------------------------------------------------------------------------

type: numeric (double)

label: CPASS1, but 1 nonmissing value is not labeled

range: [0,6] units: 1

unique values: 7 missing .: 0/302

tabulation: Freq. Numeric Label

1 0

165 1 not at all

54 2 minimal

43 3 mild

24 4 moderate

11 5 severe

4 6 extreme

---------------------------------------------------------------------------------------------------------------------------------------------------------------------------------------------------------------------------------------------------------------

C1_Q11_B3 (sum) C1_Q11_B3

---------------------------------------------------------------------------------------------------------------------------------------------------------------------------------------------------------------------------------------------------------------

type: numeric (double)

label: CPASS1

range: [1,6] units: 1

unique values: 6 missing .: 0/302

tabulation: Freq. Numeric Label

163 1 not at all

63 2 minimal

41 3 mild

22 4 moderate

9 5 severe

4 6 extreme

---------------------------------------------------------------------------------------------------------------------------------------------------------------------------------------------------------------------------------------------------------------

C1_Q11_B4 (sum) C1_Q11_B4

---------------------------------------------------------------------------------------------------------------------------------------------------------------------------------------------------------------------------------------------------------------

type: numeric (double)

label: CPASS1

range: [1,6] units: 1

unique values: 6 missing .: 0/302

tabulation: Freq. Numeric Label

166 1 not at all

56 2 minimal

47 3 mild

22 4 moderate

7 5 severe

4 6 extreme

---------------------------------------------------------------------------------------------------------------------------------------------------------------------------------------------------------------------------------------------------------------

C1_Q11_B5 (sum) C1_Q11_B5

---------------------------------------------------------------------------------------------------------------------------------------------------------------------------------------------------------------------------------------------------------------

type: numeric (double)

label: CPASS1, but 1 nonmissing value is not labeled

range: [0,6] units: 1

unique values: 7 missing .: 0/302

tabulation: Freq. Numeric Label

1 0

158 1 not at all

59 2 minimal

54 3 mild

17 4 moderate

9 5 severe

4 6 extreme

---------------------------------------------------------------------------------------------------------------------------------------------------------------------------------------------------------------------------------------------------------------

C1_Q11_B6 (sum) C1_Q11_B6

---------------------------------------------------------------------------------------------------------------------------------------------------------------------------------------------------------------------------------------------------------------

type: numeric (double)

label: CPASS1

range: [1,6] units: 1

unique values: 6 missing .: 0/302

tabulation: Freq. Numeric Label

155 1 not at all

70 2 minimal

50 3 mild

16 4 moderate

7 5 severe

4 6 extreme

---------------------------------------------------------------------------------------------------------------------------------------------------------------------------------------------------------------------------------------------------------------

C1_Q11_B7 (sum) C1_Q11_B7

---------------------------------------------------------------------------------------------------------------------------------------------------------------------------------------------------------------------------------------------------------------

type: numeric (double)

label: CPASS1, but 1 nonmissing value is not labeled

range: [0,6] units: 1

unique values: 7 missing .: 0/302

tabulation: Freq. Numeric Label

1 0

144 1 not at all

58 2 minimal

55 3 mild

27 4 moderate

12 5 severe

5 6 extreme

---------------------------------------------------------------------------------------------------------------------------------------------------------------------------------------------------------------------------------------------------------------

C1_Q11_A1 (sum) C1_Q11_A1

---------------------------------------------------------------------------------------------------------------------------------------------------------------------------------------------------------------------------------------------------------------

type: numeric (double)

label: CPASS1

range: [1,6] units: 1

unique values: 6 missing .: 0/302

tabulation: Freq. Numeric Label

97 1 not at all

63 2 minimal

67 3 mild

47 4 moderate

20 5 severe

8 6 extreme

---------------------------------------------------------------------------------------------------------------------------------------------------------------------------------------------------------------------------------------------------------------

C1_Q11_A2 (sum) C1_Q11_A2

---------------------------------------------------------------------------------------------------------------------------------------------------------------------------------------------------------------------------------------------------------------

type: numeric (double)

label: CPASS1

range: [1,6] units: 1

unique values: 6 missing .: 0/302

tabulation: Freq. Numeric Label

94 1 not at all

76 2 minimal

67 3 mild

33 4 moderate

22 5 severe

10 6 extreme

---------------------------------------------------------------------------------------------------------------------------------------------------------------------------------------------------------------------------------------------------------------

C1_Q11_A3 (sum) C1_Q11_A3

---------------------------------------------------------------------------------------------------------------------------------------------------------------------------------------------------------------------------------------------------------------

type: numeric (double)

label: CPASS1

range: [1,6] units: 1

unique values: 6 missing .: 0/302

tabulation: Freq. Numeric Label

129 1 not at all

73 2 minimal

52 3 mild

28 4 moderate

18 5 severe

2 6 extreme

---------------------------------------------------------------------------------------------------------------------------------------------------------------------------------------------------------------------------------------------------------------

C1_Q11_A4 (sum) C1_Q11_A4

---------------------------------------------------------------------------------------------------------------------------------------------------------------------------------------------------------------------------------------------------------------

type: numeric (double)

label: CPASS1

range: [1,6] units: 1

unique values: 6 missing .: 0/302

tabulation: Freq. Numeric Label

165 1 not at all

52 2 minimal

48 3 mild

27 4 moderate

6 5 severe

4 6 extreme

---------------------------------------------------------------------------------------------------------------------------------------------------------------------------------------------------------------------------------------------------------------

C1_Q11_A5 (sum) C1_Q11_A5

---------------------------------------------------------------------------------------------------------------------------------------------------------------------------------------------------------------------------------------------------------------

type: numeric (double)

label: CPASS1

range: [1,6] units: 1

unique values: 6 missing .: 0/302

tabulation: Freq. Numeric Label

180 1 not at all

58 2 minimal

44 3 mild

16 4 moderate

3 5 severe

1 6 extreme

---------------------------------------------------------------------------------------------------------------------------------------------------------------------------------------------------------------------------------------------------------------

C1_Q11_A6 (sum) C1_Q11_A6

---------------------------------------------------------------------------------------------------------------------------------------------------------------------------------------------------------------------------------------------------------------

type: numeric (double)

label: CPASS1

range: [1,6] units: 1

unique values: 6 missing .: 0/302

tabulation: Freq. Numeric Label

196 1 not at all

52 2 minimal

33 3 mild

12 4 moderate

6 5 severe

3 6 extreme

---------------------------------------------------------------------------------------------------------------------------------------------------------------------------------------------------------------------------------------------------------------

C1_Q11_A7 (sum) C1_Q11_A7

---------------------------------------------------------------------------------------------------------------------------------------------------------------------------------------------------------------------------------------------------------------

type: numeric (double)

label: CPASS1

range: [1,6] units: 1

unique values: 6 missing .: 0/302

tabulation: Freq. Numeric Label

198 1 not at all

52 2 minimal

30 3 mild

17 4 moderate

3 5 severe

2 6 extreme

---------------------------------------------------------------------------------------------------------------------------------------------------------------------------------------------------------------------------------------------------------------

C1_Q11_A8 (sum) C1_Q11_A8

---------------------------------------------------------------------------------------------------------------------------------------------------------------------------------------------------------------------------------------------------------------

type: numeric (double)

label: CPASS1

range: [1,5] units: 1

unique values: 5 missing .: 0/302

tabulation: Freq. Numeric Label

215 1 not at all

46 2 minimal

24 3 mild

10 4 moderate

7 5 severe

---------------------------------------------------------------------------------------------------------------------------------------------------------------------------------------------------------------------------------------------------------------

C1_Q11_A9 (sum) C1_Q11_A9

---------------------------------------------------------------------------------------------------------------------------------------------------------------------------------------------------------------------------------------------------------------

type: numeric (double)

label: CPASS1

range: [1,5] units: 1

unique values: 5 missing .: 0/302

tabulation: Freq. Numeric Label

212 1 not at all

51 2 minimal

23 3 mild

13 4 moderate

3 5 severe

---------------------------------------------------------------------------------------------------------------------------------------------------------------------------------------------------------------------------------------------------------------

C1_Q11_A10 (sum) C1_Q11_A10

---------------------------------------------------------------------------------------------------------------------------------------------------------------------------------------------------------------------------------------------------------------

type: numeric (double)

label: CPASS1

range: [1,6] units: 1

unique values: 6 missing .: 0/302

tabulation: Freq. Numeric Label

206 1 not at all

47 2 minimal

28 3 mild

14 4 moderate

6 5 severe

1 6 extreme

---------------------------------------------------------------------------------------------------------------------------------------------------------------------------------------------------------------------------------------------------------------

C1_Q12_B1 (sum) C1_Q12_B1

---------------------------------------------------------------------------------------------------------------------------------------------------------------------------------------------------------------------------------------------------------------

type: numeric (double)

label: CPASS1, but 1 nonmissing value is not labeled

range: [0,6] units: 1

unique values: 7 missing .: 0/302

tabulation: Freq. Numeric Label

1 0

197 1 not at all

34 2 minimal

38 3 mild

25 4 moderate

4 5 severe

3 6 extreme

---------------------------------------------------------------------------------------------------------------------------------------------------------------------------------------------------------------------------------------------------------------

C1_Q12_B2 (sum) C1_Q12_B2

---------------------------------------------------------------------------------------------------------------------------------------------------------------------------------------------------------------------------------------------------------------

type: numeric (double)

label: CPASS1, but 1 nonmissing value is not labeled

range: [0,6] units: 1

unique values: 7 missing .: 0/302

tabulation: Freq. Numeric Label

1 0

203 1 not at all

39 2 minimal

31 3 mild

23 4 moderate

4 5 severe

1 6 extreme

---------------------------------------------------------------------------------------------------------------------------------------------------------------------------------------------------------------------------------------------------------------

C1_Q12_B3 (sum) C1_Q12_B3

---------------------------------------------------------------------------------------------------------------------------------------------------------------------------------------------------------------------------------------------------------------

type: numeric (double)

label: CPASS1

range: [1,6] units: 1

unique values: 6 missing .: 0/302

tabulation: Freq. Numeric Label

198 1 not at all

43 2 minimal

32 3 mild

20 4 moderate

7 5 severe

2 6 extreme

---------------------------------------------------------------------------------------------------------------------------------------------------------------------------------------------------------------------------------------------------------------

C1_Q12_B4 (sum) C1_Q12_B4

---------------------------------------------------------------------------------------------------------------------------------------------------------------------------------------------------------------------------------------------------------------

type: numeric (double)

label: CPASS1

range: [1,6] units: 1

unique values: 6 missing .: 0/302

tabulation: Freq. Numeric Label

205 1 not at all

38 2 minimal

26 3 mild

24 4 moderate

6 5 severe

3 6 extreme

---------------------------------------------------------------------------------------------------------------------------------------------------------------------------------------------------------------------------------------------------------------

C1_Q12_B5 (sum) C1_Q12_B5

---------------------------------------------------------------------------------------------------------------------------------------------------------------------------------------------------------------------------------------------------------------

type: numeric (double)

label: CPASS1, but 2 nonmissing values are not labeled

range: [0,11] units: 1

unique values: 8 missing .: 0/302

tabulation: Freq. Numeric Label

1 0

195 1 not at all

40 2 minimal

35 3 mild

22 4 moderate

5 5 severe

3 6 extreme

1 11

---------------------------------------------------------------------------------------------------------------------------------------------------------------------------------------------------------------------------------------------------------------

C1_Q12_B6 (sum) C1_Q12_B6

---------------------------------------------------------------------------------------------------------------------------------------------------------------------------------------------------------------------------------------------------------------

type: numeric (double)

label: CPASS1

range: [1,6] units: 1

unique values: 6 missing .: 0/302

tabulation: Freq. Numeric Label

190 1 not at all

43 2 minimal

34 3 mild

28 4 moderate

5 5 severe

2 6 extreme

---------------------------------------------------------------------------------------------------------------------------------------------------------------------------------------------------------------------------------------------------------------

C1_Q12_B7 (sum) C1_Q12_B7

---------------------------------------------------------------------------------------------------------------------------------------------------------------------------------------------------------------------------------------------------------------

type: numeric (double)

label: CPASS1, but 1 nonmissing value is not labeled

range: [0,6] units: 1

unique values: 7 missing .: 0/302

tabulation: Freq. Numeric Label

1 0

189 1 not at all

49 2 minimal

31 3 mild

24 4 moderate

6 5 severe

2 6 extreme

---------------------------------------------------------------------------------------------------------------------------------------------------------------------------------------------------------------------------------------------------------------

C1_Q12_A1 (sum) C1_Q12_A1

---------------------------------------------------------------------------------------------------------------------------------------------------------------------------------------------------------------------------------------------------------------

type: numeric (double)

label: CPASS1

range: [1,6] units: 1

unique values: 6 missing .: 0/302

tabulation: Freq. Numeric Label

190 1 not at all

42 2 minimal

33 3 mild

23 4 moderate

11 5 severe

3 6 extreme

---------------------------------------------------------------------------------------------------------------------------------------------------------------------------------------------------------------------------------------------------------------

C1_Q12_A2 (sum) C1_Q12_A2

---------------------------------------------------------------------------------------------------------------------------------------------------------------------------------------------------------------------------------------------------------------

type: numeric (double)

label: CPASS1

range: [1,6] units: 1

unique values: 6 missing .: 0/302

tabulation: Freq. Numeric Label

191 1 not at all

49 2 minimal

33 3 mild

19 4 moderate

6 5 severe

4 6 extreme

---------------------------------------------------------------------------------------------------------------------------------------------------------------------------------------------------------------------------------------------------------------

C1_Q12_A3 (sum) C1_Q12_A3

---------------------------------------------------------------------------------------------------------------------------------------------------------------------------------------------------------------------------------------------------------------

type: numeric (double)

label: CPASS1

range: [1,6] units: 1

unique values: 6 missing .: 0/302

tabulation: Freq. Numeric Label

202 1 not at all

39 2 minimal

36 3 mild

17 4 moderate

7 5 severe

1 6 extreme

---------------------------------------------------------------------------------------------------------------------------------------------------------------------------------------------------------------------------------------------------------------

C1_Q12_A4 (sum) C1_Q12_A4

---------------------------------------------------------------------------------------------------------------------------------------------------------------------------------------------------------------------------------------------------------------

type: numeric (double)

label: CPASS1

range: [1,6] units: 1

unique values: 6 missing .: 0/302

tabulation: Freq. Numeric Label

216 1 not at all

43 2 minimal

25 3 mild

14 4 moderate

3 5 severe

1 6 extreme

---------------------------------------------------------------------------------------------------------------------------------------------------------------------------------------------------------------------------------------------------------------

C1_Q12_A5 (sum) C1_Q12_A5

---------------------------------------------------------------------------------------------------------------------------------------------------------------------------------------------------------------------------------------------------------------

type: numeric (double)

label: CPASS1

range: [1,5] units: 1

unique values: 5 missing .: 0/302

tabulation: Freq. Numeric Label

234 1 not at all

31 2 minimal

27 3 mild

7 4 moderate

3 5 severe

---------------------------------------------------------------------------------------------------------------------------------------------------------------------------------------------------------------------------------------------------------------

C1_Q12_A6 (sum) C1_Q12_A6

---------------------------------------------------------------------------------------------------------------------------------------------------------------------------------------------------------------------------------------------------------------

type: numeric (double)

label: CPASS1

range: [1,5] units: 1

unique values: 5 missing .: 0/302

tabulation: Freq. Numeric Label

245 1 not at all

34 2 minimal

11 3 mild

10 4 moderate

2 5 severe

---------------------------------------------------------------------------------------------------------------------------------------------------------------------------------------------------------------------------------------------------------------

C1_Q12_A7 (sum) C1_Q12_A7

---------------------------------------------------------------------------------------------------------------------------------------------------------------------------------------------------------------------------------------------------------------

type: numeric (double)

label: CPASS1

range: [1,5] units: 1

unique values: 5 missing .: 0/302

tabulation: Freq. Numeric Label

250 1 not at all

27 2 minimal

11 3 mild

12 4 moderate

2 5 severe

---------------------------------------------------------------------------------------------------------------------------------------------------------------------------------------------------------------------------------------------------------------

C1_Q12_A8 (sum) C1_Q12_A8

---------------------------------------------------------------------------------------------------------------------------------------------------------------------------------------------------------------------------------------------------------------

type: numeric (double)

label: CPASS1

range: [1,6] units: 1

unique values: 5 missing .: 0/302

tabulation: Freq. Numeric Label

266 1 not at all

19 2 minimal

5 3 mild

11 4 moderate

1 6 extreme

---------------------------------------------------------------------------------------------------------------------------------------------------------------------------------------------------------------------------------------------------------------

C1_Q12_A9 (sum) C1_Q12_A9

---------------------------------------------------------------------------------------------------------------------------------------------------------------------------------------------------------------------------------------------------------------

type: numeric (double)

label: CPASS1

range: [1,4] units: 1

unique values: 4 missing .: 0/302

tabulation: Freq. Numeric Label

264 1 not at all

22 2 minimal

10 3 mild

6 4 moderate

---------------------------------------------------------------------------------------------------------------------------------------------------------------------------------------------------------------------------------------------------------------

C1_Q12_A10 (sum) C1_Q12_A10

---------------------------------------------------------------------------------------------------------------------------------------------------------------------------------------------------------------------------------------------------------------

type: numeric (double)

label: CPASS1

range: [1,5] units: 1

unique values: 5 missing .: 0/302

tabulation: Freq. Numeric Label

262 1 not at all

22 2 minimal

7 3 mild

10 4 moderate

1 5 severe

---------------------------------------------------------------------------------------------------------------------------------------------------------------------------------------------------------------------------------------------------------------

C1_Q13_B1 (sum) C1_Q13_B1

---------------------------------------------------------------------------------------------------------------------------------------------------------------------------------------------------------------------------------------------------------------

type: numeric (double)

label: CPASS1, but 1 nonmissing value is not labeled

range: [0,6] units: 1

unique values: 7 missing .: 0/302

tabulation: Freq. Numeric Label

1 0

211 1 not at all

30 2 minimal

38 3 mild

18 4 moderate

3 5 severe

1 6 extreme

---------------------------------------------------------------------------------------------------------------------------------------------------------------------------------------------------------------------------------------------------------------

C1_Q13_B2 (sum) C1_Q13_B2

---------------------------------------------------------------------------------------------------------------------------------------------------------------------------------------------------------------------------------------------------------------

type: numeric (double)

label: CPASS1, but 1 nonmissing value is not labeled

range: [0,5] units: 1

unique values: 6 missing .: 0/302

tabulation: Freq. Numeric Label

1 0

222 1 not at all

29 2 minimal

30 3 mild

17 4 moderate

3 5 severe

---------------------------------------------------------------------------------------------------------------------------------------------------------------------------------------------------------------------------------------------------------------

C1_Q13_B3 (sum) C1_Q13_B3

---------------------------------------------------------------------------------------------------------------------------------------------------------------------------------------------------------------------------------------------------------------

type: numeric (double)

label: CPASS1

range: [1,6] units: 1

unique values: 6 missing .: 0/302

tabulation: Freq. Numeric Label

212 1 not at all

34 2 minimal

31 3 mild

18 4 moderate

6 5 severe

1 6 extreme

---------------------------------------------------------------------------------------------------------------------------------------------------------------------------------------------------------------------------------------------------------------

C1_Q13_B4 (sum) C1_Q13_B4

---------------------------------------------------------------------------------------------------------------------------------------------------------------------------------------------------------------------------------------------------------------

type: numeric (double)

label: CPASS1

range: [1,6] units: 1

unique values: 6 missing .: 0/302

tabulation: Freq. Numeric Label

212 1 not at all

36 2 minimal

29 3 mild

19 4 moderate

4 5 severe

2 6 extreme

---------------------------------------------------------------------------------------------------------------------------------------------------------------------------------------------------------------------------------------------------------------

C1_Q13_B5 (sum) C1_Q13_B5

---------------------------------------------------------------------------------------------------------------------------------------------------------------------------------------------------------------------------------------------------------------

type: numeric (double)

label: CPASS1, but 1 nonmissing value is not labeled

range: [0,6] units: 1

unique values: 7 missing .: 0/302

tabulation: Freq. Numeric Label

1 0

203 1 not at all

43 2 minimal

25 3 mild

23 4 moderate

6 5 severe

1 6 extreme

---------------------------------------------------------------------------------------------------------------------------------------------------------------------------------------------------------------------------------------------------------------

C1_Q13_B6 (sum) C1_Q13_B6

---------------------------------------------------------------------------------------------------------------------------------------------------------------------------------------------------------------------------------------------------------------

type: numeric (double)

label: CPASS1

range: [1,6] units: 1

unique values: 6 missing .: 0/302

tabulation: Freq. Numeric Label

202 1 not at all

41 2 minimal

37 3 mild

14 4 moderate

6 5 severe

2 6 extreme

---------------------------------------------------------------------------------------------------------------------------------------------------------------------------------------------------------------------------------------------------------------

C1_Q13_B7 (sum) C1_Q13_B7

---------------------------------------------------------------------------------------------------------------------------------------------------------------------------------------------------------------------------------------------------------------

type: numeric (double)

label: CPASS1, but 1 nonmissing value is not labeled

range: [0,6] units: 1

unique values: 7 missing .: 0/302

tabulation: Freq. Numeric Label

1 0

208 1 not at all

32 2 minimal

29 3 mild

22 4 moderate

8 5 severe

2 6 extreme

---------------------------------------------------------------------------------------------------------------------------------------------------------------------------------------------------------------------------------------------------------------

C1_Q13_A1 (sum) C1_Q13_A1

---------------------------------------------------------------------------------------------------------------------------------------------------------------------------------------------------------------------------------------------------------------

type: numeric (double)

label: CPASS1

range: [1,6] units: 1

unique values: 6 missing .: 0/302

tabulation: Freq. Numeric Label

201 1 not at all

39 2 minimal

27 3 mild

22 4 moderate

9 5 severe

4 6 extreme

---------------------------------------------------------------------------------------------------------------------------------------------------------------------------------------------------------------------------------------------------------------

C1_Q13_A2 (sum) C1_Q13_A2

---------------------------------------------------------------------------------------------------------------------------------------------------------------------------------------------------------------------------------------------------------------

type: numeric (double)

label: CPASS1

range: [1,6] units: 1

unique values: 6 missing .: 0/302

tabulation: Freq. Numeric Label

208 1 not at all

33 2 minimal

28 3 mild

21 4 moderate

8 5 severe

4 6 extreme

---------------------------------------------------------------------------------------------------------------------------------------------------------------------------------------------------------------------------------------------------------------

C1_Q13_A3 (sum) C1_Q13_A3

---------------------------------------------------------------------------------------------------------------------------------------------------------------------------------------------------------------------------------------------------------------

type: numeric (double)

label: CPASS1

range: [1,6] units: 1

unique values: 6 missing .: 0/302

tabulation: Freq. Numeric Label

218 1 not at all

36 2 minimal

26 3 mild

17 4 moderate

4 5 severe

1 6 extreme

---------------------------------------------------------------------------------------------------------------------------------------------------------------------------------------------------------------------------------------------------------------

C1_Q13_A4 (sum) C1_Q13_A4

---------------------------------------------------------------------------------------------------------------------------------------------------------------------------------------------------------------------------------------------------------------

type: numeric (double)

label: CPASS1

range: [1,5] units: 1

unique values: 5 missing .: 0/302

tabulation: Freq. Numeric Label

230 1 not at all

30 2 minimal

29 3 mild

12 4 moderate

1 5 severe

---------------------------------------------------------------------------------------------------------------------------------------------------------------------------------------------------------------------------------------------------------------

C1_Q13_A5 (sum) C1_Q13_A5

---------------------------------------------------------------------------------------------------------------------------------------------------------------------------------------------------------------------------------------------------------------

type: numeric (double)

label: CPASS1

range: [1,6] units: 1

unique values: 6 missing .: 0/302

tabulation: Freq. Numeric Label

237 1 not at all

29 2 minimal

24 3 mild

10 4 moderate

1 5 severe

1 6 extreme

---------------------------------------------------------------------------------------------------------------------------------------------------------------------------------------------------------------------------------------------------------------

C1_Q13_A6 (sum) C1_Q13_A6

---------------------------------------------------------------------------------------------------------------------------------------------------------------------------------------------------------------------------------------------------------------

type: numeric (double)

label: CPASS1

range: [1,6] units: 1

unique values: 5 missing .: 0/302

tabulation: Freq. Numeric Label

247 1 not at all

32 2 minimal

14 3 mild

8 4 moderate

1 6 extreme

---------------------------------------------------------------------------------------------------------------------------------------------------------------------------------------------------------------------------------------------------------------

C1_Q13_A7 (sum) C1_Q13_A7

---------------------------------------------------------------------------------------------------------------------------------------------------------------------------------------------------------------------------------------------------------------

type: numeric (double)

label: CPASS1

range: [1,5] units: 1

unique values: 5 missing .: 0/302

tabulation: Freq. Numeric Label

253 1 not at all

27 2 minimal

10 3 mild

10 4 moderate

2 5 severe

---------------------------------------------------------------------------------------------------------------------------------------------------------------------------------------------------------------------------------------------------------------

C1_Q13_A8 (sum) C1_Q13_A8

---------------------------------------------------------------------------------------------------------------------------------------------------------------------------------------------------------------------------------------------------------------

type: numeric (double)

label: CPASS1

range: [1,6] units: 1

unique values: 6 missing .: 0/302

tabulation: Freq. Numeric Label

256 1 not at all

30 2 minimal

7 3 mild

6 4 moderate

1 5 severe

2 6 extreme

---------------------------------------------------------------------------------------------------------------------------------------------------------------------------------------------------------------------------------------------------------------

C1_Q13_A9 (sum) C1_Q13_A9

---------------------------------------------------------------------------------------------------------------------------------------------------------------------------------------------------------------------------------------------------------------

type: numeric (double)

label: CPASS1

range: [1,4] units: 1

unique values: 4 missing .: 0/302

tabulation: Freq. Numeric Label

256 1 not at all

23 2 minimal

16 3 mild

7 4 moderate

---------------------------------------------------------------------------------------------------------------------------------------------------------------------------------------------------------------------------------------------------------------

C1_Q13_A10 (sum) C1_Q13_A10

---------------------------------------------------------------------------------------------------------------------------------------------------------------------------------------------------------------------------------------------------------------

type: numeric (double)

label: CPASS1

range: [1,6] units: 1

unique values: 5 missing .: 0/302

tabulation: Freq. Numeric Label

259 1 not at all

22 2 minimal

12 3 mild

8 4 moderate

1 6 extreme

---------------------------------------------------------------------------------------------------------------------------------------------------------------------------------------------------------------------------------------------------------------

C1_Q14_B1 (sum) C1_Q14_B1

---------------------------------------------------------------------------------------------------------------------------------------------------------------------------------------------------------------------------------------------------------------

type: numeric (double)

label: CPASS1, but 1 nonmissing value is not labeled

range: [0,6] units: 1

unique values: 7 missing .: 0/302

tabulation: Freq. Numeric Label

1 0

175 1 not at all

47 2 minimal

34 3 mild

28 4 moderate

14 5 severe

3 6 extreme

---------------------------------------------------------------------------------------------------------------------------------------------------------------------------------------------------------------------------------------------------------------

C1_Q14_B2 (sum) C1_Q14_B2

---------------------------------------------------------------------------------------------------------------------------------------------------------------------------------------------------------------------------------------------------------------

type: numeric (double)

label: CPASS1, but 1 nonmissing value is not labeled

range: [0,6] units: 1

unique values: 7 missing .: 0/302

tabulation: Freq. Numeric Label

1 0

171 1 not at all

42 2 minimal

43 3 mild

31 4 moderate

11 5 severe

3 6 extreme

---------------------------------------------------------------------------------------------------------------------------------------------------------------------------------------------------------------------------------------------------------------

C1_Q14_B3 (sum) C1_Q14_B3

---------------------------------------------------------------------------------------------------------------------------------------------------------------------------------------------------------------------------------------------------------------

type: numeric (double)

label: CPASS1

range: [1,6] units: 1

unique values: 6 missing .: 0/302

tabulation: Freq. Numeric Label

162 1 not at all

45 2 minimal

52 3 mild

29 4 moderate

9 5 severe

5 6 extreme

---------------------------------------------------------------------------------------------------------------------------------------------------------------------------------------------------------------------------------------------------------------

C1_Q14_B4 (sum) C1_Q14_B4

---------------------------------------------------------------------------------------------------------------------------------------------------------------------------------------------------------------------------------------------------------------

type: numeric (double)

label: CPASS1

range: [1,6] units: 1

unique values: 6 missing .: 0/302

tabulation: Freq. Numeric Label

159 1 not at all

54 2 minimal

43 3 mild

33 4 moderate

9 5 severe

4 6 extreme

---------------------------------------------------------------------------------------------------------------------------------------------------------------------------------------------------------------------------------------------------------------

C1_Q14_B5 (sum) C1_Q14_B5

---------------------------------------------------------------------------------------------------------------------------------------------------------------------------------------------------------------------------------------------------------------

type: numeric (double)

label: CPASS1, but 1 nonmissing value is not labeled

range: [0,6] units: 1

unique values: 7 missing .: 0/302

tabulation: Freq. Numeric Label

1 0

166 1 not at all

41 2 minimal

49 3 mild

30 4 moderate

12 5 severe

3 6 extreme

---------------------------------------------------------------------------------------------------------------------------------------------------------------------------------------------------------------------------------------------------------------

C1_Q14_B6 (sum) C1_Q14_B6

---------------------------------------------------------------------------------------------------------------------------------------------------------------------------------------------------------------------------------------------------------------

type: numeric (double)

label: CPASS1

range: [1,6] units: 1

unique values: 6 missing .: 0/302

tabulation: Freq. Numeric Label

165 1 not at all

54 2 minimal

41 3 mild

26 4 moderate

11 5 severe

5 6 extreme

---------------------------------------------------------------------------------------------------------------------------------------------------------------------------------------------------------------------------------------------------------------

C1_Q14_B7 (sum) C1_Q14_B7

---------------------------------------------------------------------------------------------------------------------------------------------------------------------------------------------------------------------------------------------------------------

type: numeric (double)

label: CPASS1, but 1 nonmissing value is not labeled

range: [0,6] units: 1

unique values: 7 missing .: 0/302

tabulation: Freq. Numeric Label

1 0

152 1 not at all

47 2 minimal

52 3 mild

32 4 moderate

15 5 severe

3 6 extreme

---------------------------------------------------------------------------------------------------------------------------------------------------------------------------------------------------------------------------------------------------------------

C1_Q14_A1 (sum) C1_Q14_A1

---------------------------------------------------------------------------------------------------------------------------------------------------------------------------------------------------------------------------------------------------------------

type: numeric (double)

label: CPASS1

range: [1,6] units: 1

unique values: 6 missing .: 0/302

tabulation: Freq. Numeric Label

128 1 not at all

57 2 minimal

54 3 mild

38 4 moderate

20 5 severe

5 6 extreme

---------------------------------------------------------------------------------------------------------------------------------------------------------------------------------------------------------------------------------------------------------------

C1_Q14_A2 (sum) C1_Q14_A2

---------------------------------------------------------------------------------------------------------------------------------------------------------------------------------------------------------------------------------------------------------------

type: numeric (double)

label: CPASS1

range: [1,6] units: 1

unique values: 6 missing .: 0/302

tabulation: Freq. Numeric Label

127 1 not at all

63 2 minimal

44 3 mild

42 4 moderate

17 5 severe

9 6 extreme

---------------------------------------------------------------------------------------------------------------------------------------------------------------------------------------------------------------------------------------------------------------

C1_Q14_A3 (sum) C1_Q14_A3

---------------------------------------------------------------------------------------------------------------------------------------------------------------------------------------------------------------------------------------------------------------

type: numeric (double)

label: CPASS1

range: [1,6] units: 1

unique values: 6 missing .: 0/302

tabulation: Freq. Numeric Label

141 1 not at all

59 2 minimal

55 3 mild

28 4 moderate

16 5 severe

3 6 extreme

---------------------------------------------------------------------------------------------------------------------------------------------------------------------------------------------------------------------------------------------------------------

C1_Q14_A4 (sum) C1_Q14_A4

---------------------------------------------------------------------------------------------------------------------------------------------------------------------------------------------------------------------------------------------------------------

type: numeric (double)

label: CPASS1

range: [1,6] units: 1

unique values: 6 missing .: 0/302

tabulation: Freq. Numeric Label

160 1 not at all

58 2 minimal

42 3 mild

28 4 moderate

9 5 severe

5 6 extreme

---------------------------------------------------------------------------------------------------------------------------------------------------------------------------------------------------------------------------------------------------------------

C1_Q14_A5 (sum) C1_Q14_A5

---------------------------------------------------------------------------------------------------------------------------------------------------------------------------------------------------------------------------------------------------------------

type: numeric (double)

label: CPASS1

range: [1,6] units: 1

unique values: 6 missing .: 0/302

tabulation: Freq. Numeric Label

184 1 not at all

50 2 minimal

44 3 mild

15 4 moderate

4 5 severe

5 6 extreme

---------------------------------------------------------------------------------------------------------------------------------------------------------------------------------------------------------------------------------------------------------------

C1_Q14_A6 (sum) C1_Q14_A6

---------------------------------------------------------------------------------------------------------------------------------------------------------------------------------------------------------------------------------------------------------------

type: numeric (double)

label: CPASS1

range: [1,6] units: 1

unique values: 6 missing .: 0/302

tabulation: Freq. Numeric Label

192 1 not at all

53 2 minimal

36 3 mild

16 4 moderate

4 5 severe

1 6 extreme

---------------------------------------------------------------------------------------------------------------------------------------------------------------------------------------------------------------------------------------------------------------

C1_Q14_A7 (sum) C1_Q14_A7

---------------------------------------------------------------------------------------------------------------------------------------------------------------------------------------------------------------------------------------------------------------

type: numeric (double)

label: CPASS1

range: [1,6] units: 1

unique values: 6 missing .: 0/302

tabulation: Freq. Numeric Label

197 1 not at all

47 2 minimal

35 3 mild

11 4 moderate

9 5 severe

3 6 extreme

---------------------------------------------------------------------------------------------------------------------------------------------------------------------------------------------------------------------------------------------------------------

C1_Q14_A8 (sum) C1_Q14_A8

---------------------------------------------------------------------------------------------------------------------------------------------------------------------------------------------------------------------------------------------------------------

type: numeric (double)

label: CPASS1

range: [1,6] units: 1

unique values: 6 missing .: 0/302

tabulation: Freq. Numeric Label

220 1 not at all

36 2 minimal

27 3 mild

14 4 moderate

4 5 severe

1 6 extreme

---------------------------------------------------------------------------------------------------------------------------------------------------------------------------------------------------------------------------------------------------------------

C1_Q14_A9 (sum) C1_Q14_A9

---------------------------------------------------------------------------------------------------------------------------------------------------------------------------------------------------------------------------------------------------------------

type: numeric (double)

label: CPASS1

range: [1,6] units: 1

unique values: 6 missing .: 0/302

tabulation: Freq. Numeric Label

220 1 not at all

37 2 minimal

27 3 mild

14 4 moderate

3 5 severe

1 6 extreme

---------------------------------------------------------------------------------------------------------------------------------------------------------------------------------------------------------------------------------------------------------------

C1_Q14_A10 (sum) C1_Q14_A10

---------------------------------------------------------------------------------------------------------------------------------------------------------------------------------------------------------------------------------------------------------------

type: numeric (double)

label: CPASS1

range: [1,5] units: 1

unique values: 5 missing .: 0/302

tabulation: Freq. Numeric Label

223 1 not at all

36 2 minimal

22 3 mild

13 4 moderate

8 5 severe

---------------------------------------------------------------------------------------------------------------------------------------------------------------------------------------------------------------------------------------------------------------

C1_Q15_B1 (sum) C1_Q15_B1

---------------------------------------------------------------------------------------------------------------------------------------------------------------------------------------------------------------------------------------------------------------

type: numeric (double)

label: CPASS1, but 1 nonmissing value is not labeled

range: [0,5] units: 1

unique values: 6 missing .: 0/302

tabulation: Freq. Numeric Label

1 0

227 1 not at all

32 2 minimal

25 3 mild

12 4 moderate

5 5 severe

---------------------------------------------------------------------------------------------------------------------------------------------------------------------------------------------------------------------------------------------------------------

C1_Q15_B2 (sum) C1_Q15_B2

---------------------------------------------------------------------------------------------------------------------------------------------------------------------------------------------------------------------------------------------------------------

type: numeric (double)

label: CPASS1, but 1 nonmissing value is not labeled

range: [0,5] units: 1

unique values: 6 missing .: 0/302

tabulation: Freq. Numeric Label

1 0

225 1 not at all

39 2 minimal

21 3 mild

12 4 moderate

4 5 severe

---------------------------------------------------------------------------------------------------------------------------------------------------------------------------------------------------------------------------------------------------------------

C1_Q15_B3 (sum) C1_Q15_B3

---------------------------------------------------------------------------------------------------------------------------------------------------------------------------------------------------------------------------------------------------------------

type: numeric (double)

label: CPASS1

range: [1,6] units: 1

unique values: 6 missing .: 0/302

tabulation: Freq. Numeric Label

217 1 not at all

44 2 minimal

22 3 mild

16 4 moderate

2 5 severe

1 6 extreme

---------------------------------------------------------------------------------------------------------------------------------------------------------------------------------------------------------------------------------------------------------------

C1_Q15_B4 (sum) C1_Q15_B4

---------------------------------------------------------------------------------------------------------------------------------------------------------------------------------------------------------------------------------------------------------------

type: numeric (double)

label: CPASS1

range: [1,6] units: 1

unique values: 6 missing .: 0/302

tabulation: Freq. Numeric Label

228 1 not at all

32 2 minimal

30 3 mild

9 4 moderate

2 5 severe

1 6 extreme

---------------------------------------------------------------------------------------------------------------------------------------------------------------------------------------------------------------------------------------------------------------

C1_Q15_B5 (sum) C1_Q15_B5

---------------------------------------------------------------------------------------------------------------------------------------------------------------------------------------------------------------------------------------------------------------

type: numeric (double)

label: CPASS1, but 1 nonmissing value is not labeled

range: [0,5] units: 1

unique values: 6 missing .: 0/302

tabulation: Freq. Numeric Label

1 0

227 1 not at all

34 2 minimal

24 3 mild

13 4 moderate

3 5 severe

---------------------------------------------------------------------------------------------------------------------------------------------------------------------------------------------------------------------------------------------------------------

C1_Q15_B6 (sum) C1_Q15_B6

---------------------------------------------------------------------------------------------------------------------------------------------------------------------------------------------------------------------------------------------------------------

type: numeric (double)

label: CPASS1

range: [1,6] units: 1

unique values: 6 missing .: 0/302

tabulation: Freq. Numeric Label

220 1 not at all

32 2 minimal

27 3 mild

16 4 moderate

5 5 severe

2 6 extreme

---------------------------------------------------------------------------------------------------------------------------------------------------------------------------------------------------------------------------------------------------------------

C1_Q15_B7 (sum) C1_Q15_B7

---------------------------------------------------------------------------------------------------------------------------------------------------------------------------------------------------------------------------------------------------------------

type: numeric (double)

label: CPASS1, but 1 nonmissing value is not labeled

range: [0,6] units: 1

unique values: 7 missing .: 0/302

tabulation: Freq. Numeric Label

1 0

213 1 not at all

36 2 minimal

20 3 mild

21 4 moderate

9 5 severe

2 6 extreme

---------------------------------------------------------------------------------------------------------------------------------------------------------------------------------------------------------------------------------------------------------------

C1_Q15_A1 (sum) C1_Q15_A1

---------------------------------------------------------------------------------------------------------------------------------------------------------------------------------------------------------------------------------------------------------------

type: numeric (double)

label: CPASS1

range: [1,6] units: 1

unique values: 6 missing .: 0/302

tabulation: Freq. Numeric Label

204 1 not at all

43 2 minimal

24 3 mild

20 4 moderate

8 5 severe

3 6 extreme

---------------------------------------------------------------------------------------------------------------------------------------------------------------------------------------------------------------------------------------------------------------

C1_Q15_A2 (sum) C1_Q15_A2

---------------------------------------------------------------------------------------------------------------------------------------------------------------------------------------------------------------------------------------------------------------

type: numeric (double)

label: CPASS1

range: [1,6] units: 1

unique values: 6 missing .: 0/302

tabulation: Freq. Numeric Label

200 1 not at all

39 2 minimal

34 3 mild

20 4 moderate

8 5 severe

1 6 extreme

---------------------------------------------------------------------------------------------------------------------------------------------------------------------------------------------------------------------------------------------------------------

C1_Q15_A3 (sum) C1_Q15_A3

---------------------------------------------------------------------------------------------------------------------------------------------------------------------------------------------------------------------------------------------------------------

type: numeric (double)

label: CPASS1

range: [1,5] units: 1

unique values: 5 missing .: 0/302

tabulation: Freq. Numeric Label

208 1 not at all

52 2 minimal

26 3 mild

12 4 moderate

4 5 severe

---------------------------------------------------------------------------------------------------------------------------------------------------------------------------------------------------------------------------------------------------------------

C1_Q15_A4 (sum) C1_Q15_A4

---------------------------------------------------------------------------------------------------------------------------------------------------------------------------------------------------------------------------------------------------------------

type: numeric (double)

label: CPASS1

range: [1,6] units: 1

unique values: 6 missing .: 0/302

tabulation: Freq. Numeric Label

218 1 not at all

45 2 minimal

20 3 mild

12 4 moderate

6 5 severe

1 6 extreme

---------------------------------------------------------------------------------------------------------------------------------------------------------------------------------------------------------------------------------------------------------------

C1_Q15_A5 (sum) C1_Q15_A5

---------------------------------------------------------------------------------------------------------------------------------------------------------------------------------------------------------------------------------------------------------------

type: numeric (double)

label: CPASS1

range: [1,6] units: 1

unique values: 5 missing .: 0/302

tabulation: Freq. Numeric Label

237 1 not at all

39 2 minimal

17 3 mild

8 4 moderate

1 6 extreme

---------------------------------------------------------------------------------------------------------------------------------------------------------------------------------------------------------------------------------------------------------------

C1_Q15_A6 (sum) C1_Q15_A6

---------------------------------------------------------------------------------------------------------------------------------------------------------------------------------------------------------------------------------------------------------------

type: numeric (double)

label: CPASS1

range: [1,5] units: 1

unique values: 5 missing .: 0/302

tabulation: Freq. Numeric Label

235 1 not at all

34 2 minimal

22 3 mild

8 4 moderate

3 5 severe

---------------------------------------------------------------------------------------------------------------------------------------------------------------------------------------------------------------------------------------------------------------

C1_Q15_A7 (sum) C1_Q15_A7

---------------------------------------------------------------------------------------------------------------------------------------------------------------------------------------------------------------------------------------------------------------

type: numeric (double)

label: CPASS1

range: [1,6] units: 1

unique values: 6 missing .: 0/302

tabulation: Freq. Numeric Label

243 1 not at all

34 2 minimal

17 3 mild

6 4 moderate

1 5 severe

1 6 extreme

---------------------------------------------------------------------------------------------------------------------------------------------------------------------------------------------------------------------------------------------------------------

C1_Q15_A8 (sum) C1_Q15_A8

---------------------------------------------------------------------------------------------------------------------------------------------------------------------------------------------------------------------------------------------------------------

type: numeric (double)

label: CPASS1

range: [1,5] units: 1

unique values: 5 missing .: 0/302

tabulation: Freq. Numeric Label

257 1 not at all

23 2 minimal

9 3 mild

9 4 moderate

4 5 severe

---------------------------------------------------------------------------------------------------------------------------------------------------------------------------------------------------------------------------------------------------------------

C1_Q15_A9 (sum) C1_Q15_A9

---------------------------------------------------------------------------------------------------------------------------------------------------------------------------------------------------------------------------------------------------------------

type: numeric (double)

label: CPASS1

range: [1,5] units: 1

unique values: 5 missing .: 0/302

tabulation: Freq. Numeric Label

248 1 not at all

28 2 minimal

13 3 mild

11 4 moderate

2 5 severe

---------------------------------------------------------------------------------------------------------------------------------------------------------------------------------------------------------------------------------------------------------------

C1_Q15_A10 (sum) C1_Q15_A10

---------------------------------------------------------------------------------------------------------------------------------------------------------------------------------------------------------------------------------------------------------------

type: numeric (double)

label: CPASS1

range: [1,5] units: 1

unique values: 5 missing .: 0/302

tabulation: Freq. Numeric Label

244 1 not at all

34 2 minimal

15 3 mild

6 4 moderate

3 5 severe

---------------------------------------------------------------------------------------------------------------------------------------------------------------------------------------------------------------------------------------------------------------

C1_Q16_B1 (sum) C1_Q16_B1

---------------------------------------------------------------------------------------------------------------------------------------------------------------------------------------------------------------------------------------------------------------

type: numeric (double)

label: CPASS1, but 1 nonmissing value is not labeled

range: [0,6] units: 1

unique values: 7 missing .: 0/302

tabulation: Freq. Numeric Label

1 0

204 1 not at all

38 2 minimal

28 3 mild

18 4 moderate

10 5 severe

3 6 extreme

---------------------------------------------------------------------------------------------------------------------------------------------------------------------------------------------------------------------------------------------------------------

C1_Q16_B2 (sum) C1_Q16_B2

---------------------------------------------------------------------------------------------------------------------------------------------------------------------------------------------------------------------------------------------------------------

type: numeric (double)

label: CPASS1, but 1 nonmissing value is not labeled

range: [0,6] units: 1

unique values: 7 missing .: 0/302

tabulation: Freq. Numeric Label

1 0

200 1 not at all

47 2 minimal

30 3 mild

17 4 moderate

6 5 severe

1 6 extreme

---------------------------------------------------------------------------------------------------------------------------------------------------------------------------------------------------------------------------------------------------------------

C1_Q16_B3 (sum) C1_Q16_B3

---------------------------------------------------------------------------------------------------------------------------------------------------------------------------------------------------------------------------------------------------------------

type: numeric (double)

label: CPASS1

range: [1,5] units: 1

unique values: 5 missing .: 0/302

tabulation: Freq. Numeric Label

199 1 not at all

42 2 minimal

33 3 mild

22 4 moderate

6 5 severe

---------------------------------------------------------------------------------------------------------------------------------------------------------------------------------------------------------------------------------------------------------------

C1_Q16_B4 (sum) C1_Q16_B4

---------------------------------------------------------------------------------------------------------------------------------------------------------------------------------------------------------------------------------------------------------------

type: numeric (double)

label: CPASS1

range: [1,6] units: 1

unique values: 6 missing .: 0/302

tabulation: Freq. Numeric Label

194 1 not at all

48 2 minimal

35 3 mild

16 4 moderate

8 5 severe

1 6 extreme

---------------------------------------------------------------------------------------------------------------------------------------------------------------------------------------------------------------------------------------------------------------

C1_Q16_B5 (sum) C1_Q16_B5

---------------------------------------------------------------------------------------------------------------------------------------------------------------------------------------------------------------------------------------------------------------

type: numeric (double)

label: CPASS1, but 1 nonmissing value is not labeled

range: [0,6] units: 1

unique values: 7 missing .: 0/302

tabulation: Freq. Numeric Label

1 0

208 1 not at all

31 2 minimal

38 3 mild

14 4 moderate

9 5 severe

1 6 extreme

---------------------------------------------------------------------------------------------------------------------------------------------------------------------------------------------------------------------------------------------------------------

C1_Q16_B6 (sum) C1_Q16_B6

---------------------------------------------------------------------------------------------------------------------------------------------------------------------------------------------------------------------------------------------------------------

type: numeric (double)

label: CPASS1

range: [1,6] units: 1

unique values: 6 missing .: 0/302

tabulation: Freq. Numeric Label

201 1 not at all

42 2 minimal

33 3 mild

17 4 moderate

7 5 severe

2 6 extreme

---------------------------------------------------------------------------------------------------------------------------------------------------------------------------------------------------------------------------------------------------------------

C1_Q16_B7 (sum) C1_Q16_B7

---------------------------------------------------------------------------------------------------------------------------------------------------------------------------------------------------------------------------------------------------------------

type: numeric (double)

label: CPASS1, but 1 nonmissing value is not labeled

range: [0,6] units: 1

unique values: 7 missing .: 0/302

tabulation: Freq. Numeric Label

1 0

203 1 not at all

40 2 minimal

32 3 mild

20 4 moderate

3 5 severe

3 6 extreme

---------------------------------------------------------------------------------------------------------------------------------------------------------------------------------------------------------------------------------------------------------------

C1_Q16_A1 (sum) C1_Q16_A1

---------------------------------------------------------------------------------------------------------------------------------------------------------------------------------------------------------------------------------------------------------------

type: numeric (double)

label: CPASS1

range: [1,6] units: 1

unique values: 6 missing .: 0/302

tabulation: Freq. Numeric Label

188 1 not at all

47 2 minimal

29 3 mild

25 4 moderate

9 5 severe

4 6 extreme

---------------------------------------------------------------------------------------------------------------------------------------------------------------------------------------------------------------------------------------------------------------

C1_Q16_A2 (sum) C1_Q16_A2

---------------------------------------------------------------------------------------------------------------------------------------------------------------------------------------------------------------------------------------------------------------

type: numeric (double)

label: CPASS1

range: [1,6] units: 1

unique values: 6 missing .: 0/302

tabulation: Freq. Numeric Label

190 1 not at all

44 2 minimal

35 3 mild

20 4 moderate

10 5 severe

3 6 extreme

---------------------------------------------------------------------------------------------------------------------------------------------------------------------------------------------------------------------------------------------------------------

C1_Q16_A3 (sum) C1_Q16_A3

---------------------------------------------------------------------------------------------------------------------------------------------------------------------------------------------------------------------------------------------------------------

type: numeric (double)

label: CPASS1

range: [1,6] units: 1

unique values: 6 missing .: 0/302

tabulation: Freq. Numeric Label

190 1 not at all

45 2 minimal

33 3 mild

24 4 moderate

8 5 severe

2 6 extreme

---------------------------------------------------------------------------------------------------------------------------------------------------------------------------------------------------------------------------------------------------------------

C1_Q16_A4 (sum) C1_Q16_A4

---------------------------------------------------------------------------------------------------------------------------------------------------------------------------------------------------------------------------------------------------------------

type: numeric (double)

label: CPASS1

range: [1,6] units: 1

unique values: 6 missing .: 0/302

tabulation: Freq. Numeric Label

213 1 not at all

43 2 minimal

25 3 mild

14 4 moderate

5 5 severe

2 6 extreme

---------------------------------------------------------------------------------------------------------------------------------------------------------------------------------------------------------------------------------------------------------------

C1_Q16_A5 (sum) C1_Q16_A5

---------------------------------------------------------------------------------------------------------------------------------------------------------------------------------------------------------------------------------------------------------------

type: numeric (double)

label: CPASS1

range: [1,5] units: 1

unique values: 5 missing .: 0/302

tabulation: Freq. Numeric Label

220 1 not at all

43 2 minimal

19 3 mild

18 4 moderate

2 5 severe

---------------------------------------------------------------------------------------------------------------------------------------------------------------------------------------------------------------------------------------------------------------

C1_Q16_A6 (sum) C1_Q16_A6

---------------------------------------------------------------------------------------------------------------------------------------------------------------------------------------------------------------------------------------------------------------

type: numeric (double)

label: CPASS1

range: [1,5] units: 1

unique values: 5 missing .: 0/302

tabulation: Freq. Numeric Label

236 1 not at all

32 2 minimal

20 3 mild

12 4 moderate

2 5 severe

---------------------------------------------------------------------------------------------------------------------------------------------------------------------------------------------------------------------------------------------------------------

C1_Q16_A7 (sum) C1_Q16_A7

---------------------------------------------------------------------------------------------------------------------------------------------------------------------------------------------------------------------------------------------------------------

type: numeric (double)

label: CPASS1

range: [1,6] units: 1

unique values: 6 missing .: 0/302

tabulation: Freq. Numeric Label

230 1 not at all

36 2 minimal

19 3 mild

14 4 moderate

2 5 severe

1 6 extreme

---------------------------------------------------------------------------------------------------------------------------------------------------------------------------------------------------------------------------------------------------------------

C1_Q16_A8 (sum) C1_Q16_A8

---------------------------------------------------------------------------------------------------------------------------------------------------------------------------------------------------------------------------------------------------------------

type: numeric (double)

label: CPASS1

range: [1,6] units: 1

unique values: 6 missing .: 0/302

tabulation: Freq. Numeric Label

237 1 not at all

32 2 minimal

18 3 mild

10 4 moderate

4 5 severe

1 6 extreme

---------------------------------------------------------------------------------------------------------------------------------------------------------------------------------------------------------------------------------------------------------------

C1_Q16_A9 (sum) C1_Q16_A9

---------------------------------------------------------------------------------------------------------------------------------------------------------------------------------------------------------------------------------------------------------------

type: numeric (double)

label: CPASS1

range: [1,5] units: 1

unique values: 5 missing .: 0/302

tabulation: Freq. Numeric Label

242 1 not at all

31 2 minimal

16 3 mild

11 4 moderate

2 5 severe

---------------------------------------------------------------------------------------------------------------------------------------------------------------------------------------------------------------------------------------------------------------

C1_Q16_A10 (sum) C1_Q16_A10

---------------------------------------------------------------------------------------------------------------------------------------------------------------------------------------------------------------------------------------------------------------

type: numeric (double)

label: CPASS1

range: [1,6] units: 1

unique values: 6 missing .: 0/302

tabulation: Freq. Numeric Label

239 1 not at all

29 2 minimal

18 3 mild

10 4 moderate

4 5 severe

2 6 extreme

---------------------------------------------------------------------------------------------------------------------------------------------------------------------------------------------------------------------------------------------------------------

C1_Q17_B1 (sum) C1_Q17_B1

---------------------------------------------------------------------------------------------------------------------------------------------------------------------------------------------------------------------------------------------------------------

type: numeric (double)

label: CPASS1, but 1 nonmissing value is not labeled

range: [0,6] units: 1

unique values: 7 missing .: 0/302

tabulation: Freq. Numeric Label

1 0

209 1 not at all

37 2 minimal

30 3 mild

17 4 moderate

5 5 severe

3 6 extreme

---------------------------------------------------------------------------------------------------------------------------------------------------------------------------------------------------------------------------------------------------------------

C1_Q17_B2 (sum) C1_Q17_B2

---------------------------------------------------------------------------------------------------------------------------------------------------------------------------------------------------------------------------------------------------------------

type: numeric (double)

label: CPASS1, but 1 nonmissing value is not labeled

range: [0,6] units: 1

unique values: 7 missing .: 0/302

tabulation: Freq. Numeric Label

1 0

210 1 not at all

43 2 minimal

22 3 mild

21 4 moderate

4 5 severe

1 6 extreme

---------------------------------------------------------------------------------------------------------------------------------------------------------------------------------------------------------------------------------------------------------------

C1_Q17_B3 (sum) C1_Q17_B3

---------------------------------------------------------------------------------------------------------------------------------------------------------------------------------------------------------------------------------------------------------------

type: numeric (double)

label: CPASS1

range: [1,6] units: 1

unique values: 6 missing .: 0/302

tabulation: Freq. Numeric Label

217 1 not at all

36 2 minimal

30 3 mild

12 4 moderate

5 5 severe

2 6 extreme

---------------------------------------------------------------------------------------------------------------------------------------------------------------------------------------------------------------------------------------------------------------

C1_Q17_B4 (sum) C1_Q17_B4

---------------------------------------------------------------------------------------------------------------------------------------------------------------------------------------------------------------------------------------------------------------

type: numeric (double)

label: CPASS1

range: [1,6] units: 1

unique values: 6 missing .: 0/302

tabulation: Freq. Numeric Label

215 1 not at all

38 2 minimal

29 3 mild

14 4 moderate

5 5 severe

1 6 extreme

---------------------------------------------------------------------------------------------------------------------------------------------------------------------------------------------------------------------------------------------------------------

C1_Q17_B5 (sum) C1_Q17_B5

---------------------------------------------------------------------------------------------------------------------------------------------------------------------------------------------------------------------------------------------------------------

type: numeric (double)

label: CPASS1, but 1 nonmissing value is not labeled

range: [0,6] units: 1

unique values: 7 missing .: 0/302

tabulation: Freq. Numeric Label

1 0

204 1 not at all

38 2 minimal

36 3 mild

19 4 moderate

2 5 severe

2 6 extreme

---------------------------------------------------------------------------------------------------------------------------------------------------------------------------------------------------------------------------------------------------------------

C1_Q17_B6 (sum) C1_Q17_B6

---------------------------------------------------------------------------------------------------------------------------------------------------------------------------------------------------------------------------------------------------------------

type: numeric (double)

label: CPASS1

range: [1,6] units: 1

unique values: 6 missing .: 0/302

tabulation: Freq. Numeric Label

210 1 not at all

39 2 minimal

27 3 mild

17 4 moderate

6 5 severe

3 6 extreme

---------------------------------------------------------------------------------------------------------------------------------------------------------------------------------------------------------------------------------------------------------------

C1_Q17_B7 (sum) C1_Q17_B7

---------------------------------------------------------------------------------------------------------------------------------------------------------------------------------------------------------------------------------------------------------------

type: numeric (double)

label: CPASS1, but 1 nonmissing value is not labeled

range: [0,6] units: 1

unique values: 7 missing .: 0/302

tabulation: Freq. Numeric Label

1 0

213 1 not at all

36 2 minimal

27 3 mild

16 4 moderate

4 5 severe

5 6 extreme

---------------------------------------------------------------------------------------------------------------------------------------------------------------------------------------------------------------------------------------------------------------

C1_Q17_A1 (sum) C1_Q17_A1

---------------------------------------------------------------------------------------------------------------------------------------------------------------------------------------------------------------------------------------------------------------

type: numeric (double)

label: CPASS1

range: [1,6] units: 1

unique values: 6 missing .: 0/302

tabulation: Freq. Numeric Label

172 1 not at all

59 2 minimal

35 3 mild

26 4 moderate

6 5 severe

4 6 extreme

---------------------------------------------------------------------------------------------------------------------------------------------------------------------------------------------------------------------------------------------------------------

C1_Q17_A2 (sum) C1_Q17_A2

---------------------------------------------------------------------------------------------------------------------------------------------------------------------------------------------------------------------------------------------------------------

type: numeric (double)

label: CPASS1

range: [1,6] units: 1

unique values: 6 missing .: 0/302

tabulation: Freq. Numeric Label

187 1 not at all

48 2 minimal

40 3 mild

17 4 moderate

7 5 severe

3 6 extreme

---------------------------------------------------------------------------------------------------------------------------------------------------------------------------------------------------------------------------------------------------------------

C1_Q17_A3 (sum) C1_Q17_A3

---------------------------------------------------------------------------------------------------------------------------------------------------------------------------------------------------------------------------------------------------------------

type: numeric (double)

label: CPASS1

range: [1,6] units: 1

unique values: 6 missing .: 0/302

tabulation: Freq. Numeric Label

191 1 not at all

58 2 minimal

30 3 mild

13 4 moderate

8 5 severe

2 6 extreme

---------------------------------------------------------------------------------------------------------------------------------------------------------------------------------------------------------------------------------------------------------------

C1_Q17_A4 (sum) C1_Q17_A4

---------------------------------------------------------------------------------------------------------------------------------------------------------------------------------------------------------------------------------------------------------------

type: numeric (double)

label: CPASS1

range: [1,6] units: 1

unique values: 6 missing .: 0/302

tabulation: Freq. Numeric Label

214 1 not at all

45 2 minimal

23 3 mild

12 4 moderate

6 5 severe

2 6 extreme

---------------------------------------------------------------------------------------------------------------------------------------------------------------------------------------------------------------------------------------------------------------

C1_Q17_A5 (sum) C1_Q17_A5

---------------------------------------------------------------------------------------------------------------------------------------------------------------------------------------------------------------------------------------------------------------

type: numeric (double)

label: CPASS1

range: [1,5] units: 1

unique values: 5 missing .: 0/302

tabulation: Freq. Numeric Label

223 1 not at all

44 2 minimal

18 3 mild

15 4 moderate

2 5 severe

---------------------------------------------------------------------------------------------------------------------------------------------------------------------------------------------------------------------------------------------------------------

C1_Q17_A6 (sum) C1_Q17_A6

---------------------------------------------------------------------------------------------------------------------------------------------------------------------------------------------------------------------------------------------------------------

type: numeric (double)

label: CPASS1

range: [1,5] units: 1

unique values: 5 missing .: 0/302

tabulation: Freq. Numeric Label

235 1 not at all

41 2 minimal

16 3 mild

8 4 moderate

2 5 severe

---------------------------------------------------------------------------------------------------------------------------------------------------------------------------------------------------------------------------------------------------------------

C1_Q17_A7 (sum) C1_Q17_A7

---------------------------------------------------------------------------------------------------------------------------------------------------------------------------------------------------------------------------------------------------------------

type: numeric (double)

label: CPASS1

range: [1,5] units: 1

unique values: 5 missing .: 0/302

tabulation: Freq. Numeric Label

244 1 not at all

43 2 minimal

9 3 mild

5 4 moderate

1 5 severe

---------------------------------------------------------------------------------------------------------------------------------------------------------------------------------------------------------------------------------------------------------------

C1_Q17_A8 (sum) C1_Q17_A8

---------------------------------------------------------------------------------------------------------------------------------------------------------------------------------------------------------------------------------------------------------------

type: numeric (double)

label: CPASS1

range: [1,6] units: 1

unique values: 6 missing .: 0/302

tabulation: Freq. Numeric Label

248 1 not at all

32 2 minimal

10 3 mild

8 4 moderate

2 5 severe

2 6 extreme

---------------------------------------------------------------------------------------------------------------------------------------------------------------------------------------------------------------------------------------------------------------

C1_Q17_A9 (sum) C1_Q17_A9

---------------------------------------------------------------------------------------------------------------------------------------------------------------------------------------------------------------------------------------------------------------

type: numeric (double)

label: CPASS1

range: [1,4] units: 1

unique values: 4 missing .: 0/302

tabulation: Freq. Numeric Label

251 1 not at all

35 2 minimal

10 3 mild

6 4 moderate

---------------------------------------------------------------------------------------------------------------------------------------------------------------------------------------------------------------------------------------------------------------

C1_Q17_A10 (sum) C1_Q17_A10

---------------------------------------------------------------------------------------------------------------------------------------------------------------------------------------------------------------------------------------------------------------

type: numeric (double)

label: CPASS1

range: [1,6] units: 1

unique values: 6 missing .: 0/302

tabulation: Freq. Numeric Label

248 1 not at all

32 2 minimal

12 3 mild

7 4 moderate

2 5 severe

1 6 extreme

---------------------------------------------------------------------------------------------------------------------------------------------------------------------------------------------------------------------------------------------------------------

C1_Q18_B1 (sum) C1_Q18_B1

---------------------------------------------------------------------------------------------------------------------------------------------------------------------------------------------------------------------------------------------------------------

type: numeric (double)

label: CPASS1, but 1 nonmissing value is not labeled

range: [0,5] units: 1

unique values: 6 missing .: 0/302

tabulation: Freq. Numeric Label

1 0

220 1 not at all

37 2 minimal

26 3 mild

15 4 moderate

3 5 severe

---------------------------------------------------------------------------------------------------------------------------------------------------------------------------------------------------------------------------------------------------------------

C1_Q18_B2 (sum) C1_Q18_B2

---------------------------------------------------------------------------------------------------------------------------------------------------------------------------------------------------------------------------------------------------------------

type: numeric (double)

label: CPASS1, but 1 nonmissing value is not labeled

range: [0,6] units: 1

unique values: 7 missing .: 0/302

tabulation: Freq. Numeric Label

1 0

208 1 not at all

40 2 minimal

32 3 mild

19 4 moderate

1 5 severe

1 6 extreme

---------------------------------------------------------------------------------------------------------------------------------------------------------------------------------------------------------------------------------------------------------------

C1_Q18_B3 (sum) C1_Q18_B3

---------------------------------------------------------------------------------------------------------------------------------------------------------------------------------------------------------------------------------------------------------------

type: numeric (double)

label: CPASS1

range: [1,6] units: 1

unique values: 6 missing .: 0/302

tabulation: Freq. Numeric Label

200 1 not at all

40 2 minimal

32 3 mild

22 4 moderate

7 5 severe

1 6 extreme

---------------------------------------------------------------------------------------------------------------------------------------------------------------------------------------------------------------------------------------------------------------

C1_Q18_B4 (sum) C1_Q18_B4

---------------------------------------------------------------------------------------------------------------------------------------------------------------------------------------------------------------------------------------------------------------

type: numeric (double)

label: CPASS1

range: [1,5] units: 1

unique values: 5 missing .: 0/302

tabulation: Freq. Numeric Label

185 1 not at all

41 2 minimal

43 3 mild

26 4 moderate

7 5 severe

---------------------------------------------------------------------------------------------------------------------------------------------------------------------------------------------------------------------------------------------------------------

C1_Q18_B5 (sum) C1_Q18_B5

---------------------------------------------------------------------------------------------------------------------------------------------------------------------------------------------------------------------------------------------------------------

type: numeric (double)

label: CPASS1, but 1 nonmissing value is not labeled

range: [0,5] units: 1

unique values: 6 missing .: 0/302

tabulation: Freq. Numeric Label

1 0

171 1 not at all

50 2 minimal

44 3 mild

25 4 moderate

11 5 severe

---------------------------------------------------------------------------------------------------------------------------------------------------------------------------------------------------------------------------------------------------------------

C1_Q18_B6 (sum) C1_Q18_B6

---------------------------------------------------------------------------------------------------------------------------------------------------------------------------------------------------------------------------------------------------------------

type: numeric (double)

label: CPASS1

range: [1,6] units: 1

unique values: 6 missing .: 0/302

tabulation: Freq. Numeric Label

172 1 not at all

50 2 minimal

39 3 mild

32 4 moderate

6 5 severe

3 6 extreme

---------------------------------------------------------------------------------------------------------------------------------------------------------------------------------------------------------------------------------------------------------------

C1_Q18_B7 (sum) C1_Q18_B7

---------------------------------------------------------------------------------------------------------------------------------------------------------------------------------------------------------------------------------------------------------------

type: numeric (double)

label: CPASS1, but 1 nonmissing value is not labeled

range: [0,6] units: 1

unique values: 7 missing .: 0/302

tabulation: Freq. Numeric Label

1 0

153 1 not at all

53 2 minimal

59 3 mild

22 4 moderate

11 5 severe

3 6 extreme

---------------------------------------------------------------------------------------------------------------------------------------------------------------------------------------------------------------------------------------------------------------

C1_Q18_A1 (sum) C1_Q18_A1

---------------------------------------------------------------------------------------------------------------------------------------------------------------------------------------------------------------------------------------------------------------

type: numeric (double)

label: CPASS1

range: [1,6] units: 1

unique values: 6 missing .: 0/302

tabulation: Freq. Numeric Label

142 1 not at all

75 2 minimal

54 3 mild

20 4 moderate

5 5 severe

6 6 extreme

---------------------------------------------------------------------------------------------------------------------------------------------------------------------------------------------------------------------------------------------------------------

C1_Q18_A2 (sum) C1_Q18_A2

---------------------------------------------------------------------------------------------------------------------------------------------------------------------------------------------------------------------------------------------------------------

type: numeric (double)

label: CPASS1

range: [1,6] units: 1

unique values: 6 missing .: 0/302

tabulation: Freq. Numeric Label

174 1 not at all

56 2 minimal

52 3 mild

13 4 moderate

5 5 severe

2 6 extreme

---------------------------------------------------------------------------------------------------------------------------------------------------------------------------------------------------------------------------------------------------------------

C1_Q18_A3 (sum) C1_Q18_A3

---------------------------------------------------------------------------------------------------------------------------------------------------------------------------------------------------------------------------------------------------------------

type: numeric (double)

label: CPASS1

range: [1,6] units: 1

unique values: 6 missing .: 0/302

tabulation: Freq. Numeric Label

206 1 not at all

51 2 minimal

33 3 mild

9 4 moderate

2 5 severe

1 6 extreme

---------------------------------------------------------------------------------------------------------------------------------------------------------------------------------------------------------------------------------------------------------------

C1_Q18_A4 (sum) C1_Q18_A4

---------------------------------------------------------------------------------------------------------------------------------------------------------------------------------------------------------------------------------------------------------------

type: numeric (double)

label: CPASS1

range: [1,4] units: 1

unique values: 4 missing .: 0/302

tabulation: Freq. Numeric Label

233 1 not at all

43 2 minimal

21 3 mild

5 4 moderate

---------------------------------------------------------------------------------------------------------------------------------------------------------------------------------------------------------------------------------------------------------------

C1_Q18_A5 (sum) C1_Q18_A5

---------------------------------------------------------------------------------------------------------------------------------------------------------------------------------------------------------------------------------------------------------------

type: numeric (double)

label: CPASS1

range: [1,5] units: 1

unique values: 5 missing .: 0/302

tabulation: Freq. Numeric Label

258 1 not at all

31 2 minimal

8 3 mild

4 4 moderate

1 5 severe

---------------------------------------------------------------------------------------------------------------------------------------------------------------------------------------------------------------------------------------------------------------

C1_Q18_A6 (sum) C1_Q18_A6

---------------------------------------------------------------------------------------------------------------------------------------------------------------------------------------------------------------------------------------------------------------

type: numeric (double)

label: CPASS1

range: [1,6] units: 1

unique values: 4 missing .: 0/302

tabulation: Freq. Numeric Label

276 1 not at all

18 2 minimal

7 3 mild

1 6 extreme

---------------------------------------------------------------------------------------------------------------------------------------------------------------------------------------------------------------------------------------------------------------

C1_Q18_A7 (sum) C1_Q18_A7

---------------------------------------------------------------------------------------------------------------------------------------------------------------------------------------------------------------------------------------------------------------

type: numeric (double)

label: CPASS1

range: [1,4] units: 1

unique values: 4 missing .: 0/302

tabulation: Freq. Numeric Label

285 1 not at all

10 2 minimal

5 3 mild

2 4 moderate

---------------------------------------------------------------------------------------------------------------------------------------------------------------------------------------------------------------------------------------------------------------

C1_Q18_A8 (sum) C1_Q18_A8

---------------------------------------------------------------------------------------------------------------------------------------------------------------------------------------------------------------------------------------------------------------

type: numeric (double)

label: CPASS1

range: [1,5] units: 1

unique values: 5 missing .: 0/302

tabulation: Freq. Numeric Label

287 1 not at all

10 2 minimal

3 3 mild

1 4 moderate

1 5 severe

---------------------------------------------------------------------------------------------------------------------------------------------------------------------------------------------------------------------------------------------------------------

C1_Q18_A9 (sum) C1_Q18_A9

---------------------------------------------------------------------------------------------------------------------------------------------------------------------------------------------------------------------------------------------------------------

type: numeric (double)

label: CPASS1

range: [1,4] units: 1

unique values: 4 missing .: 0/302

tabulation: Freq. Numeric Label

288 1 not at all

11 2 minimal

2 3 mild

1 4 moderate

---------------------------------------------------------------------------------------------------------------------------------------------------------------------------------------------------------------------------------------------------------------

C1_Q18_A10 (sum) C1_Q18_A10

---------------------------------------------------------------------------------------------------------------------------------------------------------------------------------------------------------------------------------------------------------------

type: numeric (double)

label: CPASS1

range: [1,4] units: 1

unique values: 4 missing .: 0/302

tabulation: Freq. Numeric Label

288 1 not at all

9 2 minimal

4 3 mild

1 4 moderate

---------------------------------------------------------------------------------------------------------------------------------------------------------------------------------------------------------------------------------------------------------------

C1_Q19_B1 (sum) C1_Q19_B1

---------------------------------------------------------------------------------------------------------------------------------------------------------------------------------------------------------------------------------------------------------------

type: numeric (double)

label: CPASS1, but 1 nonmissing value is not labeled

range: [0,6] units: 1

unique values: 7 missing .: 0/302

tabulation: Freq. Numeric Label

1 0

234 1 not at all

28 2 minimal

22 3 mild

13 4 moderate

3 5 severe

1 6 extreme

---------------------------------------------------------------------------------------------------------------------------------------------------------------------------------------------------------------------------------------------------------------

C1_Q19_B2 (sum) C1_Q19_B2

---------------------------------------------------------------------------------------------------------------------------------------------------------------------------------------------------------------------------------------------------------------

type: numeric (double)

label: CPASS1, but 1 nonmissing value is not labeled

range: [0,6] units: 1

unique values: 7 missing .: 0/302

tabulation: Freq. Numeric Label

1 0

214 1 not at all

45 2 minimal

30 3 mild

7 4 moderate

4 5 severe

1 6 extreme

---------------------------------------------------------------------------------------------------------------------------------------------------------------------------------------------------------------------------------------------------------------

C1_Q19_B3 (sum) C1_Q19_B3

---------------------------------------------------------------------------------------------------------------------------------------------------------------------------------------------------------------------------------------------------------------

type: numeric (double)

label: CPASS1

range: [1,6] units: 1

unique values: 6 missing .: 0/302

tabulation: Freq. Numeric Label

209 1 not at all

43 2 minimal

28 3 mild

15 4 moderate

6 5 severe

1 6 extreme

---------------------------------------------------------------------------------------------------------------------------------------------------------------------------------------------------------------------------------------------------------------

C1_Q19_B4 (sum) C1_Q19_B4

---------------------------------------------------------------------------------------------------------------------------------------------------------------------------------------------------------------------------------------------------------------

type: numeric (double)

label: CPASS1

range: [1,5] units: 1

unique values: 5 missing .: 0/302

tabulation: Freq. Numeric Label

201 1 not at all

49 2 minimal

30 3 mild

15 4 moderate

7 5 severe

---------------------------------------------------------------------------------------------------------------------------------------------------------------------------------------------------------------------------------------------------------------

C1_Q19_B5 (sum) C1_Q19_B5

---------------------------------------------------------------------------------------------------------------------------------------------------------------------------------------------------------------------------------------------------------------

type: numeric (double)

label: CPASS1, but 1 nonmissing value is not labeled

range: [0,5] units: 1

unique values: 6 missing .: 0/302

tabulation: Freq. Numeric Label

1 0

183 1 not at all

45 2 minimal

48 3 mild

17 4 moderate

8 5 severe

---------------------------------------------------------------------------------------------------------------------------------------------------------------------------------------------------------------------------------------------------------------

C1_Q19_B6 (sum) C1_Q19_B6

---------------------------------------------------------------------------------------------------------------------------------------------------------------------------------------------------------------------------------------------------------------

type: numeric (double)

label: CPASS1

range: [1,6] units: 1

unique values: 6 missing .: 0/302

tabulation: Freq. Numeric Label

186 1 not at all

53 2 minimal

34 3 mild

23 4 moderate

3 5 severe

3 6 extreme

---------------------------------------------------------------------------------------------------------------------------------------------------------------------------------------------------------------------------------------------------------------

C1_Q19_B7 (sum) C1_Q19_B7

---------------------------------------------------------------------------------------------------------------------------------------------------------------------------------------------------------------------------------------------------------------

type: numeric (double)

label: CPASS1, but 1 nonmissing value is not labeled

range: [0,6] units: 1

unique values: 7 missing .: 0/302

tabulation: Freq. Numeric Label

1 0

174 1 not at all

58 2 minimal

39 3 mild

18 4 moderate

9 5 severe

3 6 extreme

---------------------------------------------------------------------------------------------------------------------------------------------------------------------------------------------------------------------------------------------------------------

C1_Q19_A1 (sum) C1_Q19_A1

---------------------------------------------------------------------------------------------------------------------------------------------------------------------------------------------------------------------------------------------------------------

type: numeric (double)

label: CPASS1

range: [1,6] units: 1

unique values: 6 missing .: 0/302

tabulation: Freq. Numeric Label

141 1 not at all

64 2 minimal

53 3 mild

29 4 moderate

9 5 severe

6 6 extreme

---------------------------------------------------------------------------------------------------------------------------------------------------------------------------------------------------------------------------------------------------------------

C1_Q19_A2 (sum) C1_Q19_A2

---------------------------------------------------------------------------------------------------------------------------------------------------------------------------------------------------------------------------------------------------------------

type: numeric (double)

label: CPASS1

range: [1,6] units: 1

unique values: 6 missing .: 0/302

tabulation: Freq. Numeric Label

168 1 not at all

56 2 minimal

51 3 mild

18 4 moderate

5 5 severe

4 6 extreme

---------------------------------------------------------------------------------------------------------------------------------------------------------------------------------------------------------------------------------------------------------------

C1_Q19_A3 (sum) C1_Q19_A3

---------------------------------------------------------------------------------------------------------------------------------------------------------------------------------------------------------------------------------------------------------------

type: numeric (double)

label: CPASS1

range: [1,6] units: 1

unique values: 6 missing .: 0/302

tabulation: Freq. Numeric Label

194 1 not at all

64 2 minimal

31 3 mild

9 4 moderate

3 5 severe

1 6 extreme

---------------------------------------------------------------------------------------------------------------------------------------------------------------------------------------------------------------------------------------------------------------

C1_Q19_A4 (sum) C1_Q19_A4

---------------------------------------------------------------------------------------------------------------------------------------------------------------------------------------------------------------------------------------------------------------

type: numeric (double)

label: CPASS1

range: [1,5] units: 1

unique values: 5 missing .: 0/302

tabulation: Freq. Numeric Label

231 1 not at all

42 2 minimal

19 3 mild

8 4 moderate

2 5 severe

---------------------------------------------------------------------------------------------------------------------------------------------------------------------------------------------------------------------------------------------------------------

C1_Q19_A5 (sum) C1_Q19_A5

---------------------------------------------------------------------------------------------------------------------------------------------------------------------------------------------------------------------------------------------------------------

type: numeric (double)

label: CPASS1

range: [1,4] units: 1

unique values: 4 missing .: 0/302

tabulation: Freq. Numeric Label

260 1 not at all

28 2 minimal

11 3 mild

3 4 moderate

---------------------------------------------------------------------------------------------------------------------------------------------------------------------------------------------------------------------------------------------------------------

C1_Q19_A6 (sum) C1_Q19_A6

---------------------------------------------------------------------------------------------------------------------------------------------------------------------------------------------------------------------------------------------------------------

type: numeric (double)

label: CPASS1

range: [1,4] units: 1

unique values: 4 missing .: 0/302

tabulation: Freq. Numeric Label

282 1 not at all

13 2 minimal

4 3 mild

3 4 moderate

---------------------------------------------------------------------------------------------------------------------------------------------------------------------------------------------------------------------------------------------------------------

C1_Q19_A7 (sum) C1_Q19_A7

---------------------------------------------------------------------------------------------------------------------------------------------------------------------------------------------------------------------------------------------------------------

type: numeric (double)

label: CPASS1

range: [1,4] units: 1

unique values: 4 missing .: 0/302

tabulation: Freq. Numeric Label

284 1 not at all

11 2 minimal

6 3 mild

1 4 moderate

---------------------------------------------------------------------------------------------------------------------------------------------------------------------------------------------------------------------------------------------------------------

C1_Q19_A8 (sum) C1_Q19_A8

---------------------------------------------------------------------------------------------------------------------------------------------------------------------------------------------------------------------------------------------------------------

type: numeric (double)

label: CPASS1

range: [1,4] units: 1

unique values: 4 missing .: 0/302

tabulation: Freq. Numeric Label

286 1 not at all

10 2 minimal

3 3 mild

3 4 moderate

---------------------------------------------------------------------------------------------------------------------------------------------------------------------------------------------------------------------------------------------------------------

C1_Q19_A9 (sum) C1_Q19_A9

---------------------------------------------------------------------------------------------------------------------------------------------------------------------------------------------------------------------------------------------------------------

type: numeric (double)

label: CPASS1

range: [1,4] units: 1

unique values: 4 missing .: 0/302

tabulation: Freq. Numeric Label

289 1 not at all

8 2 minimal

4 3 mild

1 4 moderate

---------------------------------------------------------------------------------------------------------------------------------------------------------------------------------------------------------------------------------------------------------------

C1_Q19_A10 (sum) C1_Q19_A10

---------------------------------------------------------------------------------------------------------------------------------------------------------------------------------------------------------------------------------------------------------------

type: numeric (double)

label: CPASS1

range: [1,4] units: 1

unique values: 4 missing .: 0/302

tabulation: Freq. Numeric Label

285 1 not at all

13 2 minimal

3 3 mild

1 4 moderate

---------------------------------------------------------------------------------------------------------------------------------------------------------------------------------------------------------------------------------------------------------------

C1_Q21_B1 (sum) C1_Q20_B1

---------------------------------------------------------------------------------------------------------------------------------------------------------------------------------------------------------------------------------------------------------------

type: numeric (double)

label: CPASS1, but 1 nonmissing value is not labeled

range: [0,6] units: 1

unique values: 7 missing .: 0/302

tabulation: Freq. Numeric Label

1 0

222 1 not at all

35 2 minimal

28 3 mild

11 4 moderate

2 5 severe

3 6 extreme

---------------------------------------------------------------------------------------------------------------------------------------------------------------------------------------------------------------------------------------------------------------

C1_Q21_B2 (sum) C1_Q20_B2

---------------------------------------------------------------------------------------------------------------------------------------------------------------------------------------------------------------------------------------------------------------

type: numeric (double)

label: CPASS1, but 1 nonmissing value is not labeled

range: [0,5] units: 1

unique values: 6 missing .: 0/302

tabulation: Freq. Numeric Label

1 0

224 1 not at all

39 2 minimal

23 3 mild

11 4 moderate

4 5 severe

---------------------------------------------------------------------------------------------------------------------------------------------------------------------------------------------------------------------------------------------------------------

C1_Q21_B3 (sum) C1_Q20_B3

---------------------------------------------------------------------------------------------------------------------------------------------------------------------------------------------------------------------------------------------------------------

type: numeric (double)

label: CPASS1

range: [1,6] units: 1

unique values: 6 missing .: 0/302

tabulation: Freq. Numeric Label

220 1 not at all

39 2 minimal

23 3 mild

16 4 moderate

3 5 severe

1 6 extreme

---------------------------------------------------------------------------------------------------------------------------------------------------------------------------------------------------------------------------------------------------------------

C1_Q21_B4 (sum) C1_Q20_B4

---------------------------------------------------------------------------------------------------------------------------------------------------------------------------------------------------------------------------------------------------------------

type: numeric (double)

label: CPASS1

range: [1,6] units: 1

unique values: 6 missing .: 0/302

tabulation: Freq. Numeric Label

223 1 not at all

38 2 minimal

24 3 mild

14 4 moderate

2 5 severe

1 6 extreme

---------------------------------------------------------------------------------------------------------------------------------------------------------------------------------------------------------------------------------------------------------------

C1_Q21_B5 (sum) C1_Q20_B5

---------------------------------------------------------------------------------------------------------------------------------------------------------------------------------------------------------------------------------------------------------------

type: numeric (double)

label: CPASS1, but 1 nonmissing value is not labeled

range: [0,6] units: 1

unique values: 7 missing .: 0/302

tabulation: Freq. Numeric Label

1 0

212 1 not at all

39 2 minimal

27 3 mild

15 4 moderate

7 5 severe

1 6 extreme

---------------------------------------------------------------------------------------------------------------------------------------------------------------------------------------------------------------------------------------------------------------

C1_Q21_B6 (sum) C1_Q20_B6

---------------------------------------------------------------------------------------------------------------------------------------------------------------------------------------------------------------------------------------------------------------

type: numeric (double)

label: CPASS1

range: [1,6] units: 1

unique values: 6 missing .: 0/302

tabulation: Freq. Numeric Label

216 1 not at all

40 2 minimal

24 3 mild

16 4 moderate

5 5 severe

1 6 extreme

---------------------------------------------------------------------------------------------------------------------------------------------------------------------------------------------------------------------------------------------------------------

C1_Q21_B7 (sum) C1_Q20_B7

---------------------------------------------------------------------------------------------------------------------------------------------------------------------------------------------------------------------------------------------------------------

type: numeric (double)

label: CPASS1, but 1 nonmissing value is not labeled

range: [0,6] units: 1

unique values: 7 missing .: 0/302

tabulation: Freq. Numeric Label

1 0

199 1 not at all

42 2 minimal

33 3 mild

18 4 moderate

7 5 severe

2 6 extreme

---------------------------------------------------------------------------------------------------------------------------------------------------------------------------------------------------------------------------------------------------------------

C1_Q21_A1 (sum) C1_Q20_A1

---------------------------------------------------------------------------------------------------------------------------------------------------------------------------------------------------------------------------------------------------------------

type: numeric (double)

label: CPASS1

range: [1,6] units: 1

unique values: 6 missing .: 0/302

tabulation: Freq. Numeric Label

170 1 not at all

70 2 minimal

33 3 mild

21 4 moderate

7 5 severe

1 6 extreme

---------------------------------------------------------------------------------------------------------------------------------------------------------------------------------------------------------------------------------------------------------------

C1_Q21_A2 (sum) C1_Q20_A2

---------------------------------------------------------------------------------------------------------------------------------------------------------------------------------------------------------------------------------------------------------------

type: numeric (double)

label: CPASS1

range: [1,6] units: 1

unique values: 6 missing .: 0/302

tabulation: Freq. Numeric Label

176 1 not at all

52 2 minimal

42 3 mild

26 4 moderate

5 5 severe

1 6 extreme

---------------------------------------------------------------------------------------------------------------------------------------------------------------------------------------------------------------------------------------------------------------

C1_Q21_A3 (sum) C1_Q20_A3

---------------------------------------------------------------------------------------------------------------------------------------------------------------------------------------------------------------------------------------------------------------

type: numeric (double)

label: CPASS1

range: [1,6] units: 1

unique values: 6 missing .: 0/302

tabulation: Freq. Numeric Label

199 1 not at all

45 2 minimal

35 3 mild

20 4 moderate

2 5 severe

1 6 extreme

---------------------------------------------------------------------------------------------------------------------------------------------------------------------------------------------------------------------------------------------------------------

C1_Q21_A4 (sum) C1_Q20_A4

---------------------------------------------------------------------------------------------------------------------------------------------------------------------------------------------------------------------------------------------------------------

type: numeric (double)

label: CPASS1

range: [1,5] units: 1

unique values: 5 missing .: 0/302

tabulation: Freq. Numeric Label

218 1 not at all

36 2 minimal

27 3 mild

15 4 moderate

6 5 severe

---------------------------------------------------------------------------------------------------------------------------------------------------------------------------------------------------------------------------------------------------------------

C1_Q21_A5 (sum) C1_Q20_A5

---------------------------------------------------------------------------------------------------------------------------------------------------------------------------------------------------------------------------------------------------------------

type: numeric (double)

label: CPASS1

range: [1,6] units: 1

unique values: 6 missing .: 0/302

tabulation: Freq. Numeric Label

227 1 not at all

37 2 minimal

24 3 mild

11 4 moderate

2 5 severe

1 6 extreme

---------------------------------------------------------------------------------------------------------------------------------------------------------------------------------------------------------------------------------------------------------------

C1_Q21_A6 (sum) C1_Q20_A6

---------------------------------------------------------------------------------------------------------------------------------------------------------------------------------------------------------------------------------------------------------------

type: numeric (double)

label: CPASS1

range: [1,6] units: 1

unique values: 6 missing .: 0/302

tabulation: Freq. Numeric Label

236 1 not at all

27 2 minimal

28 3 mild

7 4 moderate

3 5 severe

1 6 extreme

---------------------------------------------------------------------------------------------------------------------------------------------------------------------------------------------------------------------------------------------------------------

C1_Q21_A7 (sum) C1_Q20_A7

---------------------------------------------------------------------------------------------------------------------------------------------------------------------------------------------------------------------------------------------------------------

type: numeric (double)

label: CPASS1

range: [1,6] units: 1

unique values: 5 missing .: 0/302

tabulation: Freq. Numeric Label

244 1 not at all

25 2 minimal

23 3 mild

8 4 moderate

2 6 extreme

---------------------------------------------------------------------------------------------------------------------------------------------------------------------------------------------------------------------------------------------------------------

C1_Q21_A8 (sum) C1_Q20_A8

---------------------------------------------------------------------------------------------------------------------------------------------------------------------------------------------------------------------------------------------------------------

type: numeric (double)

label: CPASS1

range: [1,5] units: 1

unique values: 5 missing .: 0/302

tabulation: Freq. Numeric Label

254 1 not at all

23 2 minimal

15 3 mild

9 4 moderate

1 5 severe

---------------------------------------------------------------------------------------------------------------------------------------------------------------------------------------------------------------------------------------------------------------

C1_Q21_A9 (sum) C1_Q20_A9

---------------------------------------------------------------------------------------------------------------------------------------------------------------------------------------------------------------------------------------------------------------

type: numeric (double)

label: CPASS1

range: [1,5] units: 1

unique values: 5 missing .: 0/302

tabulation: Freq. Numeric Label

249 1 not at all

25 2 minimal

16 3 mild

9 4 moderate

3 5 severe

---------------------------------------------------------------------------------------------------------------------------------------------------------------------------------------------------------------------------------------------------------------

C1_Q21_A10 (sum) C1_Q20_A10

---------------------------------------------------------------------------------------------------------------------------------------------------------------------------------------------------------------------------------------------------------------

type: numeric (double)

label: CPASS1

range: [1,5] units: 1

unique values: 5 missing .: 0/302

tabulation: Freq. Numeric Label

247 1 not at all

23 2 minimal

15 3 mild

15 4 moderate

2 5 severe

---------------------------------------------------------------------------------------------------------------------------------------------------------------------------------------------------------------------------------------------------------------

C1_Q20_B1 (sum) C1_Q21_B1

---------------------------------------------------------------------------------------------------------------------------------------------------------------------------------------------------------------------------------------------------------------

type: numeric (double)

label: CPASS1, but 1 nonmissing value is not labeled

range: [0,5] units: 1

unique values: 6 missing .: 0/302

tabulation: Freq. Numeric Label

1 0

233 1 not at all

32 2 minimal

18 3 mild

16 4 moderate

2 5 severe

---------------------------------------------------------------------------------------------------------------------------------------------------------------------------------------------------------------------------------------------------------------

C1_Q20_B2 (sum) C1_Q21_B2

---------------------------------------------------------------------------------------------------------------------------------------------------------------------------------------------------------------------------------------------------------------

type: numeric (double)

label: CPASS1, but 1 nonmissing value is not labeled

range: [0,6] units: 1

unique values: 7 missing .: 0/302

tabulation: Freq. Numeric Label

1 0

227 1 not at all

36 2 minimal

21 3 mild

11 4 moderate

5 5 severe

1 6 extreme

---------------------------------------------------------------------------------------------------------------------------------------------------------------------------------------------------------------------------------------------------------------

C1_Q20_B3 (sum) C1_Q21_B3

---------------------------------------------------------------------------------------------------------------------------------------------------------------------------------------------------------------------------------------------------------------

type: numeric (double)

label: CPASS1

range: [1,5] units: 1

unique values: 5 missing .: 0/302

tabulation: Freq. Numeric Label

220 1 not at all

32 2 minimal

28 3 mild

18 4 moderate

4 5 severe

---------------------------------------------------------------------------------------------------------------------------------------------------------------------------------------------------------------------------------------------------------------

C1_Q20_B4 (sum) C1_Q21_B4

---------------------------------------------------------------------------------------------------------------------------------------------------------------------------------------------------------------------------------------------------------------

type: numeric (double)

label: CPASS1

range: [1,5] units: 1

unique values: 5 missing .: 0/302

tabulation: Freq. Numeric Label

223 1 not at all

33 2 minimal

28 3 mild

15 4 moderate

3 5 severe

---------------------------------------------------------------------------------------------------------------------------------------------------------------------------------------------------------------------------------------------------------------

C1_Q20_B5 (sum) C1_Q21_B5

---------------------------------------------------------------------------------------------------------------------------------------------------------------------------------------------------------------------------------------------------------------

type: numeric (double)

label: CPASS1, but 1 nonmissing value is not labeled

range: [0,6] units: 1

unique values: 7 missing .: 0/302

tabulation: Freq. Numeric Label

1 0

216 1 not at all

33 2 minimal

27 3 mild

19 4 moderate

5 5 severe

1 6 extreme

---------------------------------------------------------------------------------------------------------------------------------------------------------------------------------------------------------------------------------------------------------------

C1_Q20_B6 (sum) C1_Q21_B6

---------------------------------------------------------------------------------------------------------------------------------------------------------------------------------------------------------------------------------------------------------------

type: numeric (double)

label: CPASS1

range: [1,6] units: 1

unique values: 6 missing .: 0/302

tabulation: Freq. Numeric Label

214 1 not at all

34 2 minimal

29 3 mild

15 4 moderate

7 5 severe

3 6 extreme

---------------------------------------------------------------------------------------------------------------------------------------------------------------------------------------------------------------------------------------------------------------

C1_Q20_B7 (sum) C1_Q21_B7

---------------------------------------------------------------------------------------------------------------------------------------------------------------------------------------------------------------------------------------------------------------

type: numeric (double)

label: CPASS1, but 1 nonmissing value is not labeled

range: [0,6] units: 1

unique values: 7 missing .: 0/302

tabulation: Freq. Numeric Label

1 0

191 1 not at all

44 2 minimal

33 3 mild

23 4 moderate

8 5 severe

2 6 extreme

---------------------------------------------------------------------------------------------------------------------------------------------------------------------------------------------------------------------------------------------------------------

C1_Q20_A1 (sum) C1_Q21_A1

---------------------------------------------------------------------------------------------------------------------------------------------------------------------------------------------------------------------------------------------------------------

type: numeric (double)

label: CPASS1

range: [1,6] units: 1

unique values: 6 missing .: 0/302

tabulation: Freq. Numeric Label

141 1 not at all

59 2 minimal

48 3 mild

28 4 moderate

21 5 severe

5 6 extreme

---------------------------------------------------------------------------------------------------------------------------------------------------------------------------------------------------------------------------------------------------------------

C1_Q20_A2 (sum) C1_Q21_A2

---------------------------------------------------------------------------------------------------------------------------------------------------------------------------------------------------------------------------------------------------------------

type: numeric (double)

label: CPASS1

range: [1,6] units: 1

unique values: 6 missing .: 0/302

tabulation: Freq. Numeric Label

146 1 not at all

66 2 minimal

42 3 mild

31 4 moderate

8 5 severe

9 6 extreme

---------------------------------------------------------------------------------------------------------------------------------------------------------------------------------------------------------------------------------------------------------------

C1_Q20_A3 (sum) C1_Q21_A3

---------------------------------------------------------------------------------------------------------------------------------------------------------------------------------------------------------------------------------------------------------------

type: numeric (double)

label: CPASS1

range: [1,6] units: 1

unique values: 6 missing .: 0/302

tabulation: Freq. Numeric Label

184 1 not at all

50 2 minimal

39 3 mild

23 4 moderate

5 5 severe

1 6 extreme

---------------------------------------------------------------------------------------------------------------------------------------------------------------------------------------------------------------------------------------------------------------

C1_Q20_A4 (sum) C1_Q21_A4

---------------------------------------------------------------------------------------------------------------------------------------------------------------------------------------------------------------------------------------------------------------

type: numeric (double)

label: CPASS1

range: [1,5] units: 1

unique values: 5 missing .: 0/302

tabulation: Freq. Numeric Label

208 1 not at all

45 2 minimal

29 3 mild

16 4 moderate

4 5 severe

---------------------------------------------------------------------------------------------------------------------------------------------------------------------------------------------------------------------------------------------------------------

C1_Q20_A5 (sum) C1_Q21_A5

---------------------------------------------------------------------------------------------------------------------------------------------------------------------------------------------------------------------------------------------------------------

type: numeric (double)

label: CPASS1

range: [1,5] units: 1

unique values: 5 missing .: 0/302

tabulation: Freq. Numeric Label

231 1 not at all

39 2 minimal

25 3 mild

6 4 moderate

1 5 severe

---------------------------------------------------------------------------------------------------------------------------------------------------------------------------------------------------------------------------------------------------------------

C1_Q20_A6 (sum) C1_Q21_A6

---------------------------------------------------------------------------------------------------------------------------------------------------------------------------------------------------------------------------------------------------------------

type: numeric (double)

label: CPASS1

range: [1,5] units: 1

unique values: 5 missing .: 0/302

tabulation: Freq. Numeric Label

239 1 not at all

38 2 minimal

18 3 mild

5 4 moderate

2 5 severe

---------------------------------------------------------------------------------------------------------------------------------------------------------------------------------------------------------------------------------------------------------------

C1_Q20_A7 (sum) C1_Q21_A7

---------------------------------------------------------------------------------------------------------------------------------------------------------------------------------------------------------------------------------------------------------------

type: numeric (double)

label: CPASS1

range: [1,6] units: 1

unique values: 6 missing .: 0/302

tabulation: Freq. Numeric Label

251 1 not at all

26 2 minimal

17 3 mild

6 4 moderate

1 5 severe

1 6 extreme

---------------------------------------------------------------------------------------------------------------------------------------------------------------------------------------------------------------------------------------------------------------

C1_Q20_A8 (sum) C1_Q21_A8

---------------------------------------------------------------------------------------------------------------------------------------------------------------------------------------------------------------------------------------------------------------

type: numeric (double)

label: CPASS1

range: [1,5] units: 1

unique values: 5 missing .: 0/302

tabulation: Freq. Numeric Label

259 1 not at all

24 2 minimal

11 3 mild

6 4 moderate

2 5 severe

---------------------------------------------------------------------------------------------------------------------------------------------------------------------------------------------------------------------------------------------------------------

C1_Q20_A9 (sum) C1_Q21_A9

---------------------------------------------------------------------------------------------------------------------------------------------------------------------------------------------------------------------------------------------------------------

type: numeric (double)

label: CPASS1

range: [1,5] units: 1

unique values: 5 missing .: 0/302

tabulation: Freq. Numeric Label

262 1 not at all

20 2 minimal

12 3 mild

7 4 moderate

1 5 severe

---------------------------------------------------------------------------------------------------------------------------------------------------------------------------------------------------------------------------------------------------------------

C1_Q20_A10 (sum) C1_Q21_A10

---------------------------------------------------------------------------------------------------------------------------------------------------------------------------------------------------------------------------------------------------------------

type: numeric (double)

label: CPASS1

range: [1,5] units: 1

unique values: 5 missing .: 0/302

tabulation: Freq. Numeric Label

257 1 not at all

29 2 minimal

11 3 mild

4 4 moderate

1 5 severe

---------------------------------------------------------------------------------------------------------------------------------------------------------------------------------------------------------------------------------------------------------------

C1_Q22_B1 (sum) C1_Q22_B1

---------------------------------------------------------------------------------------------------------------------------------------------------------------------------------------------------------------------------------------------------------------

type: numeric (double)

label: CPASS1, but 1 nonmissing value is not labeled

range: [0,5] units: 1

unique values: 6 missing .: 0/302

tabulation: Freq. Numeric Label

1 0

176 1 not at all

55 2 minimal

36 3 mild

26 4 moderate

8 5 severe

---------------------------------------------------------------------------------------------------------------------------------------------------------------------------------------------------------------------------------------------------------------

C1_Q22_B2 (sum) C1_Q22_B2

---------------------------------------------------------------------------------------------------------------------------------------------------------------------------------------------------------------------------------------------------------------

type: numeric (double)

label: CPASS1, but 1 nonmissing value is not labeled

range: [0,5] units: 1

unique values: 6 missing .: 0/302

tabulation: Freq. Numeric Label

1 0

183 1 not at all

60 2 minimal

31 3 mild

18 4 moderate

9 5 severe

---------------------------------------------------------------------------------------------------------------------------------------------------------------------------------------------------------------------------------------------------------------

C1_Q22_B3 (sum) C1_Q22_B3

---------------------------------------------------------------------------------------------------------------------------------------------------------------------------------------------------------------------------------------------------------------

type: numeric (double)

label: CPASS1

range: [1,5] units: 1

unique values: 5 missing .: 0/302

tabulation: Freq. Numeric Label

184 1 not at all

54 2 minimal

40 3 mild

17 4 moderate

7 5 severe

---------------------------------------------------------------------------------------------------------------------------------------------------------------------------------------------------------------------------------------------------------------

C1_Q22_B4 (sum) C1_Q22_B4

---------------------------------------------------------------------------------------------------------------------------------------------------------------------------------------------------------------------------------------------------------------

type: numeric (double)

label: CPASS1

range: [1,6] units: 1

unique values: 6 missing .: 0/302

tabulation: Freq. Numeric Label

180 1 not at all

54 2 minimal

41 3 mild

21 4 moderate

5 5 severe

1 6 extreme

---------------------------------------------------------------------------------------------------------------------------------------------------------------------------------------------------------------------------------------------------------------

C1_Q22_B5 (sum) C1_Q22_B5

---------------------------------------------------------------------------------------------------------------------------------------------------------------------------------------------------------------------------------------------------------------

type: numeric (double)

label: CPASS1, but 1 nonmissing value is not labeled

range: [0,6] units: 1

unique values: 7 missing .: 0/302

tabulation: Freq. Numeric Label

1 0

174 1 not at all

58 2 minimal

44 3 mild

18 4 moderate

6 5 severe

1 6 extreme

---------------------------------------------------------------------------------------------------------------------------------------------------------------------------------------------------------------------------------------------------------------

C1_Q22_B6 (sum) C1_Q22_B6

---------------------------------------------------------------------------------------------------------------------------------------------------------------------------------------------------------------------------------------------------------------

type: numeric (double)

label: CPASS1

range: [1,6] units: 1

unique values: 6 missing .: 0/302

tabulation: Freq. Numeric Label

187 1 not at all

54 2 minimal

34 3 mild

19 4 moderate

5 5 severe

3 6 extreme

---------------------------------------------------------------------------------------------------------------------------------------------------------------------------------------------------------------------------------------------------------------

C1_Q22_B7 (sum) C1_Q22_B7

---------------------------------------------------------------------------------------------------------------------------------------------------------------------------------------------------------------------------------------------------------------

type: numeric (double)

label: CPASS1, but 1 nonmissing value is not labeled

range: [0,6] units: 1

unique values: 7 missing .: 0/302

tabulation: Freq. Numeric Label

1 0

167 1 not at all

59 2 minimal

44 3 mild

20 4 moderate

8 5 severe

3 6 extreme

---------------------------------------------------------------------------------------------------------------------------------------------------------------------------------------------------------------------------------------------------------------

C1_Q22_A1 (sum) C1_Q22_A1

---------------------------------------------------------------------------------------------------------------------------------------------------------------------------------------------------------------------------------------------------------------

type: numeric (double)

label: CPASS1

range: [1,6] units: 1

unique values: 6 missing .: 0/302

tabulation: Freq. Numeric Label

123 1 not at all

76 2 minimal

55 3 mild

35 4 moderate

11 5 severe

2 6 extreme

---------------------------------------------------------------------------------------------------------------------------------------------------------------------------------------------------------------------------------------------------------------

C1_Q22_A2 (sum) C1_Q22_A2

---------------------------------------------------------------------------------------------------------------------------------------------------------------------------------------------------------------------------------------------------------------

type: numeric (double)

label: CPASS1

range: [1,6] units: 1

unique values: 6 missing .: 0/302

tabulation: Freq. Numeric Label

139 1 not at all

70 2 minimal

48 3 mild

27 4 moderate

13 5 severe

5 6 extreme

---------------------------------------------------------------------------------------------------------------------------------------------------------------------------------------------------------------------------------------------------------------

C1_Q22_A3 (sum) C1_Q22_A3

---------------------------------------------------------------------------------------------------------------------------------------------------------------------------------------------------------------------------------------------------------------

type: numeric (double)

label: CPASS1

range: [1,6] units: 1

unique values: 6 missing .: 0/302

tabulation: Freq. Numeric Label

161 1 not at all

63 2 minimal

46 3 mild

20 4 moderate

10 5 severe

2 6 extreme

---------------------------------------------------------------------------------------------------------------------------------------------------------------------------------------------------------------------------------------------------------------

C1_Q22_A4 (sum) C1_Q22_A4

---------------------------------------------------------------------------------------------------------------------------------------------------------------------------------------------------------------------------------------------------------------

type: numeric (double)

label: CPASS1

range: [1,6] units: 1

unique values: 6 missing .: 0/302

tabulation: Freq. Numeric Label

190 1 not at all

45 2 minimal

42 3 mild

17 4 moderate

7 5 severe

1 6 extreme

---------------------------------------------------------------------------------------------------------------------------------------------------------------------------------------------------------------------------------------------------------------

C1_Q22_A5 (sum) C1_Q22_A5

---------------------------------------------------------------------------------------------------------------------------------------------------------------------------------------------------------------------------------------------------------------

type: numeric (double)

label: CPASS1

range: [1,5] units: 1

unique values: 5 missing .: 0/302

tabulation: Freq. Numeric Label

211 1 not at all

53 2 minimal

24 3 mild

13 4 moderate

1 5 severe

---------------------------------------------------------------------------------------------------------------------------------------------------------------------------------------------------------------------------------------------------------------

C1_Q22_A6 (sum) C1_Q22_A6

---------------------------------------------------------------------------------------------------------------------------------------------------------------------------------------------------------------------------------------------------------------

type: numeric (double)

label: CPASS1

range: [1,5] units: 1

unique values: 5 missing .: 0/302

tabulation: Freq. Numeric Label

221 1 not at all

44 2 minimal

21 3 mild

13 4 moderate

3 5 severe

---------------------------------------------------------------------------------------------------------------------------------------------------------------------------------------------------------------------------------------------------------------

C1_Q22_A7 (sum) C1_Q22_A7

---------------------------------------------------------------------------------------------------------------------------------------------------------------------------------------------------------------------------------------------------------------

type: numeric (double)

label: CPASS1

range: [1,5] units: 1

unique values: 5 missing .: 0/302

tabulation: Freq. Numeric Label

226 1 not at all

41 2 minimal

22 3 mild

11 4 moderate

2 5 severe

---------------------------------------------------------------------------------------------------------------------------------------------------------------------------------------------------------------------------------------------------------------

C1_Q22_A8 (sum) C1_Q22_A8

---------------------------------------------------------------------------------------------------------------------------------------------------------------------------------------------------------------------------------------------------------------

type: numeric (double)

label: CPASS1

range: [1,5] units: 1

unique values: 5 missing .: 0/302

tabulation: Freq. Numeric Label

229 1 not at all

48 2 minimal

13 3 mild

7 4 moderate

5 5 severe

---------------------------------------------------------------------------------------------------------------------------------------------------------------------------------------------------------------------------------------------------------------

C1_Q22_A9 (sum) C1_Q22_A9

---------------------------------------------------------------------------------------------------------------------------------------------------------------------------------------------------------------------------------------------------------------

type: numeric (double)

label: CPASS1

range: [1,5] units: 1

unique values: 5 missing .: 0/302

tabulation: Freq. Numeric Label

241 1 not at all

34 2 minimal

13 3 mild

12 4 moderate

2 5 severe

---------------------------------------------------------------------------------------------------------------------------------------------------------------------------------------------------------------------------------------------------------------

C1_Q22_A10 (sum) C1_Q22_A10

---------------------------------------------------------------------------------------------------------------------------------------------------------------------------------------------------------------------------------------------------------------

type: numeric (double)

label: CPASS1

range: [1,6] units: 1

unique values: 6 missing .: 0/302

tabulation: Freq. Numeric Label

231 1 not at all

39 2 minimal

23 3 mild

5 4 moderate

3 5 severe

1 6 extreme

---------------------------------------------------------------------------------------------------------------------------------------------------------------------------------------------------------------------------------------------------------------

C1_Q23_B1 (sum) C1_Q23_B1

---------------------------------------------------------------------------------------------------------------------------------------------------------------------------------------------------------------------------------------------------------------

type: numeric (double)

label: CPASS1, but 1 nonmissing value is not labeled

range: [0,6] units: 1

unique values: 7 missing .: 0/302

tabulation: Freq. Numeric Label

1 0

208 1 not at all

38 2 minimal

32 3 mild

15 4 moderate

7 5 severe

1 6 extreme

---------------------------------------------------------------------------------------------------------------------------------------------------------------------------------------------------------------------------------------------------------------

C1_Q23_B2 (sum) C1_Q23_B2

---------------------------------------------------------------------------------------------------------------------------------------------------------------------------------------------------------------------------------------------------------------

type: numeric (double)

label: CPASS1, but 1 nonmissing value is not labeled

range: [0,5] units: 1

unique values: 6 missing .: 0/302

tabulation: Freq. Numeric Label

1 0

201 1 not at all

55 2 minimal

25 3 mild

14 4 moderate

6 5 severe

---------------------------------------------------------------------------------------------------------------------------------------------------------------------------------------------------------------------------------------------------------------

C1_Q23_B3 (sum) C1_Q23_B3

---------------------------------------------------------------------------------------------------------------------------------------------------------------------------------------------------------------------------------------------------------------

type: numeric (double)

label: CPASS1

range: [1,5] units: 1

unique values: 5 missing .: 0/302

tabulation: Freq. Numeric Label

204 1 not at all

46 2 minimal

30 3 mild

16 4 moderate

6 5 severe

---------------------------------------------------------------------------------------------------------------------------------------------------------------------------------------------------------------------------------------------------------------

C1_Q23_B4 (sum) C1_Q23_B4

---------------------------------------------------------------------------------------------------------------------------------------------------------------------------------------------------------------------------------------------------------------

type: numeric (double)

label: CPASS1

range: [1,6] units: 1

unique values: 6 missing .: 0/302

tabulation: Freq. Numeric Label

211 1 not at all

39 2 minimal

33 3 mild

13 4 moderate

5 5 severe

1 6 extreme

---------------------------------------------------------------------------------------------------------------------------------------------------------------------------------------------------------------------------------------------------------------

C1_Q23_B5 (sum) C1_Q23_B5

---------------------------------------------------------------------------------------------------------------------------------------------------------------------------------------------------------------------------------------------------------------

type: numeric (double)

label: CPASS1, but 1 nonmissing value is not labeled

range: [0,6] units: 1

unique values: 7 missing .: 0/302

tabulation: Freq. Numeric Label

1 0

199 1 not at all

52 2 minimal

33 3 mild

11 4 moderate

5 5 severe

1 6 extreme

---------------------------------------------------------------------------------------------------------------------------------------------------------------------------------------------------------------------------------------------------------------

C1_Q23_B6 (sum) C1_Q23_B6

---------------------------------------------------------------------------------------------------------------------------------------------------------------------------------------------------------------------------------------------------------------

type: numeric (double)

label: CPASS1

range: [1,6] units: 1

unique values: 6 missing .: 0/302

tabulation: Freq. Numeric Label

202 1 not at all

51 2 minimal

28 3 mild

14 4 moderate

5 5 severe

2 6 extreme

---------------------------------------------------------------------------------------------------------------------------------------------------------------------------------------------------------------------------------------------------------------

C1_Q23_B7 (sum) C1_Q23_B7

---------------------------------------------------------------------------------------------------------------------------------------------------------------------------------------------------------------------------------------------------------------

type: numeric (double)

label: CPASS1, but 1 nonmissing value is not labeled

range: [0,6] units: 1

unique values: 7 missing .: 0/302

tabulation: Freq. Numeric Label

1 0

200 1 not at all

40 2 minimal

35 3 mild

17 4 moderate

7 5 severe

2 6 extreme

---------------------------------------------------------------------------------------------------------------------------------------------------------------------------------------------------------------------------------------------------------------

C1_Q23_A1 (sum) C1_Q23_A1

---------------------------------------------------------------------------------------------------------------------------------------------------------------------------------------------------------------------------------------------------------------

type: numeric (double)

label: CPASS1

range: [1,6] units: 1

unique values: 6 missing .: 0/302

tabulation: Freq. Numeric Label

145 1 not at all

63 2 minimal

53 3 mild

30 4 moderate

10 5 severe

1 6 extreme

---------------------------------------------------------------------------------------------------------------------------------------------------------------------------------------------------------------------------------------------------------------

C1_Q23_A2 (sum) C1_Q23_A2

---------------------------------------------------------------------------------------------------------------------------------------------------------------------------------------------------------------------------------------------------------------

type: numeric (double)

label: CPASS1

range: [1,6] units: 1

unique values: 6 missing .: 0/302

tabulation: Freq. Numeric Label

165 1 not at all

64 2 minimal

34 3 mild

29 4 moderate

5 5 severe

5 6 extreme

---------------------------------------------------------------------------------------------------------------------------------------------------------------------------------------------------------------------------------------------------------------

C1_Q23_A3 (sum) C1_Q23_A3

---------------------------------------------------------------------------------------------------------------------------------------------------------------------------------------------------------------------------------------------------------------

type: numeric (double)

label: CPASS1

range: [1,6] units: 1

unique values: 6 missing .: 0/302

tabulation: Freq. Numeric Label

186 1 not at all

53 2 minimal

38 3 mild

18 4 moderate

5 5 severe

2 6 extreme

---------------------------------------------------------------------------------------------------------------------------------------------------------------------------------------------------------------------------------------------------------------

C1_Q23_A4 (sum) C1_Q23_A4

---------------------------------------------------------------------------------------------------------------------------------------------------------------------------------------------------------------------------------------------------------------

type: numeric (double)

label: CPASS1

range: [1,6] units: 1

unique values: 6 missing .: 0/302

tabulation: Freq. Numeric Label

210 1 not at all

44 2 minimal

29 3 mild

14 4 moderate

4 5 severe

1 6 extreme

---------------------------------------------------------------------------------------------------------------------------------------------------------------------------------------------------------------------------------------------------------------

C1_Q23_A5 (sum) C1_Q23_A5

---------------------------------------------------------------------------------------------------------------------------------------------------------------------------------------------------------------------------------------------------------------

type: numeric (double)

label: CPASS1

range: [1,4] units: 1

unique values: 4 missing .: 0/302

tabulation: Freq. Numeric Label

229 1 not at all

40 2 minimal

19 3 mild

14 4 moderate

---------------------------------------------------------------------------------------------------------------------------------------------------------------------------------------------------------------------------------------------------------------

C1_Q23_A6 (sum) C1_Q23_A6

---------------------------------------------------------------------------------------------------------------------------------------------------------------------------------------------------------------------------------------------------------------

type: numeric (double)

label: CPASS1

range: [1,5] units: 1

unique values: 5 missing .: 0/302

tabulation: Freq. Numeric Label

236 1 not at all

36 2 minimal

20 3 mild

8 4 moderate

2 5 severe

---------------------------------------------------------------------------------------------------------------------------------------------------------------------------------------------------------------------------------------------------------------

C1_Q23_A7 (sum) C1_Q23_A7

---------------------------------------------------------------------------------------------------------------------------------------------------------------------------------------------------------------------------------------------------------------

type: numeric (double)

label: CPASS1

range: [1,5] units: 1

unique values: 5 missing .: 0/302

tabulation: Freq. Numeric Label

243 1 not at all

36 2 minimal

14 3 mild

8 4 moderate

1 5 severe

---------------------------------------------------------------------------------------------------------------------------------------------------------------------------------------------------------------------------------------------------------------

C1_Q23_A8 (sum) C1_Q23_A8

---------------------------------------------------------------------------------------------------------------------------------------------------------------------------------------------------------------------------------------------------------------

type: numeric (double)

label: CPASS1

range: [1,4] units: 1

unique values: 4 missing .: 0/302

tabulation: Freq. Numeric Label

251 1 not at all

33 2 minimal

12 3 mild

6 4 moderate

---------------------------------------------------------------------------------------------------------------------------------------------------------------------------------------------------------------------------------------------------------------

C1_Q23_A9 (sum) C1_Q23_A9

---------------------------------------------------------------------------------------------------------------------------------------------------------------------------------------------------------------------------------------------------------------

type: numeric (double)

label: CPASS1

range: [1,5] units: 1

unique values: 5 missing .: 0/302

tabulation: Freq. Numeric Label

249 1 not at all

37 2 minimal

5 3 mild

10 4 moderate

1 5 severe

---------------------------------------------------------------------------------------------------------------------------------------------------------------------------------------------------------------------------------------------------------------

C1_Q23_A10 (sum) C1_Q23_A10

---------------------------------------------------------------------------------------------------------------------------------------------------------------------------------------------------------------------------------------------------------------

type: numeric (double)

label: CPASS1

range: [1,4] units: 1

unique values: 4 missing .: 0/302

tabulation: Freq. Numeric Label

248 1 not at all

33 2 minimal

17 3 mild

4 4 moderate

---------------------------------------------------------------------------------------------------------------------------------------------------------------------------------------------------------------------------------------------------------------

C1_Q24_B1 (sum) C1_Q24_B1

---------------------------------------------------------------------------------------------------------------------------------------------------------------------------------------------------------------------------------------------------------------

type: numeric (double)

label: CPASS1, but 1 nonmissing value is not labeled

range: [0,6] units: 1

unique values: 7 missing .: 0/302

tabulation: Freq. Numeric Label

1 0

221 1 not at all

34 2 minimal

25 3 mild

15 4 moderate

3 5 severe

3 6 extreme

---------------------------------------------------------------------------------------------------------------------------------------------------------------------------------------------------------------------------------------------------------------

C1_Q24_B2 (sum) C1_Q24_B2

---------------------------------------------------------------------------------------------------------------------------------------------------------------------------------------------------------------------------------------------------------------

type: numeric (double)

label: CPASS1, but 1 nonmissing value is not labeled

range: [0,6] units: 1

unique values: 7 missing .: 0/302

tabulation: Freq. Numeric Label

1 0

219 1 not at all

36 2 minimal

27 3 mild

12 4 moderate

6 5 severe

1 6 extreme

---------------------------------------------------------------------------------------------------------------------------------------------------------------------------------------------------------------------------------------------------------------

C1_Q24_B3 (sum) C1_Q24_B3

---------------------------------------------------------------------------------------------------------------------------------------------------------------------------------------------------------------------------------------------------------------

type: numeric (double)

label: CPASS1

range: [1,6] units: 1

unique values: 6 missing .: 0/302

tabulation: Freq. Numeric Label

220 1 not at all

41 2 minimal

21 3 mild

14 4 moderate

5 5 severe

1 6 extreme

---------------------------------------------------------------------------------------------------------------------------------------------------------------------------------------------------------------------------------------------------------------

C1_Q24_B4 (sum) C1_Q24_B4

---------------------------------------------------------------------------------------------------------------------------------------------------------------------------------------------------------------------------------------------------------------

type: numeric (double)

label: CPASS1

range: [1,6] units: 1

unique values: 6 missing .: 0/302

tabulation: Freq. Numeric Label

222 1 not at all

35 2 minimal

30 3 mild

8 4 moderate

6 5 severe

1 6 extreme

---------------------------------------------------------------------------------------------------------------------------------------------------------------------------------------------------------------------------------------------------------------

C1_Q24_B5 (sum) C1_Q24_B5

---------------------------------------------------------------------------------------------------------------------------------------------------------------------------------------------------------------------------------------------------------------

type: numeric (double)

label: CPASS1, but 1 nonmissing value is not labeled

range: [0,6] units: 1

unique values: 7 missing .: 0/302

tabulation: Freq. Numeric Label

1 0

209 1 not at all

47 2 minimal

29 3 mild

11 4 moderate

4 5 severe

1 6 extreme

---------------------------------------------------------------------------------------------------------------------------------------------------------------------------------------------------------------------------------------------------------------

C1_Q24_B6 (sum) C1_Q24_B6

---------------------------------------------------------------------------------------------------------------------------------------------------------------------------------------------------------------------------------------------------------------

type: numeric (double)

label: CPASS1

range: [1,6] units: 1

unique values: 6 missing .: 0/302

tabulation: Freq. Numeric Label

224 1 not at all

33 2 minimal

23 3 mild

13 4 moderate

7 5 severe

2 6 extreme

---------------------------------------------------------------------------------------------------------------------------------------------------------------------------------------------------------------------------------------------------------------

C1_Q24_B7 (sum) C1_Q24_B7

---------------------------------------------------------------------------------------------------------------------------------------------------------------------------------------------------------------------------------------------------------------

type: numeric (double)

label: CPASS1, but 1 nonmissing value is not labeled

range: [0,6] units: 1

unique values: 7 missing .: 0/302

tabulation: Freq. Numeric Label

1 0

206 1 not at all

40 2 minimal

35 3 mild

12 4 moderate

6 5 severe

2 6 extreme

---------------------------------------------------------------------------------------------------------------------------------------------------------------------------------------------------------------------------------------------------------------

C1_Q24_A1 (sum) C1_Q24_A1

---------------------------------------------------------------------------------------------------------------------------------------------------------------------------------------------------------------------------------------------------------------

type: numeric (double)

label: CPASS1

range: [1,6] units: 1

unique values: 6 missing .: 0/302

tabulation: Freq. Numeric Label

179 1 not at all

54 2 minimal

37 3 mild

24 4 moderate

5 5 severe

3 6 extreme

---------------------------------------------------------------------------------------------------------------------------------------------------------------------------------------------------------------------------------------------------------------

C1_Q24_A2 (sum) C1_Q24_A2

---------------------------------------------------------------------------------------------------------------------------------------------------------------------------------------------------------------------------------------------------------------

type: numeric (double)

label: CPASS1

range: [1,6] units: 1

unique values: 6 missing .: 0/302

tabulation: Freq. Numeric Label

189 1 not at all

51 2 minimal

33 3 mild

22 4 moderate

4 5 severe

3 6 extreme

---------------------------------------------------------------------------------------------------------------------------------------------------------------------------------------------------------------------------------------------------------------

C1_Q24_A3 (sum) C1_Q24_A3

---------------------------------------------------------------------------------------------------------------------------------------------------------------------------------------------------------------------------------------------------------------

type: numeric (double)

label: CPASS1

range: [1,6] units: 1

unique values: 6 missing .: 0/302

tabulation: Freq. Numeric Label

199 1 not at all

50 2 minimal

30 3 mild

16 4 moderate

5 5 severe

2 6 extreme

---------------------------------------------------------------------------------------------------------------------------------------------------------------------------------------------------------------------------------------------------------------

C1_Q24_A4 (sum) C1_Q24_A4

---------------------------------------------------------------------------------------------------------------------------------------------------------------------------------------------------------------------------------------------------------------

type: numeric (double)

label: CPASS1, but 1 nonmissing value is not labeled

range: [0,6] units: 1

unique values: 7 missing .: 0/302

tabulation: Freq. Numeric Label

1 0

224 1 not at all

42 2 minimal

21 3 mild

9 4 moderate

4 5 severe

1 6 extreme

---------------------------------------------------------------------------------------------------------------------------------------------------------------------------------------------------------------------------------------------------------------

C1_Q24_A5 (sum) C1_Q24_A5

---------------------------------------------------------------------------------------------------------------------------------------------------------------------------------------------------------------------------------------------------------------

type: numeric (double)

label: CPASS1

range: [1,5] units: 1

unique values: 5 missing .: 0/302

tabulation: Freq. Numeric Label

239 1 not at all

31 2 minimal

18 3 mild

13 4 moderate

1 5 severe

---------------------------------------------------------------------------------------------------------------------------------------------------------------------------------------------------------------------------------------------------------------

C1_Q24_A6 (sum) C1_Q24_A6

---------------------------------------------------------------------------------------------------------------------------------------------------------------------------------------------------------------------------------------------------------------

type: numeric (double)

label: CPASS1

range: [1,5] units: 1

unique values: 5 missing .: 0/302

tabulation: Freq. Numeric Label

244 1 not at all

39 2 minimal

9 3 mild

9 4 moderate

1 5 severe

---------------------------------------------------------------------------------------------------------------------------------------------------------------------------------------------------------------------------------------------------------------

C1_Q24_A7 (sum) C1_Q24_A7

---------------------------------------------------------------------------------------------------------------------------------------------------------------------------------------------------------------------------------------------------------------

type: numeric (double)

label: CPASS1

range: [1,5] units: 1

unique values: 5 missing .: 0/302

tabulation: Freq. Numeric Label

248 1 not at all

31 2 minimal

17 3 mild

4 4 moderate

2 5 severe

---------------------------------------------------------------------------------------------------------------------------------------------------------------------------------------------------------------------------------------------------------------

C1_Q24_A8 (sum) C1_Q24_A8

---------------------------------------------------------------------------------------------------------------------------------------------------------------------------------------------------------------------------------------------------------------

type: numeric (double)

label: CPASS1

range: [1,4] units: 1

unique values: 4 missing .: 0/302

tabulation: Freq. Numeric Label

257 1 not at all

26 2 minimal

12 3 mild

7 4 moderate

---------------------------------------------------------------------------------------------------------------------------------------------------------------------------------------------------------------------------------------------------------------

C1_Q24_A9 (sum) C1_Q24_A9

---------------------------------------------------------------------------------------------------------------------------------------------------------------------------------------------------------------------------------------------------------------

type: numeric (double)

label: CPASS1

range: [1,5] units: 1

unique values: 5 missing .: 0/302

tabulation: Freq. Numeric Label

257 1 not at all

26 2 minimal

13 3 mild

5 4 moderate

1 5 severe

---------------------------------------------------------------------------------------------------------------------------------------------------------------------------------------------------------------------------------------------------------------

C1_Q24_A10 (sum) C1_Q24_A10

---------------------------------------------------------------------------------------------------------------------------------------------------------------------------------------------------------------------------------------------------------------

type: numeric (double)

label: CPASS1

range: [1,5] units: 1

unique values: 5 missing .: 0/302

tabulation: Freq. Numeric Label

257 1 not at all

27 2 minimal

14 3 mild

3 4 moderate

1 5 severe

---------------------------------------------------------------------------------------------------------------------------------------------------------------------------------------------------------------------------------------------------------------

C1_Q25_B1 (sum) C1_Q25_B1

---------------------------------------------------------------------------------------------------------------------------------------------------------------------------------------------------------------------------------------------------------------

type: numeric (double)

range: [0,0] units: 1

unique values: 1 missing .: 0/302

tabulation: Freq. Value

302 0

---------------------------------------------------------------------------------------------------------------------------------------------------------------------------------------------------------------------------------------------------------------

C1_Q25_B2 (sum) C1_Q25_B2

---------------------------------------------------------------------------------------------------------------------------------------------------------------------------------------------------------------------------------------------------------------

type: numeric (double)

range: [0,0] units: 1

unique values: 1 missing .: 0/302

tabulation: Freq. Value

302 0

---------------------------------------------------------------------------------------------------------------------------------------------------------------------------------------------------------------------------------------------------------------

C1_Q25_B3 (sum) C1_Q25_B3

---------------------------------------------------------------------------------------------------------------------------------------------------------------------------------------------------------------------------------------------------------------

type: numeric (double)

range: [0,0] units: 1

unique values: 1 missing .: 0/302

tabulation: Freq. Value

302 0

---------------------------------------------------------------------------------------------------------------------------------------------------------------------------------------------------------------------------------------------------------------

C1_Q25_B4 (sum) C1_Q25_B4

---------------------------------------------------------------------------------------------------------------------------------------------------------------------------------------------------------------------------------------------------------------

type: numeric (double)

range: [0,2] units: 1

unique values: 2 missing .: 0/302

tabulation: Freq. Value

301 0

1 2

---------------------------------------------------------------------------------------------------------------------------------------------------------------------------------------------------------------------------------------------------------------

C1_Q25_B5 (sum) C1_Q25_B5

---------------------------------------------------------------------------------------------------------------------------------------------------------------------------------------------------------------------------------------------------------------

type: numeric (double)

range: [0,0] units: 1

unique values: 1 missing .: 0/302

tabulation: Freq. Value

302 0

---------------------------------------------------------------------------------------------------------------------------------------------------------------------------------------------------------------------------------------------------------------

C1_Q25_B6 (sum) C1_Q25_B6

---------------------------------------------------------------------------------------------------------------------------------------------------------------------------------------------------------------------------------------------------------------

type: numeric (double)

range: [0,.5] units: .1

unique values: 2 missing .: 0/302

tabulation: Freq. Value

301 0

1 .5

---------------------------------------------------------------------------------------------------------------------------------------------------------------------------------------------------------------------------------------------------------------

C1_Q25_B7 (sum) C1_Q25_B7

---------------------------------------------------------------------------------------------------------------------------------------------------------------------------------------------------------------------------------------------------------------

type: numeric (double)

range: [0,13] units: .1

unique values: 5 missing .: 0/302

tabulation: Freq. Value

298 0

1 .5

1 1.5

1 3

1 13

---------------------------------------------------------------------------------------------------------------------------------------------------------------------------------------------------------------------------------------------------------------

C1_Q25_A1 (sum) C1_Q25_A1

---------------------------------------------------------------------------------------------------------------------------------------------------------------------------------------------------------------------------------------------------------------

type: numeric (double)

range: [0,129.5] units: .1

unique values: 66 missing .: 0/302

mean: 15.0103

std. dev: 16.0103

percentiles: 10% 25% 50% 75% 90%

.5 3.5 12 22.5 35

---------------------------------------------------------------------------------------------------------------------------------------------------------------------------------------------------------------------------------------------------------------

C1_Q25_A2 (sum) C1_Q25_A2

---------------------------------------------------------------------------------------------------------------------------------------------------------------------------------------------------------------------------------------------------------------

type: numeric (double)

range: [0,375] units: .01

unique values: 91 missing .: 0/302

mean: 26.5776

std. dev: 27.4606

percentiles: 10% 25% 50% 75% 90%

5 14 23.75 31.5 45

---------------------------------------------------------------------------------------------------------------------------------------------------------------------------------------------------------------------------------------------------------------

C1_Q25_A3 (sum) C1_Q25_A3

---------------------------------------------------------------------------------------------------------------------------------------------------------------------------------------------------------------------------------------------------------------

type: numeric (double)

range: [0,125] units: .1

unique values: 80 missing .: 0/302

mean: 21.8844

std. dev: 22.2357

percentiles: 10% 25% 50% 75% 90%

3 8 16 26.5 40

---------------------------------------------------------------------------------------------------------------------------------------------------------------------------------------------------------------------------------------------------------------

C1_Q25_A4 (sum) C1_Q25_A4

---------------------------------------------------------------------------------------------------------------------------------------------------------------------------------------------------------------------------------------------------------------

type: numeric (double)

range: [0,125] units: .1

unique values: 58 missing .: 0/302

mean: 11.2765

std. dev: 14.4846

percentiles: 10% 25% 50% 75% 90%

.5 2 7 15 26

---------------------------------------------------------------------------------------------------------------------------------------------------------------------------------------------------------------------------------------------------------------

C1_Q25_A5 (sum) C1_Q25_A5

---------------------------------------------------------------------------------------------------------------------------------------------------------------------------------------------------------------------------------------------------------------

type: numeric (double)

range: [0,150] units: .1

unique values: 42 missing .: 0/302

mean: 5.97517

std. dev: 13.5058

percentiles: 10% 25% 50% 75% 90%

0 0 1.5 7 15.5

---------------------------------------------------------------------------------------------------------------------------------------------------------------------------------------------------------------------------------------------------------------

C1_Q25_A6 (sum) C1_Q25_A6

---------------------------------------------------------------------------------------------------------------------------------------------------------------------------------------------------------------------------------------------------------------

type: numeric (double)

range: [0,85] units: .1

unique values: 27 missing .: 0/302

mean: 2.19702

std. dev: 7.03681

percentiles: 10% 25% 50% 75% 90%

0 0 0 1.5 6

---------------------------------------------------------------------------------------------------------------------------------------------------------------------------------------------------------------------------------------------------------------

C1_Q25_A7 (sum) C1_Q25_A7

---------------------------------------------------------------------------------------------------------------------------------------------------------------------------------------------------------------------------------------------------------------

type: numeric (double)

range: [0,55] units: .1

unique values: 15 missing .: 0/302

mean: .738411

std. dev: 3.91451

percentiles: 10% 25% 50% 75% 90%

0 0 0 0 1.5

---------------------------------------------------------------------------------------------------------------------------------------------------------------------------------------------------------------------------------------------------------------

C1_Q25_A8 (sum) C1_Q25_A8

---------------------------------------------------------------------------------------------------------------------------------------------------------------------------------------------------------------------------------------------------------------

type: numeric (double)

range: [0,18] units: .1

unique values: 11 missing .: 0/302

mean: .178808

std. dev: 1.2387

percentiles: 10% 25% 50% 75% 90%

0 0 0 0 0

---------------------------------------------------------------------------------------------------------------------------------------------------------------------------------------------------------------------------------------------------------------

C1_Q25_A9 (sum) C1_Q25_A9

---------------------------------------------------------------------------------------------------------------------------------------------------------------------------------------------------------------------------------------------------------------

type: numeric (double)

range: [0,1] units: .1

unique values: 3 missing .: 0/302

tabulation: Freq. Value

296 0

5 .5

1 1

---------------------------------------------------------------------------------------------------------------------------------------------------------------------------------------------------------------------------------------------------------------

C1_Q25_A10 (sum) C1_Q25_A10

---------------------------------------------------------------------------------------------------------------------------------------------------------------------------------------------------------------------------------------------------------------

type: numeric (double)

range: [0,11] units: .1

unique values: 4 missing .: 0/302

tabulation: Freq. Value

299 0

1 .5

1 10

1 11

---------------------------------------------------------------------------------------------------------------------------------------------------------------------------------------------------------------------------------------------------------------

C2_Q1_B1 (sum) C2_Q1_B1

---------------------------------------------------------------------------------------------------------------------------------------------------------------------------------------------------------------------------------------------------------------

type: numeric (double)

label: CPASS1

range: [1,5] units: 1

unique values: 5 missing .: 0/302

tabulation: Freq. Numeric Label

232 1 not at all

44 2 minimal

16 3 mild

8 4 moderate

2 5 severe

---------------------------------------------------------------------------------------------------------------------------------------------------------------------------------------------------------------------------------------------------------------

C2_Q1_B2 (sum) C2_Q1_B2

---------------------------------------------------------------------------------------------------------------------------------------------------------------------------------------------------------------------------------------------------------------

type: numeric (double)

label: CPASS1

range: [1,6] units: 1

unique values: 6 missing .: 0/302

tabulation: Freq. Numeric Label

226 1 not at all

41 2 minimal

20 3 mild

11 4 moderate

3 5 severe

1 6 extreme

---------------------------------------------------------------------------------------------------------------------------------------------------------------------------------------------------------------------------------------------------------------

C2_Q1_B3 (sum) C2_Q1_B3

---------------------------------------------------------------------------------------------------------------------------------------------------------------------------------------------------------------------------------------------------------------

type: numeric (double)

label: CPASS1, but 1 nonmissing value is not labeled

range: [0,5] units: 1

unique values: 6 missing .: 0/302

tabulation: Freq. Numeric Label

1 0

228 1 not at all

39 2 minimal

20 3 mild

12 4 moderate

2 5 severe

---------------------------------------------------------------------------------------------------------------------------------------------------------------------------------------------------------------------------------------------------------------

C2_Q1_B4 (sum) C2_Q1_B4

---------------------------------------------------------------------------------------------------------------------------------------------------------------------------------------------------------------------------------------------------------------

type: numeric (double)

label: CPASS1, but 1 nonmissing value is not labeled

range: [0,5] units: 1

unique values: 6 missing .: 0/302

tabulation: Freq. Numeric Label

1 0

225 1 not at all

41 2 minimal

24 3 mild

8 4 moderate

3 5 severe

---------------------------------------------------------------------------------------------------------------------------------------------------------------------------------------------------------------------------------------------------------------

C2_Q1_B5 (sum) C2_Q1_B5

---------------------------------------------------------------------------------------------------------------------------------------------------------------------------------------------------------------------------------------------------------------

type: numeric (double)

label: CPASS1, but 1 nonmissing value is not labeled

range: [0,4] units: 1

unique values: 5 missing .: 0/302

tabulation: Freq. Numeric Label

2 0

223 1 not at all

46 2 minimal

18 3 mild

13 4 moderate

---------------------------------------------------------------------------------------------------------------------------------------------------------------------------------------------------------------------------------------------------------------

C2_Q1_B6 (sum) C2_Q1_B6

---------------------------------------------------------------------------------------------------------------------------------------------------------------------------------------------------------------------------------------------------------------

type: numeric (double)

label: CPASS1, but 1 nonmissing value is not labeled

range: [0,6] units: 1

unique values: 7 missing .: 0/302

tabulation: Freq. Numeric Label

1 0

210 1 not at all

46 2 minimal

24 3 mild

16 4 moderate

4 5 severe

1 6 extreme

---------------------------------------------------------------------------------------------------------------------------------------------------------------------------------------------------------------------------------------------------------------

C2_Q1_B7 (sum) C2_Q1_B7

---------------------------------------------------------------------------------------------------------------------------------------------------------------------------------------------------------------------------------------------------------------

type: numeric (double)

label: CPASS1

range: [1,5] units: 1

unique values: 5 missing .: 0/302

tabulation: Freq. Numeric Label

198 1 not at all

49 2 minimal

33 3 mild

17 4 moderate

5 5 severe

---------------------------------------------------------------------------------------------------------------------------------------------------------------------------------------------------------------------------------------------------------------

C2_Q1_A1 (sum) C2_Q1_A1

---------------------------------------------------------------------------------------------------------------------------------------------------------------------------------------------------------------------------------------------------------------

type: numeric (double)

label: CPASS1

range: [1,6] units: 1

unique values: 6 missing .: 0/302

tabulation: Freq. Numeric Label

178 1 not at all

59 2 minimal

31 3 mild

27 4 moderate

5 5 severe

2 6 extreme

---------------------------------------------------------------------------------------------------------------------------------------------------------------------------------------------------------------------------------------------------------------

C2_Q1_A2 (sum) C2_Q1_A2

---------------------------------------------------------------------------------------------------------------------------------------------------------------------------------------------------------------------------------------------------------------

type: numeric (double)

label: CPASS1

range: [1,5] units: 1

unique values: 5 missing .: 0/302

tabulation: Freq. Numeric Label

194 1 not at all

55 2 minimal

28 3 mild

18 4 moderate

7 5 severe

---------------------------------------------------------------------------------------------------------------------------------------------------------------------------------------------------------------------------------------------------------------

C2_Q1_A3 (sum) C2_Q1_A3

---------------------------------------------------------------------------------------------------------------------------------------------------------------------------------------------------------------------------------------------------------------

type: numeric (double)

label: CPASS1

range: [1,5] units: 1

unique values: 5 missing .: 0/302

tabulation: Freq. Numeric Label

198 1 not at all

53 2 minimal

29 3 mild

20 4 moderate

2 5 severe

---------------------------------------------------------------------------------------------------------------------------------------------------------------------------------------------------------------------------------------------------------------

C2_Q1_A4 (sum) C2_Q1_A4

---------------------------------------------------------------------------------------------------------------------------------------------------------------------------------------------------------------------------------------------------------------

type: numeric (double)

label: CPASS1, but 1 nonmissing value is not labeled

range: [0,6] units: 1

unique values: 7 missing .: 0/302

tabulation: Freq. Numeric Label

1 0

213 1 not at all

50 2 minimal

25 3 mild

10 4 moderate

2 5 severe

1 6 extreme

---------------------------------------------------------------------------------------------------------------------------------------------------------------------------------------------------------------------------------------------------------------

C2_Q1_A5 (sum) C2_Q1_A5

---------------------------------------------------------------------------------------------------------------------------------------------------------------------------------------------------------------------------------------------------------------

type: numeric (double)

label: CPASS1

range: [1,5] units: 1

unique values: 5 missing .: 0/302

tabulation: Freq. Numeric Label

221 1 not at all

43 2 minimal

27 3 mild

9 4 moderate

2 5 severe

---------------------------------------------------------------------------------------------------------------------------------------------------------------------------------------------------------------------------------------------------------------

C2_Q1_A6 (sum) C2_Q1_A6

---------------------------------------------------------------------------------------------------------------------------------------------------------------------------------------------------------------------------------------------------------------

type: numeric (double)

label: CPASS1

range: [1,6] units: 1

unique values: 5 missing .: 0/302

tabulation: Freq. Numeric Label

236 1 not at all

40 2 minimal

19 3 mild

5 4 moderate

2 6 extreme

---------------------------------------------------------------------------------------------------------------------------------------------------------------------------------------------------------------------------------------------------------------

C2_Q1_A7 (sum) C2_Q1_A7

---------------------------------------------------------------------------------------------------------------------------------------------------------------------------------------------------------------------------------------------------------------

type: numeric (double)

label: CPASS1

range: [1,6] units: 1

unique values: 6 missing .: 0/302

tabulation: Freq. Numeric Label

233 1 not at all

40 2 minimal

22 3 mild

4 4 moderate

1 5 severe

2 6 extreme

---------------------------------------------------------------------------------------------------------------------------------------------------------------------------------------------------------------------------------------------------------------

C2_Q1_A8 (sum) C2_Q1_A8

---------------------------------------------------------------------------------------------------------------------------------------------------------------------------------------------------------------------------------------------------------------

type: numeric (double)

label: CPASS1, but 1 nonmissing value is not labeled

range: [0,6] units: 1

unique values: 6 missing .: 0/302

tabulation: Freq. Numeric Label

2 0

237 1 not at all

39 2 minimal

18 3 mild

5 4 moderate

1 6 extreme

---------------------------------------------------------------------------------------------------------------------------------------------------------------------------------------------------------------------------------------------------------------

C2_Q1_A9 (sum) C2_Q1_A9

---------------------------------------------------------------------------------------------------------------------------------------------------------------------------------------------------------------------------------------------------------------

type: numeric (double)

label: CPASS1

range: [1,6] units: 1

unique values: 5 missing .: 0/302

tabulation: Freq. Numeric Label

243 1 not at all

34 2 minimal

17 3 mild

7 4 moderate

1 6 extreme

---------------------------------------------------------------------------------------------------------------------------------------------------------------------------------------------------------------------------------------------------------------

C2_Q1_A10 (sum) C2_Q1_A10

---------------------------------------------------------------------------------------------------------------------------------------------------------------------------------------------------------------------------------------------------------------

type: numeric (double)

label: CPASS1

range: [1,4] units: 1

unique values: 4 missing .: 0/302

tabulation: Freq. Numeric Label

251 1 not at all

30 2 minimal

17 3 mild

4 4 moderate

---------------------------------------------------------------------------------------------------------------------------------------------------------------------------------------------------------------------------------------------------------------

C2_Q2_B1 (sum) C2_Q2_B1

---------------------------------------------------------------------------------------------------------------------------------------------------------------------------------------------------------------------------------------------------------------

type: numeric (double)

label: CPASS1

range: [1,5] units: 1

unique values: 5 missing .: 0/302

tabulation: Freq. Numeric Label

256 1 not at all

27 2 minimal

6 3 mild

11 4 moderate

2 5 severe
[truncated: 698,346 more chars]
