# Supplementary figures and images for: Associated factors with Premenstrual syndrome and Premenstrual dysphoric disorder among female medical students: A cross-sectional study
Source: PLoS One. 2023 Jan 26;18(1):e0278702. doi: 10.1371/journal.pone.0278702 (PMC9879477; doi:10.1371/journal.pone.0278702)

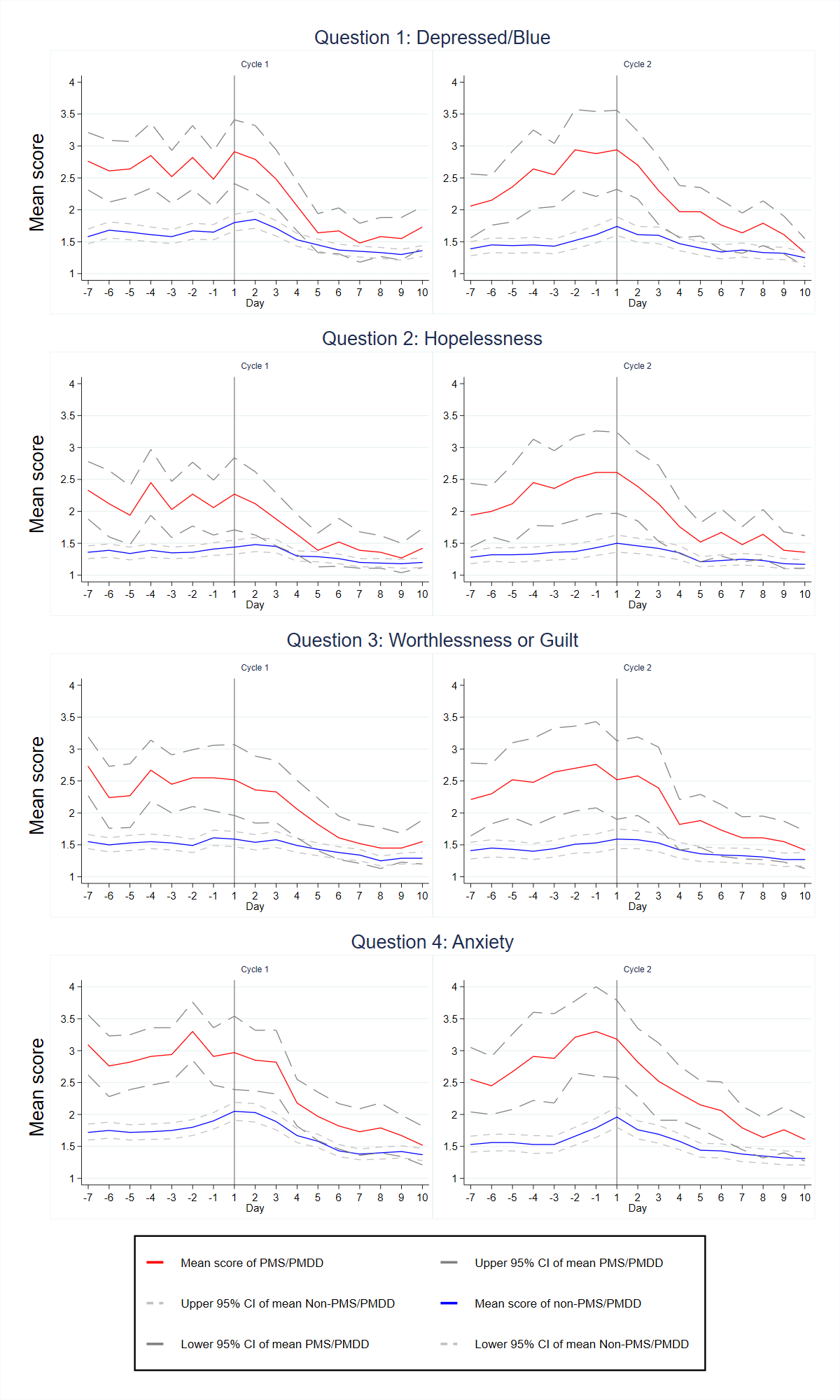

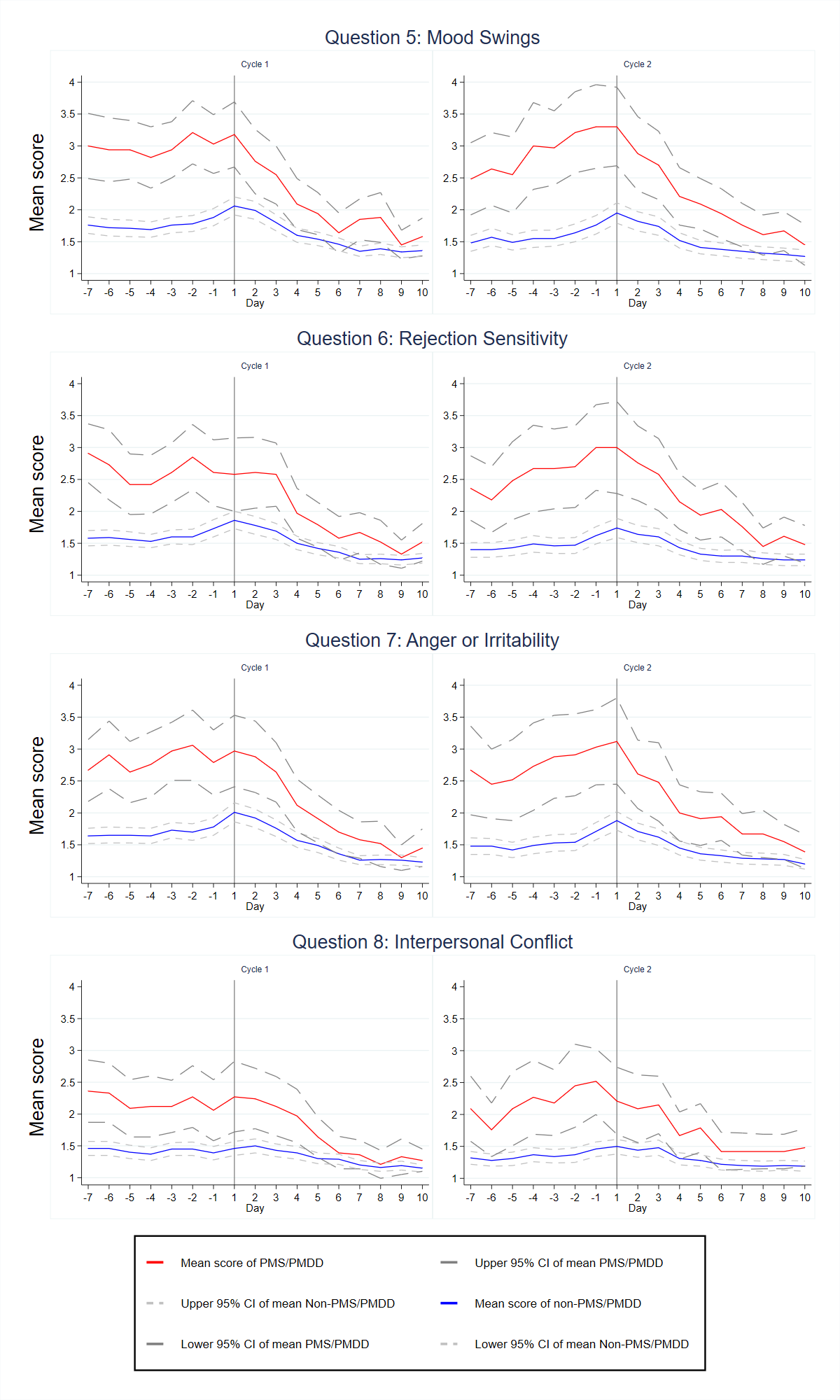

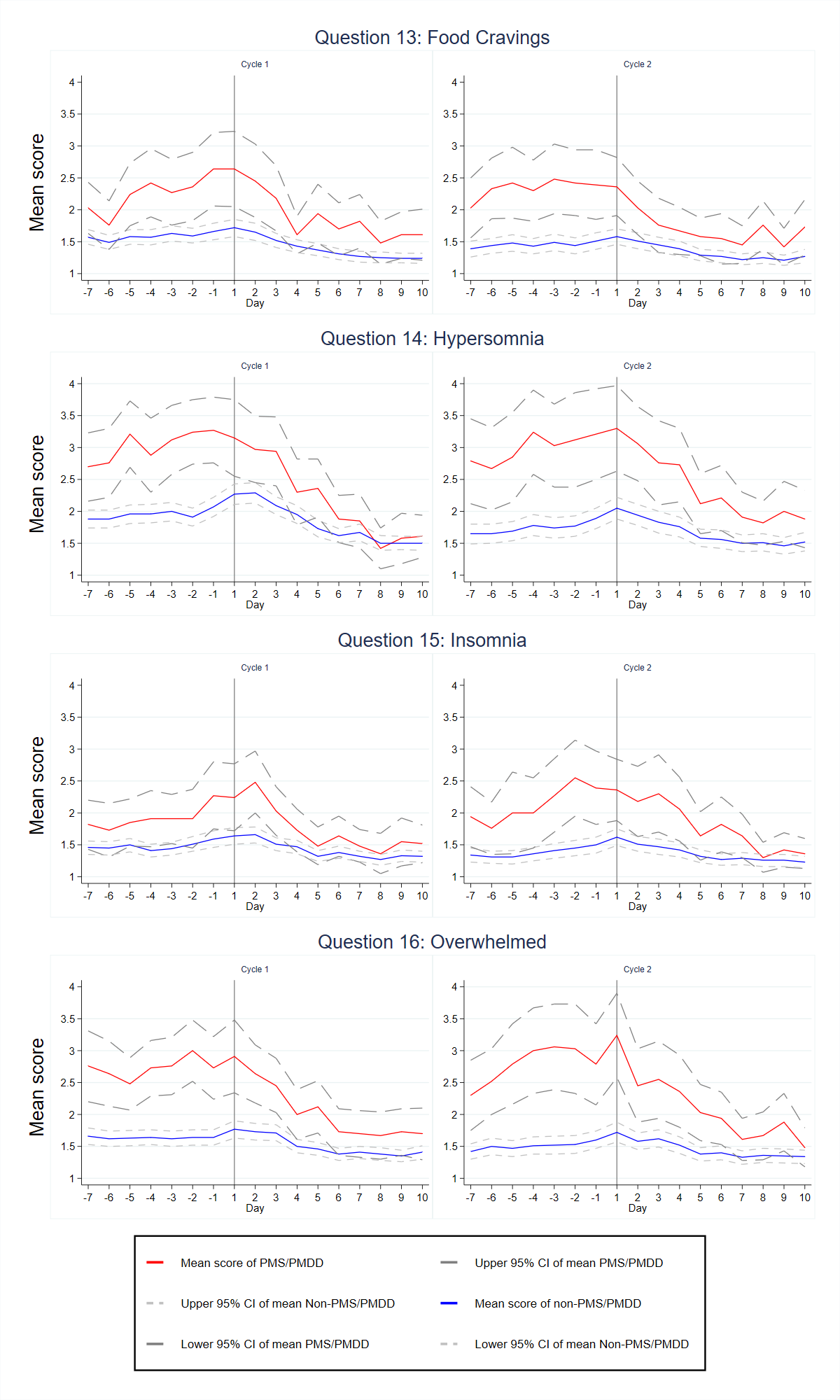

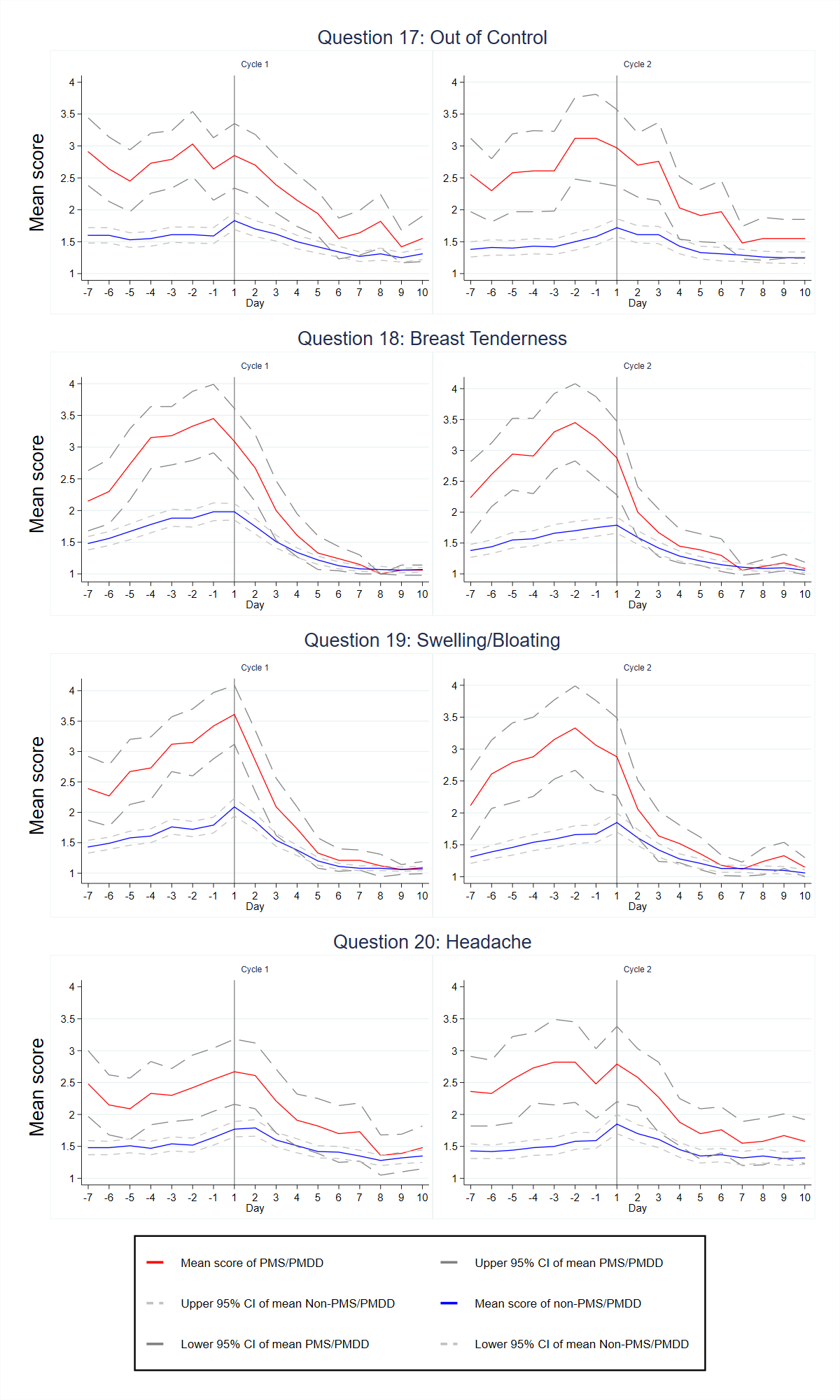

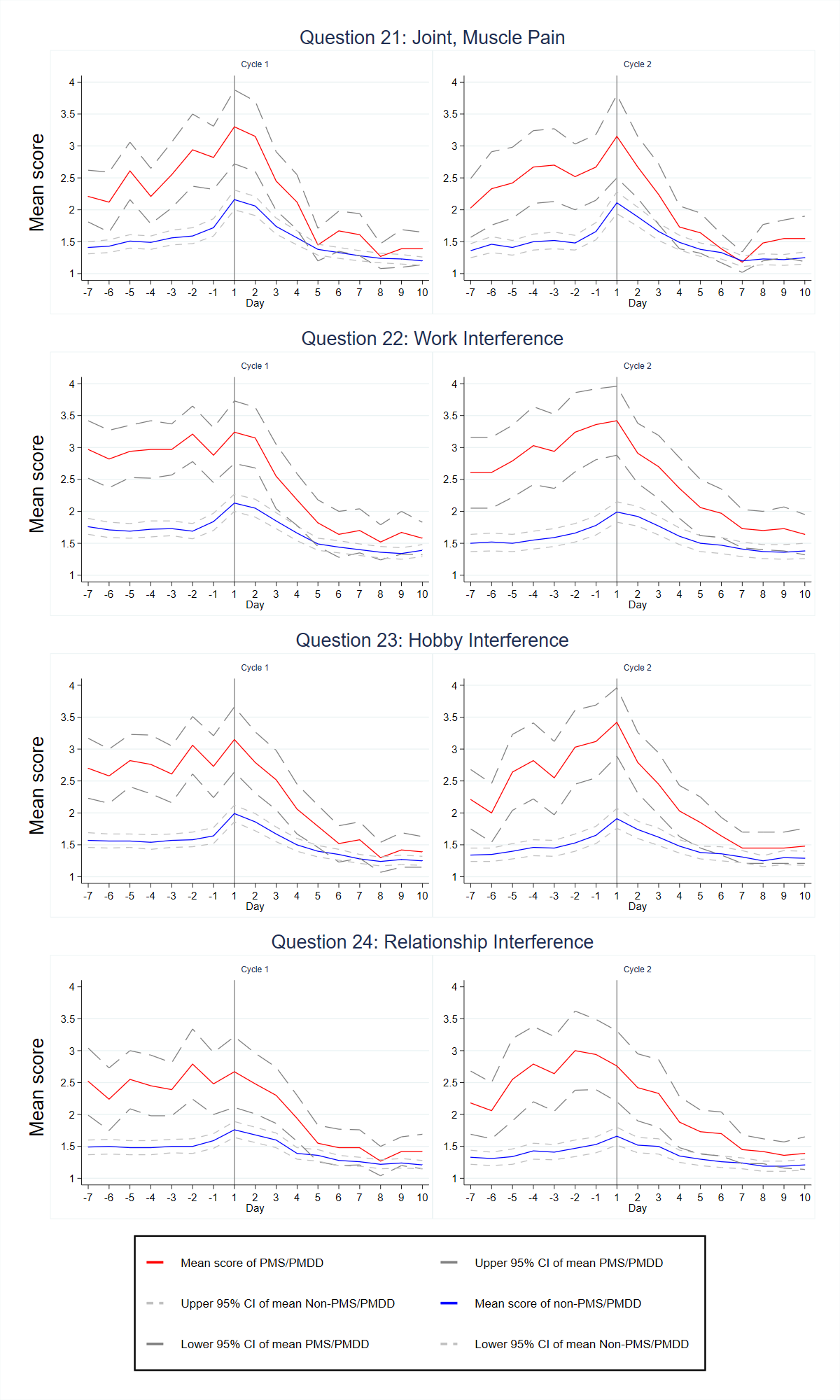


**S1 Figure.** Fluctuation of mean score of 24 symptom reported via DRSP over two menstrual cycles

Supplement: S1 Data — (ZIP) [file pone.0278702.s001.zip › S1. Fig.docx]

**S2 Figure.** Percentage of moderate or severe symptoms screened by PSST and re-test PSST


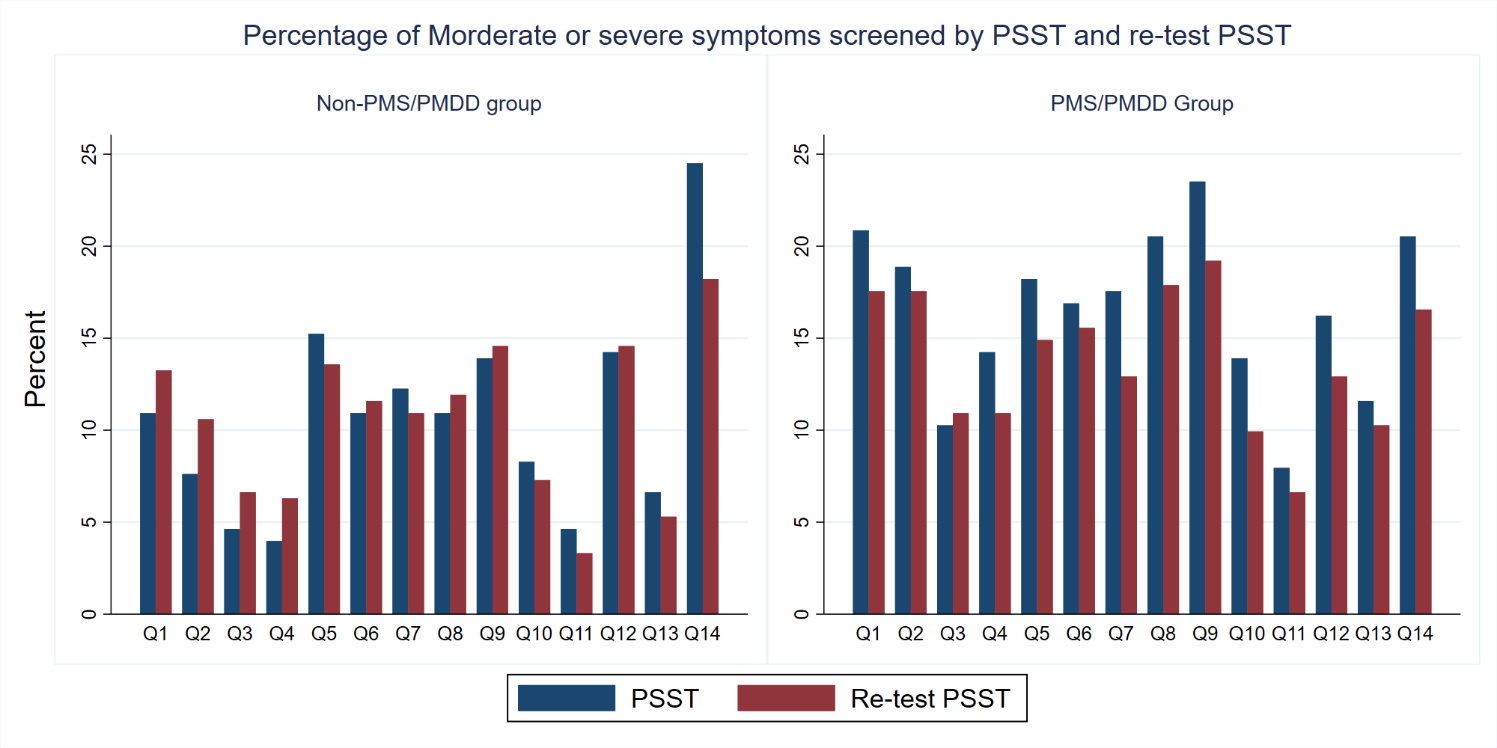

Supplement: S1 Data — (ZIP) [file pone.0278702.s001.zip › S2. Fig.docx]

**S3 Figure.** The Scree plot of eigenvalues based on the principle component analysis (PCA)


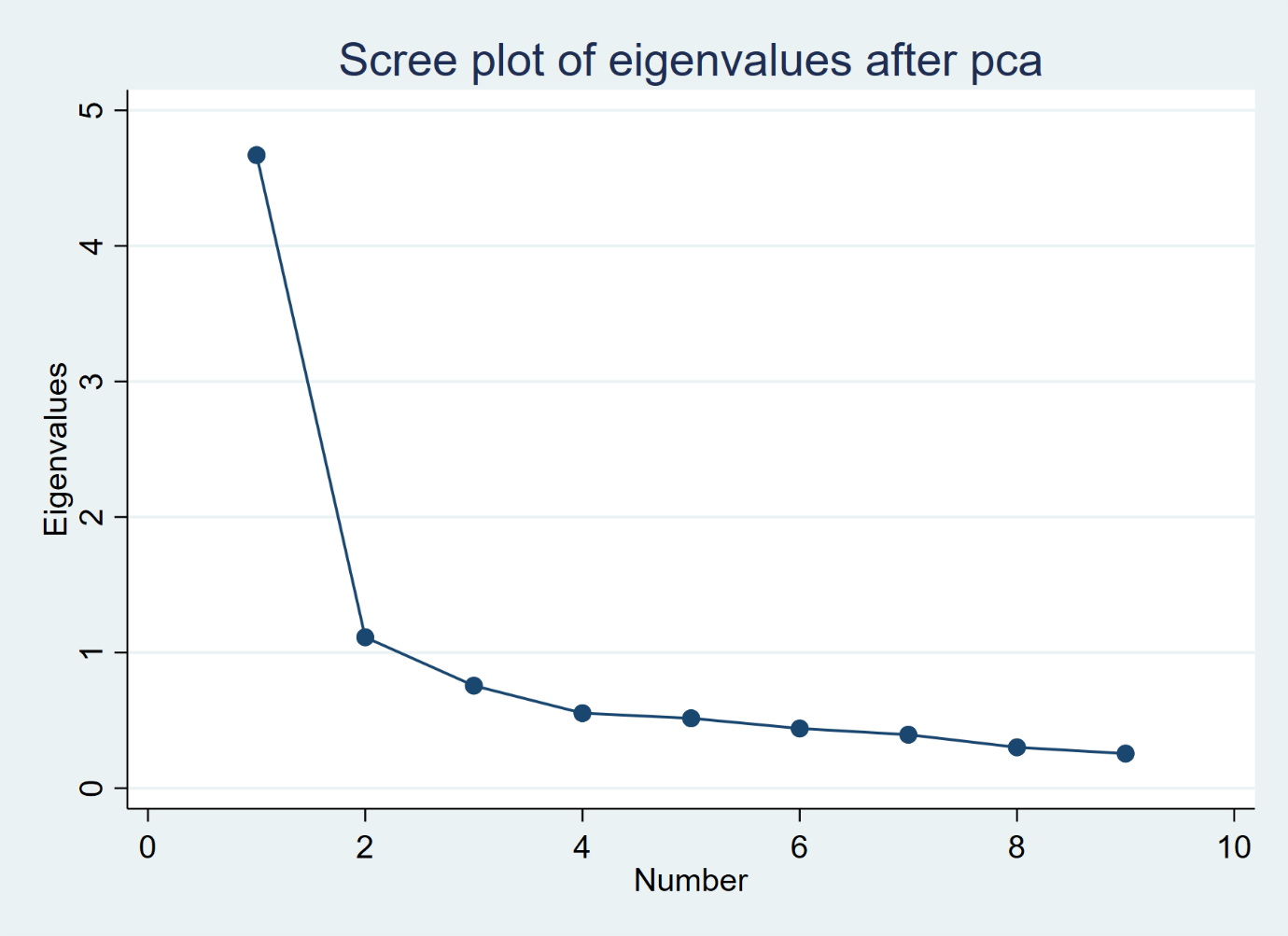

Supplement: S1 Data — (ZIP) [file pone.0278702.s001.zip › S3. Fig.docx]
